# *Meta*-metallation of *N*,*N*-dimethylaniline: Contrasting direct sodium-mediated zincation with indirect sodiation-dialkylzinc co-complexation

**DOI:** 10.3762/bjoc.7.144

**Published:** 2011-09-06

**Authors:** David R Armstrong, Liam Balloch, Eva Hevia, Alan R Kennedy, Robert E Mulvey, Charles T O'Hara, Stuart D Robertson

**Affiliations:** 1 WestCHEM, Department of Pure and Applied Chemistry, University of Strathclyde, Glasgow, G1 1XL, United Kingdom

**Keywords:** alkali metal, crystal structure, isomerisation, metallation, zincation

## Abstract

Previously we reported that direct zincation of *N*,*N*-dimethylaniline by the mixed-metal zincate reagent **1** ((TMEDA)Na(TMP)(*t*-Bu)Zn(*t*-Bu)) surprisingly led to *meta*-metallation (zincation) of the aniline, as manifested in the crystalline complex **2** ((TMEDA)Na(TMP)(*m*-C_6_H_4_-NMe_2_)Zn(*t*-Bu)), and that iodination of these isolated crystals produced the *meta*-isomer *N*,*N*-dimethyl-3-iodoaniline quantitatively. Completing the study here we find that treating the reaction solution with iodine produces a 72% conversion and results in a mixture of regioisomers of *N*,*N*-dimethyliodoaniline, with the *meta*-isomer still the major product (*ortho*:*meta*:*para* ratio, 6:73:21), as determined by NMR. In contrast to this bimetallic method, sodiation of *N*,*N*-dimethylaniline with *n*-BuNa produced the dimeric, *ortho*-sodiated complex **3** (((TMEDA)Na(*o*-C_6_H_4_-NMe_2_))_2_), as characterised by X-ray crystallography and NMR. No regioisomers were observed in the reaction solution. Introducing *t*-Bu_2_Zn to this reaction solution afforded a cocrystalline product in the solid-state, composed of the bis-anilide **4** ((TMEDA)Na(*o*-C_6_H_4_-NMe_2_)_2_Zn(*t*-Bu)) and the Me_2_N–C cleavage product **5** ({(TMEDA)_2_Na}^+^{(*t*-Bu_2_Zn)_2_(µ-NMe_2_)}^−^), which was characterised by X-ray crystallography. NMR studies of the reaction mixture that produces **4** and **5** revealed one additional species, but the mixture as a whole contained only *ortho*-species and a trace amount of *para*-species as established by iodine quenching. In an indirect variation of the bimetallic reaction, TMP(H) was added at room temperature to the reaction mixture that afforded **4** and **5**. This gave the crystalline product **6** ((TMEDA)Na(TMP)(*o*-C_6_H_4_-NMe_2_)Zn(*t*-Bu)), the *ortho*-isomer of the *meta*-complex **2**, as determined from X-ray crystallographic and NMR data. Monitoring the regioselectivity of the reaction by iodination revealed a 16.6:1.6:1.0 *ortho*:*meta*:*para* ratio. Interestingly, when the TMP(H) containing solution was heated under reflux for 18 hours more *meta*-isomer was produced (corresponding ratio 3.7:4.2:1.0). It is likely that this change has its origin in a retro reaction that produces the original base **1** as an intermediate. Theoretical calculations at the DFT level using the B3LYP method and the 6-311G** basis set were used to probe the energetics of both monometallic and bimetallic systems. In accord with the experimental results, it was found that *ortho*-metallation was favoured by sodiation; whereas *meta*- (closely followed by *para*-) metallation was favoured by direct sodium-mediated zincation.

## Introduction

While the metallation reaction remains an essential tool for constructing substituted aromatic compounds [1–2], the quest for new improved reagents capable of selectively abstracting hydrogen from organic substrates continues. Routinely, organolithium reagents have been employed for this purpose with the high electropositivity of lithium affording polar, reactive C^δ−^–Li^δ+^ bonds proficient in metallating C–H bonds in organic, especially aromatic and heteroaromatic, compounds [3–5]. Although, alkyllithium or lithium amide reagents are regarded as the preferred metallating agents for many transformations, there are major limitations associated with their use, with prominent drawbacks including poor functional group tolerance and low stability of the developing lithio-intermediates necessitating the use of sub-ambient temperatures to effect the desired reactions and avoid decomposition or side reactions. In recent years, as improvements on the existing metallating agents have been sought, research groups around the world have investigated innovative bimetallic alternatives to the customary homometallic reagents.

As part of these innovations the zinc–hydrogen exchange reaction has undergone a significant revolution in recent times propelling it into the spotlight. Despite finding utility in various types of organic reaction [6], simple zinc reagents (alkyls, amides) are kinetically sluggish bases and consequently ineffectual for deprotonative metallation [7]. Nonetheless, when part of an intricate multi-component composition, a striking enhancement in reactivity can be bestowed upon the zinc reagent as a result of cooperative effects between the different components. The work of Knochel uncovered a special reactivity and selectivity that can be realised with a mixed lithium halide–magnesium amide complex sometimes labelled a “turbo-Grignard” reagent [8]. It has been proposed that LiCl breaks up the magnesium amide aggregates allowing more soluble mixed-metal, mixed-anion reagents such as (TMPMgCl·LiCl) to perform regioselective functionalisation of aromatic and heteroaromatic compounds (TMP: 2,2,6,6-tetramethylpiperidide). Modified from their magnesiating “turbo-Grignard” reagents, Knochel’s three component system ((TMP)_2_Zn·2MgCl_2_·2LiCl) allows direct zincation of functionalised arenes and heteroarenes [9–10]. However, common limitations have been noted for the use of this reagent with some electron poor and heterocyclic compounds which suffer from the drawback of limited regioselectivity, whilst several activated aromatic compounds bearing sensitive functional groups necessitate the use of sub-ambient temperatures. These problems can be circumvented by the use of the commercially available, more selective zinc base TMPZnCl·LiCl that executes chemo- and regioselective zincation of aryl and heteroaryl substrates containing sensitive groups typically at ambient temperature [11] and on the multi-gram scale with metallation rates comparable to those obtained for small scale processes [12]. Alkali metal zincates have also been given consideration, principally two-component dialkyl-amido zincates MZn(NR_2_)R′_2_. The reagent “(LiZn(TMP)(*t*-Bu)_2_)”, introduced by Kondo and Uchiyama, is capable of directly zincating a range of similar substrates [13–14]. The sodium zincate **1** ((TMEDA)Na(TMP)(*t*-Bu)Zn(*t*-Bu); TMEDA: *N*,*N*,*N′*,*N′*-tetramethylethylenediamine), reported by our group, is a potent and versatile zincating agent for a wide range of aromatic molecules usually inert towards orthodox organozinc reagents including benzene [15] and naphthalene [16]. These studies – that have been structurally supported by X-ray crystallography in tandem with NMR spectroscopy – have uncovered the chemical synergy that these mixed-metal alternatives can exhibit, which enabled such reagents to perform special metallation reactions that cannot be reproduced by either of the single-metal components that constitute the mixed-metal reagent. While the alkali metal component is essential for the synergic metallation to follow its course, it is the less electropositive zinc that replaces the departing hydrogen atom, prompting metallations of this type to be best regarded as “Alkali-Metal-Mediated Zincations (AMM*Zn*)” [17].

Directed *ortho*-metallation (D*o*M) is a special sub-category of deprotonative metallation. Defined as a metallation (nearly always lithiation) of an aromatic ring directed towards a position adjacent (*ortho*) to an activating heteroatom-containing functional group, it is widely regarded as the number one methodology for constructing regiospecifically substituted aromatic rings [18–19]. However, by channelling the special synergic chemistry of sodium TMP-zincates, it is possible to overcome D*o*M effects and direct metallation to more remote positions. Of particular interest to this work, we previously reported that *N*,*N*-dimethylaniline can be monozincated by the base **1** at the normally inaccessible *meta*-position [20] (while complexation of *N*,*N*-dimethylaniline with Cr(CO)_3_ leads to a mixture of *ortho*-, *meta*- and *para*-deprotonation upon lithiation [21], other situations of *meta*-deprotonation require a special combination of substituents on the aromatic ring. Such situations, which because of the multiple substitution limit the number of sites available for deprotonation, are usually dictated by steric constraints and therefore should be clearly distinguished from monosubstituted aromatic rings in which *meta*-deprotonation is far more difficult to attain [22]). Another example of a synergic metallation can be found with the alkyl arene toluene. With conventional metallating reagents such as the alkyllithium BuLi·TMEDA, toluene is normally deprotonated at the methyl (lateral) site to generate the resonance stabilised benzyl “carbanion” (PhCH_2_^−^) [23–24]. In contrast, toluene has been directly zincated by reaction with the heteroleptic sodium zincate **1** to afford a statistical mixture of the *meta*- and *para*-regioisomers of (TMEDA)Na(TMP)(C_6_H_4_-Me)Zn(*t*-Bu) with the methyl substituent remaining intact [25].

**Scheme 1:**
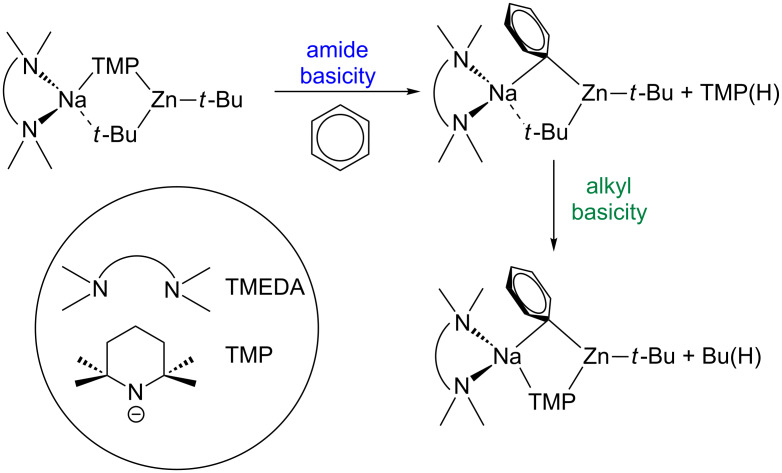
Proposed stepwise mechanism for the zincation of benzene.

The structural insight offered by these findings has been augmented by a complementary theoretical study. Filling in the gaps between the crystallographically characterised starting materials and the thermodynamic final products, Uchiyama and Nobuto have studied and evaluated the possible reaction pathways of the deprotonative metallation of benzene with sodium TMP-zincate **1** computationally [26]. Their DFT studies propose that these distinctive deprotonations proceed via a stepwise mechanism, whereby zincate **1** serves kinetically as an amide base (due to the greater kinetic lability of the Zn–N bonds) with concomitant release of TMP(H) with the formation of a bisalkyl(aryl) zincate intermediate (Scheme 1). In the second step, it follows that the co-existent, strongly acidic N–H bond of TMP(H) is deprotonated by this intermediate to afford the final thermodynamic product of the reaction identified by the aforementioned X-ray crystallographic studies, and isobutane.

Recently, we have sought to identify the structures of the reaction intermediates suggested by the theoretical studies. In addition to a closely related study for the AMM*Zn* of anisole by the analogous lithium TMP-zincate ((THF)Li(TMP)(*t*-Bu)Zn(*t*-Bu)) [27], these mechanistic and structural studies were then extended to the metallation of trifluoromethylbenzene by the bimetallic base **1** [28]. Providing a greater understanding of the mechanisms involved, the reaction was structurally traced by isolation of the kinetic solvent-separated *ortho*-deprotonated product ({(TMEDA)_2_Na}^+^{Zn(*o*-C_6_H_4_-CF_3_)(*t*-Bu)_2_}^−^) before exploring its reactivity towards TMP(H) mirroring the second, multipart step of the AMM*Zn* process. Intriguingly, this second step was found to affect strongly the conclusion, swaying not only the product yield but also the final regioselectivity of the metallation with a complex mixture of *ortho*-, *meta*- and *para*-regioisomeric products observed in solution. These findings provided the first experimental evidence for a two-step mechanism in deprotonative metallations with TMP-zincates but additionally pose the question; “is the unique *meta*-metallation of *N*,*N*-dimethylaniline a consequence of TMP induced isomerisation?” This study attempts to answer this important question.

Herein we investigate in detail the reaction of **1** with *N*,*N*-dimethylaniline, exploring the proposed two-step mechanism and shedding light on the special synergic facet of the AMM*Zn* reaction. We report the synthesis and structural elucidation of *ortho*-metallated *N*,*N*-dimethylaniline complexes **3 (**((TMEDA)Na(*o*-C_6_H_4_-NMe_2_))_2_) and **4** ((TMEDA)Na(*o*-C_6_H_4_-NMe_2_)_2_Zn(*t*-Bu)) prepared by direct metallation with *n*-BuNa followed by (in the case of **4**) co-complexation with *t*-Bu_2_Zn, before surveying the latter’s reactivity towards TMP(H) replicating the second step of the stepwise procedure. Offering increased understanding of the possible mechanisms involved in the reactions of TMP-zincates, the solid-state structural features of an isolated decomposition product **5** ({(TMEDA)_2_Na}^+^{(*t*-Bu_2_Zn)_2_(µ-NMe_2_)}^−^) are also discussed, thus overall providing an illustrative example of the subtle, yet prodigious reactivity of the bimetallic base **1**.

## Results and Discussion

**Figure 1:**
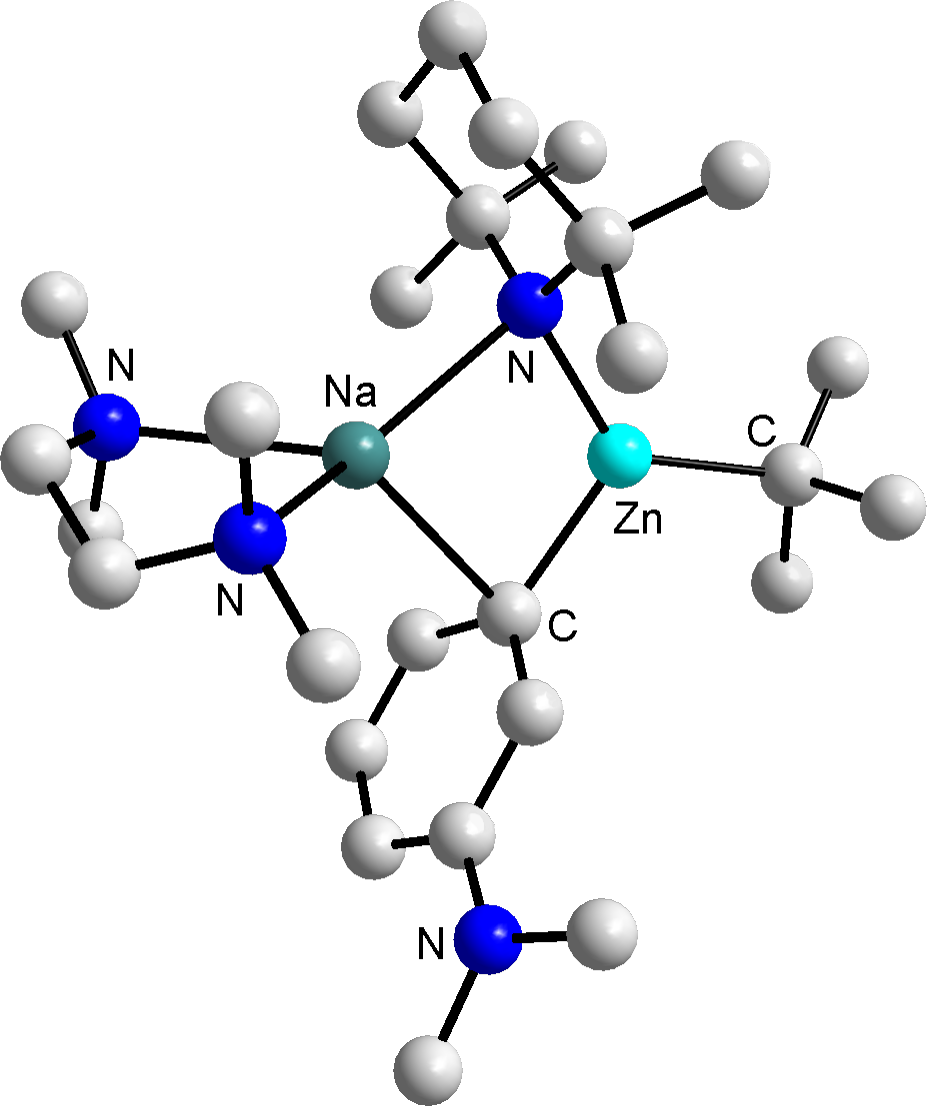
Molecular structure of **2** with selective atom labelling. Hydrogen atoms and minor disorder components are omitted for clarity [20].

**The original direct *meta*-zincation reaction.** In comparison to tertiary amides and *O*-carbamates, anilines are relatively modest metallation directing groups [19,29]. Activation of *N*,*N*-dimethylaniline transpires primarily from the acidifying effect of the N atom; the coordination effect of the N atom is believed to be less important as a result of the conjugation of its lone pair with the π-system of the ring, even so *N*,*N*-dimethylaniline undergoes D*o*M with phenyllithium in poor yield [30] or *n*-butyllithium in good yield under forcing conditions [31]. Varying the metallation agent from these mainstream homometallic species to heterometallic **1** remarkably switched the orientation of the deprotonation to the *meta*-site, with the metallated heterotrianionic product of the reaction **2** ((TMEDA)Na(TMP)(*m*-C_6_H_4_-NMe_2_)Zn(*t*-Bu)) isolated at ambient temperature in a stable crystalline form which allowed its molecular structure to be ascertained by X-ray crystallography (Figure 1).

As reported, a preliminary reaction of isolated crystals of **2** in THF with I_2_ was carried out to establish whether these *meta*-zincated dimethylaniline complexes could be intercepted by electrophiles. ^1^H NMR spectroscopic experiments revealed quantitative iodination and the formation of *N*,*N*-dimethyl-3-iodoaniline. Now we are looking to paint the complete picture of this surprising synergic *meta*-deprotonation of *N*,*N*-dimethylaniline.

**Scheme 2:**
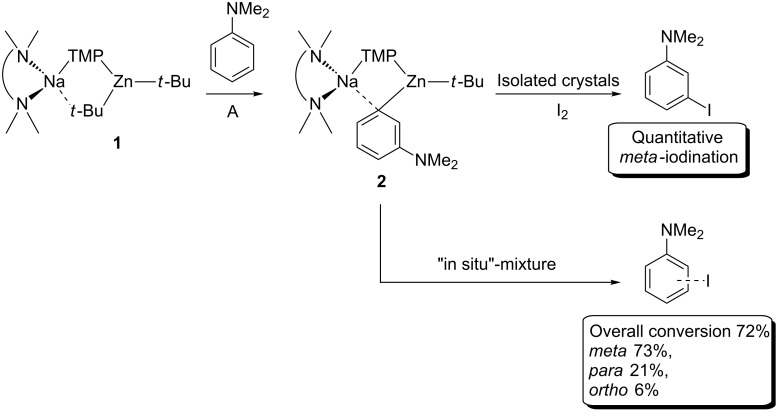
Synergic metallation of *N*,*N*-dimethylaniline (**A)** with sodium TMP-zincate **1 to** produce **2**, which was subsequently quenched with I_2_ to produce iodo-anilines.

**The direct *meta*-zincation reaction: a closer look.** Revisiting the reaction, sodium TMP-zincate **1** was prepared in situ in hexane solution and reacted with one molar equivalent of *N*,*N*-dimethylaniline at room temperature to afford a yellow solution which was subsequently treated with iodine (Scheme 2). NMR spectroscopic analysis (^1^H and ^1^H–^1^H COSY NMR spectra in deuterated benzene) of the crude reaction solution revealed a complex mixture of iodinated products that contained all three possible ring regioisomers for the monometallation of *N*,*N*-dimethylaniline. In agreement with the X-ray crystallographic analysis, the *meta*-isomer was the principal product, but additionally in the aromatic region two doublets at 7.42 and 6.13 ppm were observed for the next most abundant component, i.e., the *para*-product and finally four multiplets at 7.75, 6.99, 6.73, and 6.46 ppm for the minor *ortho*-product in an overall 11.6:3:1 ratio.

**Figure 2:**
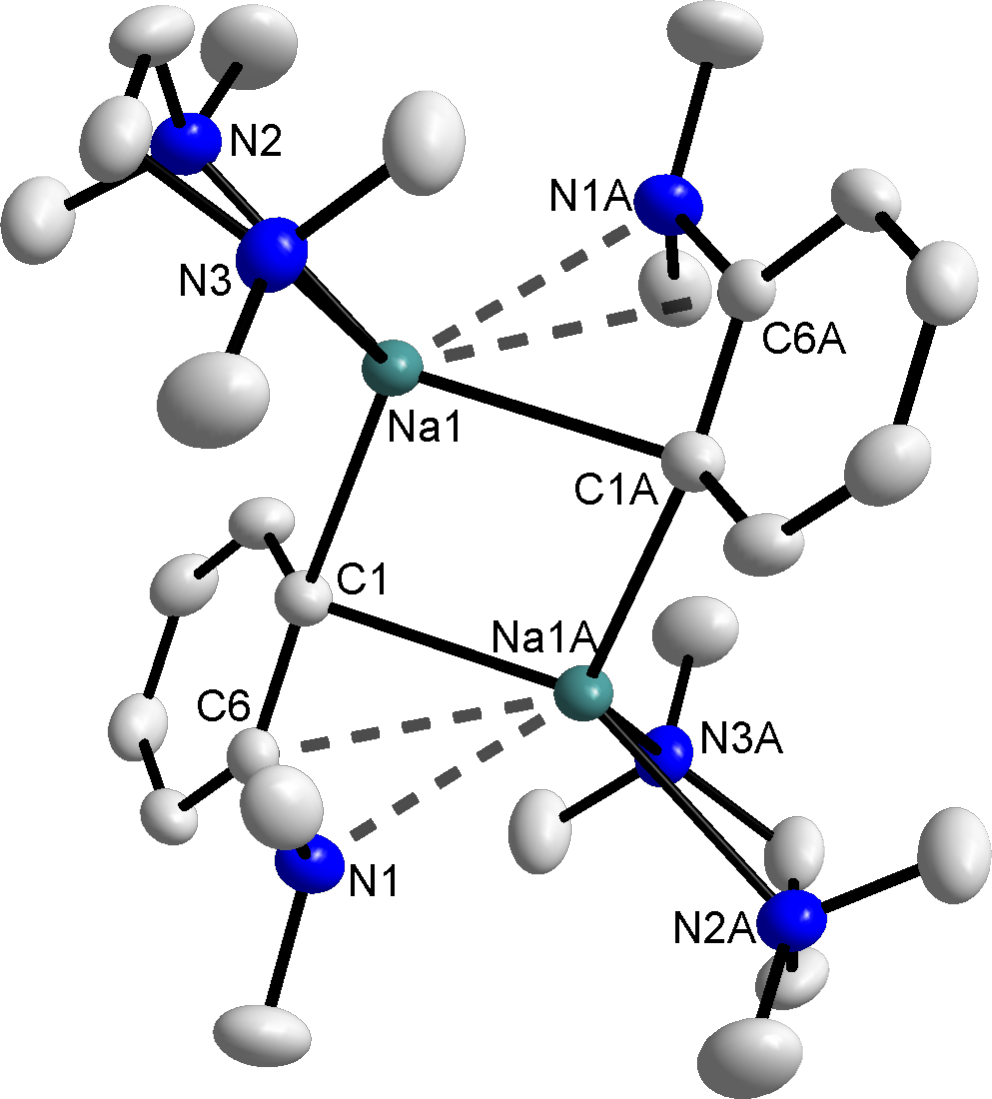
Molecular structure of **3** with selective atom labelling and thermal ellipsoids drawn at the 50% probability level. Hydrogen atoms are omitted for clarity. The long Na···C*_ipso_* and Na···N contacts are highlighted by dashed lines. Symmetry operation to generate equivalent atoms denoted A: 1−x, −y, 1−z. Selected bond distances (Å) and angles (°): Na(1)–N(2) 2.5363(14), Na(1)–N(3) 2.5350(14), Na(1)–C(1) 2.5442(15), Na(1)–C(1A) 2.5903(16), Na(1)···C(6A) 2.9231(15), Na(1)···N(1A) 2.6749(14), N(3)–Na(1)–N(2) 73.37(5), N(3)–Na(1)–C(1) 129.64(5), N(2)–Na(1)–C1) 104.31(5), N(3)–Na(1)–C(1A) 97.45(5), N(2)–Na(1)–C(1A) 150.27(5), C(1)–Na(1)–C(1A) 103.12(4), N(3)–Na(1)–N(1A) 106.68(4), N(2)–Na(1)–N(1A) 99.59(5), C(1)–Na(1)–N(1A) 122.86(5).

**The indirect sequential sodiation-dialkylzinc co-complexation approach.** Closely imitating the constituents of sodium zincate **1**, *N*,*N*-dimethylaniline was *ortho*-sodiated by reaction with *n*-BuNa in hexane at 0 °C to give the product as a light orange precipitate in 64% yield. We employed *n*-BuNa because in our hands NaTMP, with or without the addition of TMEDA, was not sufficiently basic to abstract the *ortho*-hydrogen of *N*,*N*-dimethylaniline. In an attempt to produce crystals of the resulting sodium anilide, the red filtrate (after isolation of the precipitate) was concentrated in vacuo to yield yellow/orange crystals following storage in a refrigerator. X-ray crystallography identified the product as the dimeric, *ortho*-sodiated **3** (((TMEDA)Na(*o*-C_6_H_4_-NMe_2_))_2_) (Figure 2).

Sodiated aniline **3** adopts a simple dimeric centrosymmetric molecular structure based on a four membered (Na_2_(µ-C)_2_) ring, which is strictly planar. Occupying a tetrahedral geometry (mean angle around Na: 109.76°) the sodium atom in **3** is bound to a chelating TMEDA ligand and two deprotonated aniline units, the latter primarily through a close contact with the two *ortho*-deprotonated carbon atoms of the aromatic ring (Na(1)−C(1) 2.5442(15); Na(1)–C(1A) 2.5903(16) Å). In addition to these primary contacts, sodium also engages in longer, weaker secondary contacts with both the *ipso*-carbon and nitrogen atom of the anilide unit (Na(1)···C(6A) 2.9231(15) and Na(1)···N(1A) 2.6749(14) Å). The Na(1)–C(1A) distance is comparable to similar distances in the solvent-free sodium aryl derivative (NaC_6_H_3_–2,6-Mes_2_)_2_ prepared by Power (mean Na–C bond distance of 2.609 Å) [32], whilst they are naturally longer than those in the lithium dimer ({1-(dimethylamino)-8-naphthyl}-lithium·THF)_2_ (2.207(4), 2.223(4) Å) [33]. In this latter compound the lithium atoms are located ca. 0.37 Å above and 1.05 Å below the naphthyl planes. By way of comparison, the sodium atoms in **3** are positioned 0.6565(5) Å above and 1.8248(5) Å below the aryl ring as a result of the increased size of sodium; a trend also reflected in the distance between the averaged main planes of the aromatic rings (1.176(1) Å in **3** and a surprisingly small 0.68 Å between the naphthyl rings of the lithium compound).

For the purposes of NMR spectroscopic analysis, the optimum solubility of **3** was achieved in deuterated cyclohexane solution. The salient observation from the NMR spectra of the solid material and indeed the mother liquor left following its isolation is that there is no sign of any other metallated products/isomers. Therefore the *ortho*-sodiated *N*,*N*-dimethylaniline was the sole metallated product detected during the course of the reaction. ^1^H NMR spectroscopic analysis of a *d*_12_-cyclohexane solution of **3** revealed three multiplets at 7.78, 6.79 and 6.58 ppm representing the *ortho*-deprotonated aromatic ring (overlap at 6.58 ppm between the *ortho*′- and *para*-protons) with the NMe_2_ singlet resonance of the aniline appearing at 2.83 ppm, a shift of 0.12 ppm downfield from that in *N*,*N*-dimethylaniline. Completing the assignment, signals for the TMEDA ligand are found at 2.22 and 2.04 ppm. Emphasising the synthetically limiting reactivity of **3**, it appears to react with THF, while undesired deprotonation of toluene furnished crystals of the known compound TMEDA solvated benzylsodium as confirmed by NMR analysis [34]. Indeed, Slocum et al. have recently developed a selective *ortho*-metallation protocol which permits the retention of the bromine substituent in *para*-bromoanisole by the use of *ortho*-lithiodimethylaniline, which presumably must exhibit a reduced reactivity compared to its sodium analogue **3**, when employed as the metallating agent [35]. Indeed upon storage in a dry box, the shelf life of sodium anilide **3** is no more than a couple of days.

**Scheme 3:**
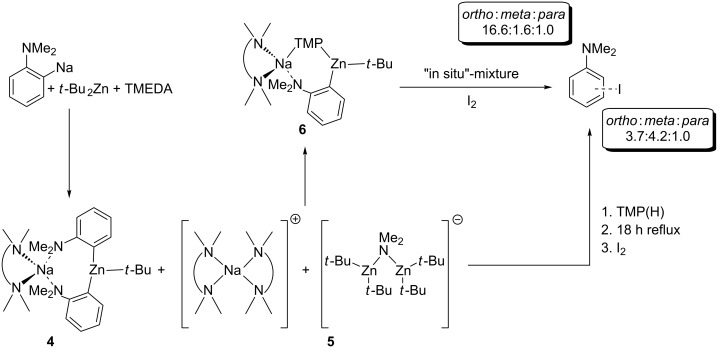
Indirect zincation of *N*,*N*-dimethylaniline producing **4**, **5** and **6**, which was then quenched with I_2_ to produce iodo-anilines.

Continuing with the experimental studies, to introduce the second metal component one molar equivalent of *t*-Bu_2_Zn (dissolved in hexane solution) was added to a suspension of **3** in hexane, which was prepared in situ, and the mixture was stirred at 0 °C for 1 hour and then for a further 2 hours at room temperature (Scheme 3). Following gentle heating of the mixture, colourless crystals were deposited upon slow cooling of the Schlenk tube in a Dewar flask of hot water overnight. X-ray crystallography indicated that this was a 2:1 co-crystalline mixture of two products, **4** ((TMEDA)Na(*o*-C_6_H_4_-NMe_2_)_2_Zn(*t*-Bu)) and **5** ({(TMEDA)_2_Na}^+^{(*t*-Bu_2_Zn)_2_(µ-NMe_2_)}^−^).

**Figure 3:**
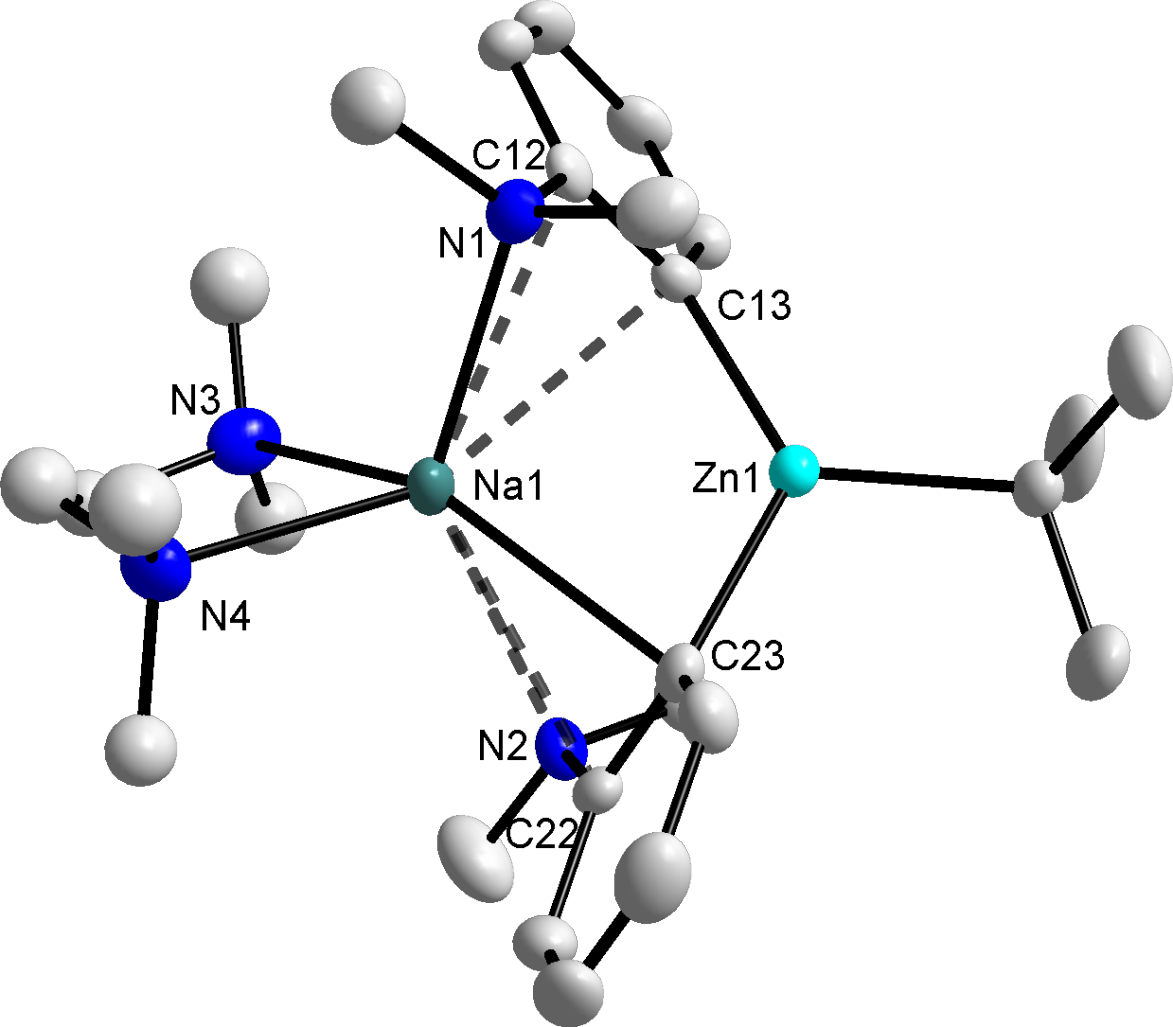
Molecular structure of **4** with selective atom labelling and thermal ellipsoids drawn at the 50% probability level. Hydrogen atoms and disordered component of TMEDA are omitted for clarity. Secondary contacts between sodium and the anilide rings are denoted by dashed lines. Only one of the crystallographically independent molecules is displayed, the parameters for the other are the same within experimental error. Selected bond distances (Å) and angles (°): Zn(1)–C(13) 2.025(3), Zn(1)–C(23) 2.042(3), Na(1)–N(1) 2.663(3), Na(1)···C(12) 2.848(3), Na(1)···C(13) 2.799(3), Na(1)···N(2) 2.851(2), Na(1)···C(22) 2.884(3), Na(1)–C(23) 2.663(3), Na(1)–N(3) 2.473(2), Na(1)–N(4) 2.473(3), C(13)–Zn(1)–C(23) 123.30(11), N(3)–Na(1)–N(4) 74.20(9), N(4)–Na(1)–C(23) 112.74(10), N(3)–Na(1)–C(23) 147.52(9), N(4)–Na(1)–N(1) 102.85(8), N(3)–Na(1)–N(1) 114.85(9), C(23)–Na(1)–N(1) 95.05(9).

Bisaryl **4** (Figure 3) can be considered a mixed-metal product of the co-complexation reaction between **3** and *t*-Bu_2_Zn, despite the incorporation of a second deprotonated organic substrate. Although, a *tert*-butyl group has been formally lost, the formation of **4** may not necessarily be a result of a second deprotonation, with a disproportionation pathway potentially at play. The molecular structure of **4** can be described as a contacted ion-pair with the anionic moiety containing a trigonal planar zinc centre bonded to three carbon atoms, two from the *ortho*-metallated aniline fragments and one from the *tert*-butyl group. In addition to the nitrogen atoms of the chelating TMEDA, the sodium atoms primary coordination sphere is completed by bonding to the nitrogen of one of the anilide units (Na(1)–N(1) 2.663(3) Å) and the *ortho*-deprotonated carbon of the second aromatic ring (Na(1)–C(23) 2.663(3) Å), resulting in sodium adopting a distorted tetrahedral geometry (mean angle around Na: 107.87°). Furthermore, the multihapto-sodium centre engages in weaker, secondary contacts to the *ipso*-carbons of both anilides (Na(1)···C(12) 2.848(3) and Na(1)···C(22) 2.884(3) Å) and additionally, interacts with not only the second deprotonated carbon (Na(1)···C(13) 2.799(3) Å) but also the second aniline N atom (a long Na(1)···N(2) length of 2.851(2) Å compared to the Na–N_TMEDA_ bond lengths of Na(1)–N(3) 2.473(2) and Na(1)–N(4) 2.473(3) Å). Notably, the carbanion which was initially bound to sodium is now directly attached to the more carbophilic zinc as illustrated by the short, strong zinc–carbon σ-bonds (Zn(1)–C(13) 2.025(3), Zn(1)–C(23) 2.042(3)). A search of the Cambridge Structural Database [36] found that the bisaryl model has precedent in alkali-metal zincate chemistry, for example, the previously prepared biscarboxamide ((TMEDA)Li{2-(1-C(O)N(*i*-Pr)_2_)C_6_H_4_}_2_Zn(*t*-Bu)) [37], however the donor ability of the different directing groups limits comparison with **4**. A salient feature of this structure is the bond between lithium and the Lewis basic carbonyl oxygen atoms which close a ten-membered ring, resulting in a contracted Ph–Zn–Ph bond angle of 111.76(10)°. In contrast, as a result of the dimethylamino groups modest directing ability, with coordination less important, the sodium atom in **4** engages the π-system of the arene ring, in turn increasing the C(13)–Zn(1)–C(23) bond angle to 123.30(11)°.

**Figure 4:**
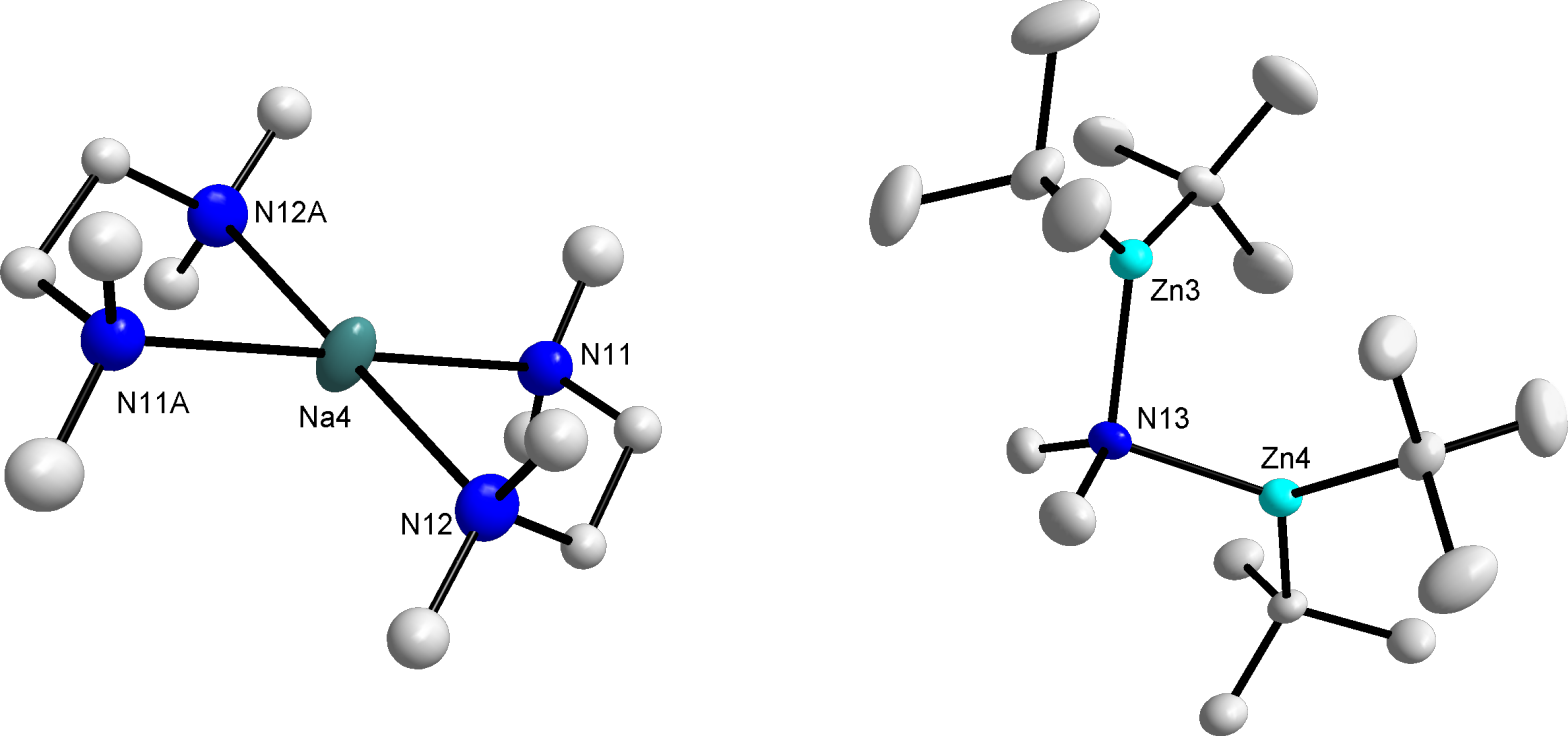
Solvent-separated ion-pair structure of **5** with selective atom labelling and thermal ellipsoids drawn at the 50% probability level. Hydrogen atoms and disordered component of TMEDA are omitted for clarity. Only one of the crystallographically independent cations is displayed, the parameters for the other are the same within experimental error. Symmetry operations to generate equivalent atoms denoted A: 1−x, 1−y, −z. Selected bond distances (Å) and angles (°): Zn(3)–N(13) 2.059(2), Zn(4)–N(13) 2.062(2), Zn(3)–N(13)–Zn(4) 103.67(9), Na(4)–N(11) 2.516(2), Na(4)–N(12) 2.494(3), N(11)–Na(4)–N(12) 75.21(8), N(11)–Na(4)–N(11A) 180.0, N(11)–Na(3)–N(12A) 104.79(8), N(12)–Na(3)–N(12A) 180.0.

The solvent-separated ion-pair co-product **5** ({(TMEDA)_2_Na}^+^{(*t*-Bu_2_Zn)_2_(µ-NMe_2_)}^−^) (Figure 4) adopts a markedly different structural motif from that of **4**. The cation comprises a nearly square planar sodium centre coordinated by two TMEDA molecules (near square planar alkali-metals are rare) [38], whereas the anion consists of two di-*tert*-butylzinc units bridged by a dimethylamino group. The unexpected manifestation of this bridging amide is almost certainly a consequence of utilising the robust homometallic reagent *n*-BuNa at fairly ambient temperatures, with decomposition of either *N*,*N*-dimethylaniline or with precedent in the literature, TMEDA potentially leading to the presence of this cleaved amide fragment [39]. The zinc-nitrogen bond distances in **5** (Zn(3)–N(13) 2.059(2) and Zn(4)–N(13) 2.062(2) Å) are the same within experimental error to each other and in good agreement with related distances in the zinc bisamide ({(PhCH_2_)_2_N}Zn)_2_·C_6_H_6_ (2.0083(13) and 2.0620(12) Å) [40] and in dimeric methyl(diphenylamido)zinc (average value of 2.072(8) Å for the four distinct Zn–N bond lengths) [41]. The best comparison for **5** is possibly provided by the dinuclear zinc guanidinate complex ({Zn(OAr)}_2_(µ-{Me_2_NC(N*i*-Pr)_2_})(µ-NMe_2_)) prepared by Coles [42]. In both complexes, the zinc atoms adopt a distorted trigonal planar geometry (sum of bond angles at Zn(3) and Zn(4) in **5** equal to 358.31° and 358.28°, respectively) but with each zinc atom bound to a bulky aryloxide and guanidinate ligand, the Zn–(µ-amidoN)–Zn bond angle is compressed to 97.34°, in contrast to 103.67(9)° in the more open structure of **5**. A possible consequence of this increased steric hindrance in the guanidinate complex is the shorter zinc–nitrogen bond distances (1.990(3) and 1.982(3) Å) in comparison to those in **5**.

The ^1^H NMR spectrum in deuterated benzene solution of the reaction of **3** and *t*-Bu_2_Zn, which produced crystals of **4** and **5**, revealed the presence of a third distinct species. Four multiplets at 8.03, 7.17, 7.06, and 6.72 ppm are observed for the major *ortho*-metallated product but additionally, shadowing these signals, another different *ortho*-metallated product is evident from four multiplets at 7.87, 7.01, 6.86, and 6.60 ppm. Potentially, this additional unidentified product could be the related dialkyl(aryl) sodium zincate ((TMEDA)Na(*o*-C_6_H_4_-NMe_2_)*t*-BuZn*t*-Bu), reminiscent of the lithium congeners ((THF)_3_Li(*o*-C_6_H_4_-OMe)*t*-BuZn*t*-Bu) and ((PMDETA)Li(*o*-C_6_H_4_-OMe)*t*-BuZn*t*-Bu) prepared by co-complexation reactions of lithiated anisole with *t*-Bu_2_Zn and the relevant Lewis base [27]. The metallation regioselectivity exhibited in the crystalline material is confirmed by treatment of the in situ reaction mixture with iodine. Electrophilic quenching of the two different *ortho*-deprotonated organometallic compounds is depicted in the ^1^H NMR of the crude residue, with a 20.2:1.0 mixture of the *ortho*- and *para*-isomers of iodo-*N*,*N*-dimethylaniline obtained and significantly, a negligible quantity of the *meta*-iodinated product present.

According to theoretical studies, compound **4** and its related bisalkyl(aryl) derivative could be putative intermediates in the first step of the metallation of *N*,*N*-dimethylaniline by TMP-zincates. Thus, the reactivity of this in situ mixture towards the amine TMP(H) was studied, purposely for the formation of (TMEDA)Na(TMP)(C_6_H_4_-NMe_2_)Zn(*t*-Bu) products. First the reactivity towards 1 molar equivalent of TMP(H) at room temperature was investigated and within 30 minutes a white solid precipitated which could be recrystallised from hexane to afford a small amount of translucent crystals.

**Figure 5:**
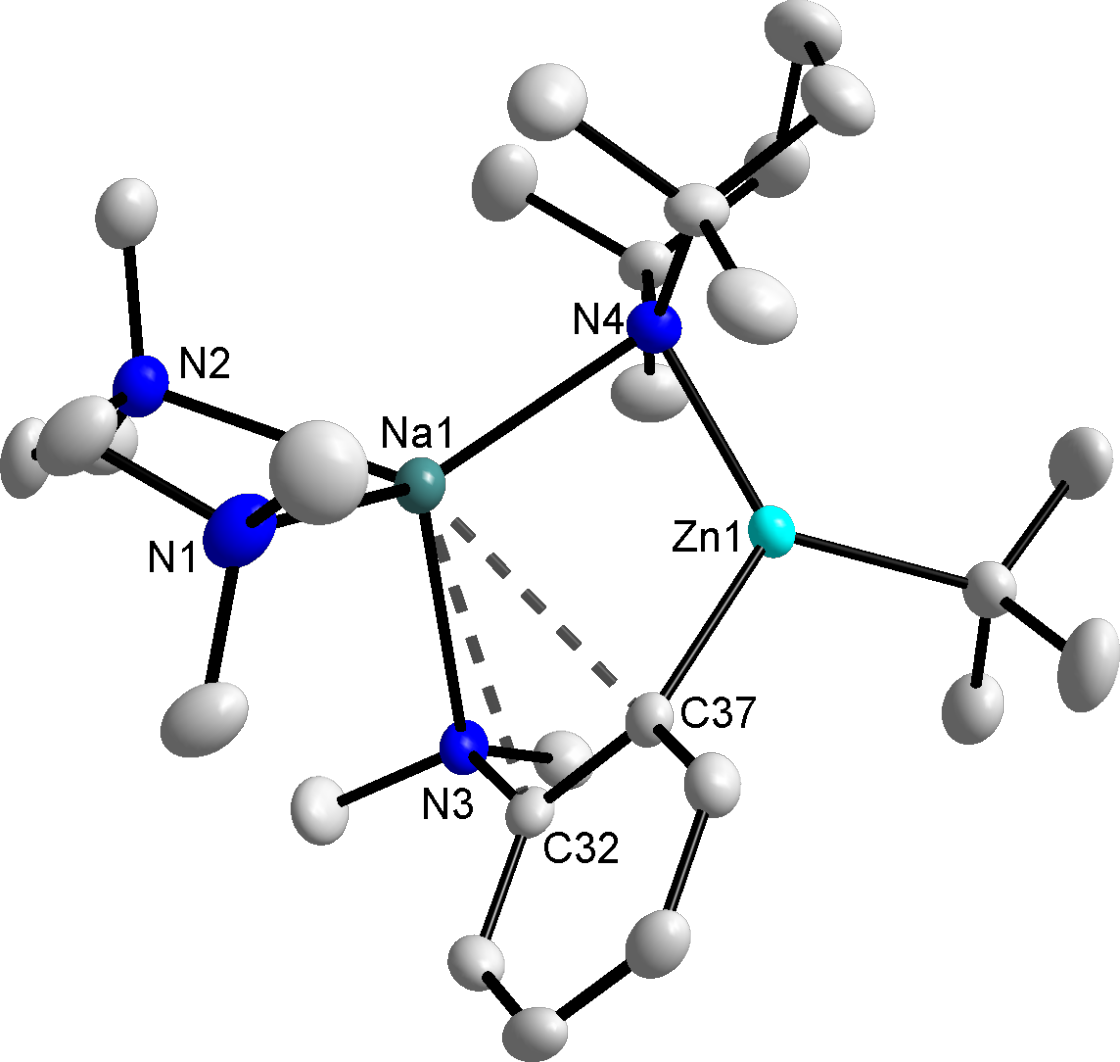
Molecular structure of **6** with selective atom labelling and thermal ellipsoids drawn at the 50% probability level. Hydrogen atoms are omitted for clarity. Secondary contacts between sodium and the anilide rings are denoted by dashed lines. Selected bond distances (Å) and angles (°): Na(1)–N(1) 2.586(2), Na(1)–N(2) 2.568(2), Na(1)–N(3) 2.6991(19), Na(1)–N(4) 2.469(2), Na(1)···C(32) 2.917(2), Na(1)···C(37) 2.768(2), Zn(1)–C(37) 2.079(2), Zn(1)–N(4) 2.0279(17), N(1)–Na(1)–N(4) 131.05(7), N(2)–Na(1)–N(4) 122.82(7), N(1)–Na(1)–N(2) 72.56(6), N(3)–Na(1)–N(4) 105.70(6), N(2)–Na(1)–N(3) 109.31(6), N(1)–Na(1)–N(3) 111.60(7), N(4)-Zn(1)–C37) 114.27(8).

An X-ray crystallographic study showed the molecular structure of these crystals to be **6** ((TMEDA)Na(TMP)(*o*-C_6_H_4_-NMe_2_)Zn(*t*-Bu)) (Figure 5). Revealing a contact ion-pair structure, **6** comprises of the Na–TMP–Zn structural backbone of bimetallic base **1** where TMEDA chelates the sodium atom and zinc is bound to a terminal *tert*-butyl group. In keeping with previous illustrations of the AMM*Zn* reaction, zinc has filled the position vacated by the departing hydrogen atom and significantly in **6**, zinc resides in the *ortho*-position of the aromatic ring. Comparing the regioisomers **2** and **6**, a strong Zn–C σ-bond is formed between the metal and deprotonated carbon atom in both compounds (2.035(4) and 2.079(2) Å in **2** and **6**, respectively), as a result the main difference between the structures arises from the manner in which the metallated arene interacts with the alkali metal. In **2**, with the NMe_2_ unit detached from sodium, the alkali metal interacts more with the π-system of the aromatic ring, principally with the deprotonated *meta*-carbon (2.691(4) Å) generating a four-membered (NaNZnC) ring. In contrast, for **6** the close proximity of the NMe_2_ group enables it to bind with the sodium centre giving a slightly longer than average Na(1)–N(3) bond distance of 2.6991(19) Å (compared to a Na(1)–N(1) bond distance of 2.663(3) Å in **4** and Na–N bond distances in the range 2.448(4)–2.621(3) Å for a series of mononuclear zinc compounds) [36], which closes a larger six-membered highly puckered (NaNZnCCN) central ring. Nevertheless, sodium still π-engages with the aromatic ring (transannular distances of 2.917(2) and 2.768(2) Å) in the characteristic fashion of several AMM*Zn* products.

The relatively simple ^1^H NMR spectrum of the white precipitate in deuterated THF solution revealed the solid to be exclusively product **6**, whereby *N*,*N*-dimethylaniline has been *ortho*-metallated with only four resonances, two doublets (7.48 and 6.81 ppm) and two triplets (6.92 and 6.75 ppm) observed in the aromatic region. Furthermore, the analysis of the aliphatic region of both the ^1^H and ^13^C{^1^H} NMR spectra showed resonances for both *tert*-butyl and TMEDA but notably, resonances which can be attributed to coordinated TMP at 1.74, 1.37 and 1.20 ppm were observed (note that ^1^H NMR resonances of TMP(H) in *d*_8_-THF appear at 1.63, 1.29 and 1.06 ppm).

**Figure 6:**
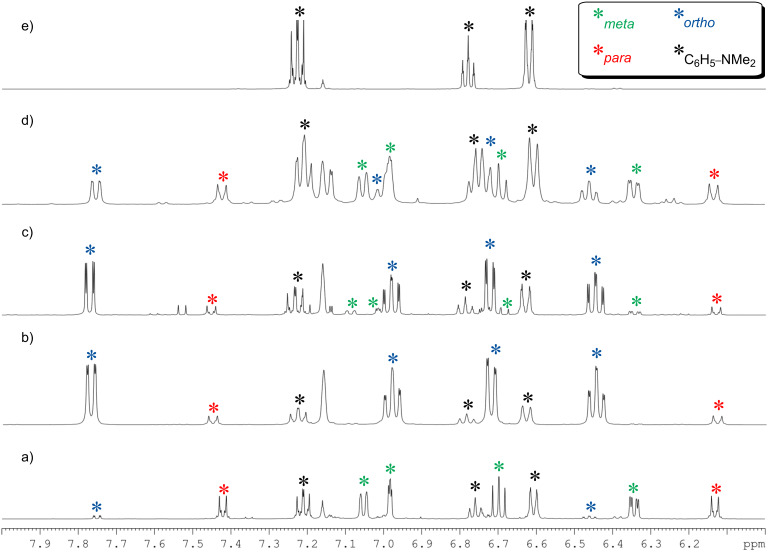
Aromatic region of ^1^H NMR spectra for deuterated benzene solutions of (a) the crude mixture obtained from the reaction of **1** ((TMEDA)Na(TMP)(*t*-Bu)Zn(*t*-Bu)) with 1 equivalent of *N*,*N*-dimethylaniline at room temperature following iodine quenching; (b) the crude mixture obtained from the reaction of BuNa·TMEDA, *N*,*N*-dimethylaniline and *t*-Bu_2_Zn at room temperature following iodine quenching; (c) the crude mixture obtained from the reaction of BuNa·TMEDA, *N*,*N*-dimethylaniline, *t*-Bu_2_Zn and TMP(H) at room temperature following iodine quenching; (d) the crude mixture obtained from the reaction of BuNa·TMEDA, *N*,*N*-dimethylaniline, *t*-Bu_2_Zn and TMP(H) following an overnight reflux and iodine quenching; (e) a standard of *N*,*N*-dimethylaniline.

Regenerating the TMP bridge between sodium and zinc can be considered a copy of the second step of Uchiyama and Nobuto’s theoretically proposed stepwise mechanism. However, should the AMM*Zn* reaction proceed by the two-step procedure, then the *meta*-isomer might emerge as the predominant product despite the isolation of **6**. To establish the complete constitution of an in situ reaction mixture, it was treated with iodine, the crude ^1^H NMR spectrum (Figure 6) revealing that while much of the *ortho*- and *para*-substitution pattern is retained, a small portion of these zincated aryl molecules have isomerised to the *meta*-position (overall *ortho*:*meta*:*para* ratio of 16.6:1.6:1.0 from an *ortho*:*meta*:*para* ratio of 20.2:0:1.0 before TMP(H) addition). For the addition of TMP(H) to the isolated, *ortho*-metallated kinetic intermediate ({(TMEDA)_2_Na}^+^{Zn(*o*-C_6_H_4_–CF_3_)(*t*-Bu)_2_}^−^), we previously reported that the secondary amine facilitates rearrangement of some *ortho*-zincated aryl molecules to *meta*- and *para*-isomers (in a 4.3:2.6:1 ratio, resulting from the amination reaction of TMP(H) with one *t*-Bu group) [28]. The smaller degree of isomerisation in the *N*,*N*-dimethylaniline system can be rationalised at least in part by considering the electronic nature of the different directing groups. With respect to NMe_2_, CF_3_ exerts an increased inductive effect thus weakening the Zn–C*_ortho_* bond to a greater extent, potentially leading to an easier cleavage and thus isomerisation. However, stimulated by this result, the TMP induced isomerisation was pursued with the aim of transforming more *ortho*-material to *meta*. In order to meet this aspiration, the reaction mixture was refluxed overnight, aiding the solubility of **6** and thus presenting greater opportunity for regioisomerisation.

Following the addition of TMP(H) and an 18 hour reflux, the yellow solution was treated with iodine. ^1^H NMR spectroscopic analysis revealed that the make-up of the solution had dramatically changed, with each of the three regioisomers for the mono-iodination of *N*,*N*-dimethylaniline present. Notably, the predominant isomer of iodo-*N*,*N*-dimethylaniline is now the *meta*-substituted product denoted by three multiplets at 7.05, 6.70, and 6.34 and a singlet at 6.98 ppm (Figure 6). The *ortho*-product is still well represented denoted by four multiplets at 7.75, 7.02, 6.74, and 6.46 ppm, while two doublets at 7.42 and 6.13 ppm denote the minor *para*-product with the three regioisomers present in an overall *ortho*:*meta*:*para* 3.7:4.2:1.0 ratio. However, the spectrum also revealed a substantial amount of free *N*,*N*-dimethylaniline (multiplets at 7.22, 6.77, and 6.60 ppm and a singlet at 2.55 ppm for the NMe_2_ group). Simulating the multipart-step of the AMM*Zn* reaction in the aforementioned trifluoromethyl benzene study [28] – by reacting isolated crystals of ({(TMEDA)_2_Na}^+^{Zn(C_6_H_4_-CF_3_)(*t*-Bu)_2_}^−^) with TMP(H) and monitoring by ^1^H NMR spectroscopy – highlighted the significance of this second step in determining not only the final regioselectivity of the metallation but also the reaction yield with the amine found to partially target the metallated aryl anion regenerating sodium TMP-zincate **1** and trifluoromethylbenzene. The existence of such a parallel competitive reaction path could promote the re-formation of *N*,*N*-dimethylaniline and when in the presence of concomitant zincate **1**, be responsible for the increase observed in the *meta*-deprotonated substrate. Further evidence for the regeneration of bimetallic base **1** comes in the identity of two unknown products in the crude mixture, although evidently *N*,*N*-dimethylaniline derivatives, positive identification from NMR analysis was made difficult by the complexity of the aromatic region (two distinct singlets visible in NMe_2_ region at 2.35 and 2.29 ppm). GC-MS (CI mode) analysis verified the presence of the three regioisomers of iodo-*N*,*N*-dimethylaniline but also shed light on the identity of the two unknown species with the MH^+^ peaks at *m*/*z* 373.8 consistent with regioisomers of diiodo-*N*,*N*-dimethylaniline. Thus following the addition of TMP(H), the presence of sodium TMP-zincate **1** in conjunction with the harsher reflux conditions is likely to be the cause for a degree of di-deprotonation of the aromatic molecule.

### DFT calculations

In an attempt to rationalise the different metallation regioselectivities afforded by the homo and heterobimetallic bases, theoretical calculations at the DFT level using the B3LYP method and the 6-311G** basis set were employed to compute the relative stabilities of the four possible regioisomers for the monometallation of *N*,*N*-dimethylaniline.

The relative stabilities of the four model regioisomers (*ortho*-, *meta*-, *para*- and methyl-positions), in which the aniline ring is sodium substituted were calculated and in support of the experimental studies, the *ortho*-isomer is the energetically preferred product (relative energy: 0.00 kcal mol^−1^) with the presence of a Na–N interaction helping its stability. Closely following is the methyl isomer (at 1.61 kcal mol^−1^) but destabilised are the *meta*- and *para*-isomers (by 8.60 and 8.71 kcal mol^−1^, respectively).

On modelling the introduction of TMEDA, the *ortho*-isomer remains the energy minimum structure at 0.00 kcal mol^−1^, however, the energy gap to the other three regioisomers decreases slightly (methyl at 1.35 kcal mol^−1^; *meta* at 6.93 kcal mol^−1^; and *para* at 7.15 kcal mol^−1^). Inspecting the dimensions of the *ortho*-product, TMEDA chelation of the Na atom results in a longer and thus weaker Na–NMe_2_ secondary interaction (increase from 2.289 to 2.501 Å), while the *meta*- and *para*- isomers benefit from TMEDA’s participation with the energies of TMEDA coordination equal to −20.86 and −20.74 kcal mol^−1^, respectively. Tellingly, although the overall energy of reaction for these isomers is exothermic by −5.81 and −5.59 kcal mol^−1^, by way of comparison the product of *ortho*-deprotonation is significantly more exothermic by −12.74 kcal mol^−1^.

**Figure 7:**
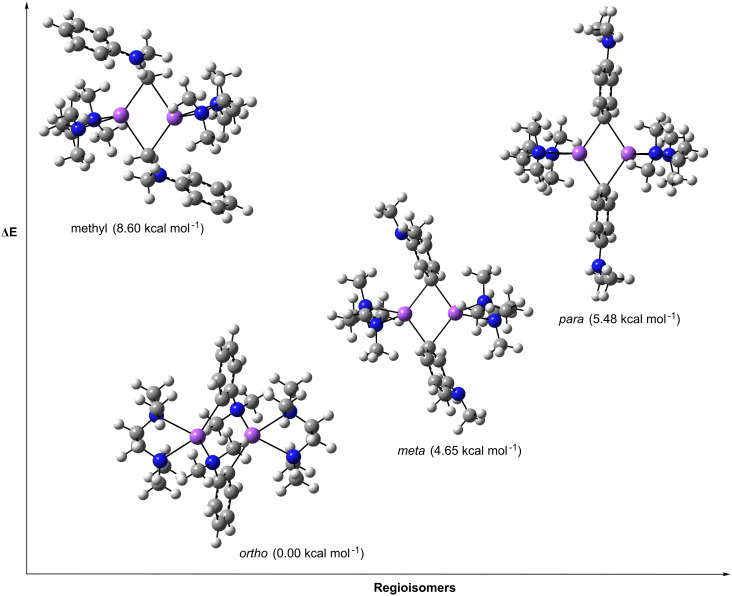
Relative energy sequence of the four theoretical regioisomers of the experimentally observed product **3**.

With respect to dimerisation the most disfavoured is the methyl isomer (relative energy of 8.60 kcal mol^−1^) but in contrast the *meta* (at 4.65 kcal mol^−1^) and *para* (at 5.48 kcal mol^−1^) regioisomers benefit in relation to the *ortho*-product (Figure 7). The −22.08 kcal mol^−1^ energy of dimerisation gained by the *ortho*-isomer is relatively low (compared to −31.29 kcal mol^−1^ for the *meta*-isomer) due to a combination of increased steric repulsions and loss of stabilising interactions. On dimerisation, the Na–N interaction in the *ortho*-product increases in length from 2.501 to 2.662 Å with the Na atom now involved in bonding with two *ortho*-deprotonated C atoms, while there is also a noticeable increase in the Na–N(TMEDA) bond lengths from 2.521 and 2.533Å to 2.604 to 2.674 Å. Through these modelling studies, *ortho*-deprotonation and dimerisation is found to be the energetically preferred metallation pathway by the homometallic reagent, the overall reaction found to be exothermic by −47.55 kcal mol^−1^. Incidentally, on addition of *t*-Bu_2_Zn to each of the three monomeric sodiated regioisomers there is very little difference in the energies of the three products (*ortho* at 0.00 kcal mol^−1^; *meta* at 0.32 kcal mol^−1^; and *para* at 0.41 kcal mol^−1^).

Moving to calculations on the sodium TMP-zincate **1**, concurring with the experimental findings and in sharp contrast to the product obtained via monometallic *n*-BuNa/TMEDA, the *meta*-isomer **2A** is found to be the minimum energy structure (relative energy: 0.00 kcal mol^−1^) but is closely followed by the *para*-isomer **7** (at 0.66 kcal mol^−1^). Notably, the *ortho*-isomer **6A** is destabilised further (at 4.32 kcal mol^−1^); and the least stable of all is metallation at the methyl group (at 9.01 kcal mol^−1^). Hence in the synergic twofold metal system, the *meta*- and *para*-regioisomers are the thermodynamic products. Modelling studies reveal all four deprotonations to be exothermic, with the *meta*-isomer most exothermic by −21.46 kcal mol^−1^.

The modest difference in the relative energies for **2A** and **7** could explain the formation of the sizeable proportion of *para*-product observed experimentally. A comparison of the dimensions in these regioisomers, reveal that the Na contacts to the aryl C atoms are systematically shorter in the energetically preferred *meta*-isomer **2A** (bond distances: 2.594, 3.070, 3.212, 3.959, 4.097, 4.403 Å) than in the analogous *para*-product **7** (bond distances: 2.596, 3.178, 3.239, 4.135, 4.185, 4.596 Å), whereas the Zn–C(aryl) bonds are within 0.001 Å of each other (2.084 and 2.083 Å, respectively) (Table 1). In the *ortho*-isomer **6A**, the product found previously to be expected from conventional (non-synergic, single metal) metallation, the Na–C(*ortho*) bond is decidedly longer (2.747 Å) and thus, weaker than the Na–C(*meta*) bond in the favoured *meta*-isomer **2A** as a result of increased steric constraints. Additionally, with the sodium atom bonding to the NMe_2_ group in **6A**, lone pair–lone pair repulsion between the lone pair of the nitrogen and the nearby *ortho*-carbanion may be a factor in the relative instability of **6A**. Consequently, the principal distinction lies in maximising the strength of the Na···Cπ contacts, and although they may be weak individually, collectively they must contribute appreciably to the overall stability of the unexpected *meta*-deprotonated product.

**Table 1:** Selected bond distances calculated for the three regioisomers **2A**, **6A** and **7**.

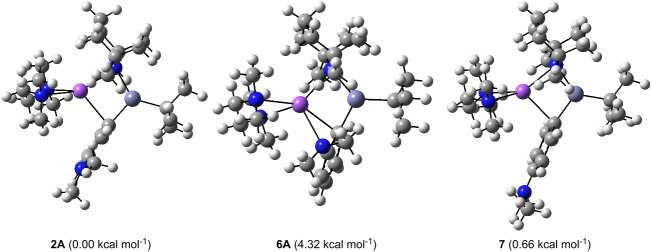			

Bond distance (Å)	2A	6A	7

Zn–C_metallated_	2.084	2.107	2.083
Na–C_metallated_	2.594	2.747	2.596
Na···C_aryl_	3.070, 3.212	2.937	3.178, 3.239

## Conclusion

**Closing remarks on direct AMM*Zn* versus indirect sodiation-zinc co-complexation approaches.** The results of this study highlight the inherent complexity of these metallation reactions even at the metal stage, with a diverse array of organometallic complexes isolated, prior to the recognised intricacies of subsequent electrophilic interception.

Collectively, these results emphasise the synthetic utility of the AMM*Zn* reaction with the employment of a synergic bimetallic base providing entry to previously inaccessible metallation regioselectivities. The AMM*Zn* of *N*,*N*-dimethylaniline by sodium zincate **1** ((TMEDA)Na(TMP)(*t*-Bu)Zn(*t*-Bu)) has been shown to afford a mixture of *ortho*-, *meta*- and *para*-regioisomers in solution, however, the predominant *meta*-substituted derivative **2** ((TMEDA)Na(TMP)(*m*-C_6_H_4_-NMe_2_)Zn(*t*-Bu)) – a regioselectivity normally closed to synthetic aromatic chemistry – can be isolated in reasonable yields in a clean, pure crystalline form.

In an attempt to try and advance our knowledge of the synergic facet of the AMM*Zn*, some control reactions with *N*,*N*-dimethylaniline were performed. With the toothless base *t*-Bu_2_Zn, incapable of directly zincating aromatics, *N*,*N*-dimethylaniline was treated with *n*-BuNa·TMEDA to afford dimeric **3** (((TMEDA)Na(*o*-C_6_H_4_-NMe_2_))_2_) , which in keeping with conventional metallation chemistry exhibits *ortho*-deprotonated anilines. The instability of **3** at ambient temperatures is reflected in its deprotonation of toluene under mild conditions and following co-complexation with the dialkyl zinc, a mixture of two products were isolated from the reaction, namely **4** ((TMEDA)Na(TMP)(*o*-C_6_H_4_-NMe_2_)_2_Zn(*t*-Bu)) and **5** ({(TMEDA)_2_Na}^+^{(*t*-Bu_2_Zn)_2_(µ-NMe_2_)}^−^). The ion-pair product **5** contains a cleaved NMe_2_ group, exemplifying nicely how the reactivity of an alkali-metal can be attenuated when incorporated in a mixed-metal system but to a point where it is still able to play its part in conducting synthetically useful smooth metallations.

The final component of bimetallic base **1**, TMP(H) was introduced at this stage, in what could be considered a copy of the second, multipart-step of the AMM*Zn* process and led to the isolation of **6** ((TMEDA)Na(TMP)(*o*-C_6_H_4_-NMe_2_)Zn(*t*-Bu)), a regioisomer of **2** where the aromatic ring remains *ortho*-zincated. In search of the elusive *meta*-deprotonated derivative **2**, the reaction mixture was refluxed overnight and as a result of the harsher conditions and potentially, regeneration of sodium zincate **1**, the mono-deprotonated *meta*-derivative emerged as the predominant product.

Significantly, these results underline the importance of the acid/base character of TMP(H)/TMP^−^ and the synergic bridge it provides between the two metals, for only when it is present, does the unique *meta*-regioselectivity materialise. Although this product has been engineered from the homometallic components, there are pitfalls in this indirect approach. The low kinetic stability of the *ortho*-sodiated intermediate leads to undesired side reactions such as deprotonation of solvent and decomposition of co-present reactants at temperatures tolerated by the synergic base, and the harsh reflux conditions required to bring about the presence of the *meta*-zincated derivative offers less control, ultimately leading to competing di-metallation of the aromatic substrate.

In addition DFT studies probing the different metallation regioselectivities obtained from homometallic and heterobimetallic bases highlight the importance of the interactions between the alkali metal and the aromatic substrate. With the conventional monometallic reagent *n*-BuNa/TMEDA, metallation is directed to the *ortho*-position by the secondary interaction between Na and the N atom of the NMe_2_ group. In contrast, in the reaction of **1** with *N*,*N*-dimethylaniline, the *meta*-isomer is energetically the most preferred and analysis of the dimensions of the model structures suggest that the strength of the combined Na···Cπ contacts contribute at least in part to its stability. This favourable enthalpic effect, along with the communication between the metals provided by the TMP bridge play a defining role in offering the unique regioselectivity observed in the AMM*Zn* of *N*,*N*-dimethylaniline.

Finally, looking at the bigger picture, this study drives home the fact that bimetallic reagents can offer chemistry distinct to that when two monometallic reagents are added sequentially to the same substrate. The secret to unlocking special synergic reactivity may be found in coupling both metals within the same bimetallic reagent. We plan to capitalise on this advantage in ongoing studies, investigating the generality of the concept.

## Experimental

### General

All reactions were performed under a protective argon atmosphere using standard Schlenk techniques. Hexane, THF and toluene purchased from Sigma-Aldrich, were dried by heating to reflux over sodium benzophenone ketyl and distilled under nitrogen prior to use. *n*-BuNa [43], *t*-Bu_2_Zn [15] and subsequently **1** ((TMEDA)Na(TMP)(*t*-Bu)Zn(*t*-Bu)) [15] were prepared according to literature procedures. The ^1^H NMR spectroscopic experiments were performed on a Bruker DPX400 spectrometer at an operating frequency of 400.13 MHz. The ^13^C NMR spectra were obtained on the same instrument at an operating frequency of 100.62 MHz. Elemental analyses were carried out on a Perkin-Elmer 2400 elemental analyser. Due to the extreme air and moisture sensitivity of **3**, **4/5** and **6**, ideal analyses could not be obtained. GC–MS analysis was performed on an Agilent 7890A apparatus, GC with 5975C triple axes detector, run under CI mode (methane).

### X-ray crystallography

All data were collected at 123(2) K on an Oxford Diffraction Gemini S Diffractometer with Mo Kα radiation (λ = 0.71073 Å). Structures were solved using SHELXS-97 [44] while refinements were carried out on *F*^2^ against all independent reflections by the full-matrix least-squares method using the SHELXL-97 program [44]. With the exception of the carbon atoms of the disordered components of TMEDA present in **4** and **5** all non-hydrogen atoms were refined using anisotropic thermal parameters. CCDC 813771 (**3**), 813772 (**4/5**) and 813773 (**6**) contain the full supplementary crystallographic data for this paper. These data can be obtained free of charge from The Cambridge Crystallographic Data Centre via http://www.ccdc.cam.ac.uk/data_request/cif.

Crystal data for **3**: C_28_H_52_N_6_Na_2_, *M* = 518.74, monoclinic, *P*2_1_/*c*, *a* = 8.8060(9), *b* = 17.5089(12), *c* = 11.1635(11) Å, β = 111.693(12)°, *V* = 1599.3(3) Å^3^, *Z* = 2. 8277 reflections collected, 3797 were unique, *R*_int_ = 0.0454, *R* = 0.0471, *R*_w_ = 0.1133, GOF = 0.905, 169 refined parameters, max and min residual electron density = 0.233 and −0.161 e·Å^−3^.

Crystal data for **4/5**: C_82_H_164_N_13_Na_3_Zn_4_, *M* = 1662.71, triclinic, *P*−1, *a* = 9.3229(2), *b* = 20.0052(4), *c* = 26.2813(6) Å, α = 86.732(2)°, β = 82.721(2)°, γ = 87.247(2)°, *V* = 4850.10(18) Å^3^, *Z* = 2. 57508 reflections collected, 19054 were unique, *R*_int_ = 0.0630, *R* = 0.0413, *R*_w_ = 0.0680, GOF = 0.839, 956 refined parameters, max and min residual electron density = 0.969 and −0.682 e·Å^−3^. Disorder in the TMEDA groups was treated as being over two sites, appropriate restraints on atom-atom distances and temperature factors in these groups were applied.

Crystal data for **6**: C_27_H_53_N_4_NaZn, *M* = 522.09, orthorhombic, *Pbca*, *a* = 16.1371(5), *b* = 17.2078(5), *c* = 21.6306(5) Å, *V* = 6006.5(3) Å^3^, *Z* = 8. 32010 reflections collected, 7240 were unique, *R*_int_ = 0.0540, *R* = 0.0371, *R*_w_ = 0.0853, GOF = 0.881, 311 refined parameters, max and min residual electron density = 0.804 and −0.289 e·Å^−3^.

**Synthesis of 3 (((TMEDA)Na(C_6_H_4_-NMe_2_))_2_) : ***n*-BuNa (2 mmol, 0.16 g) was suspended in hexane (10 mL) and sonicated for 10 min to form a fine dispersion. The Schlenk tube was then cooled to 0 °C in an ice bath before the dropwise introduction of TMEDA (2 mmol, 0.3 mL). *N*,*N*-dimethylaniline (2 mmol, 0.25 mL) was then added dropwise to the clear yellow solution to give an orange solution which was stirred at 0 °C for 2 hours and the resulting light orange precipitate was removed by filtration (0.33 g, 64% – based on monomeric unit). The deep red filtrate solution was concentrated in vacuo and stored in a refrigerator (5 °C) to yield a small amount of X-ray quality, orange crystalline material. ^1^H NMR (400.13 MHz, *d*_12_-cyclohexane, 300 K) δ 7.78 (d, 1H, H*_meta_*), 6.79 (t, 1H, H*_meta′_*), 6.58 (m, 2H, H*_para_* and H*_ortho′_*), 2.83 (s, 6H, N(CH_3_)_2_), 2.22 (s, 4H, CH_2_ (TMEDA)), 2.04 (s, 12H, CH_3_ (TMEDA)); ^13^C NMR (100.62 MHz, *d*_12_-cyclohexane, 300 K) δ 182.3 (Na–C*_ortho_*), 164.1 (C*_ipso_*), 142.7 (C*_meta_*), 124.6 (C*_meta′_*), 120.2 (C*_para_*), 111.5 (C*_ortho′_*), 58.6 (CH_2_ (TMEDA)), 46.0 (CH_3_ (TMEDA)), 45.0 (N(CH_3_)_2_).

**Synthesis of 4 ((TMEDA)Na(TMP)(C_6_H_4_-NMe_2_)_2_Zn(*t*-Bu)) and 5 ({(TMEDA)_2_Na}^+^{(*t*-Bu_2_Zn)_2_(µ-NMe_2_)}^−^):** A hexane solution of *t*-Bu_2_Zn (2 mmol, 0.36 g) was added via cannula to a suspension of **3**, prepared as described above. The mixture was allowed to stir for 1 hour at 0 °C, and then slowly warmed to room temperature and stirred for a further two hours at this temperature. The suspension was gently heated for a couple of minutes to obtain (as close as possible) a yellow homogeneous solution: The latter was left in a Dewar flask filled with hot water overnight to afford 0.6 g of colourless crystals. ^1^H NMR (400.13 MHz, *d*_6_-benzene, 300 K) δ 8.03 (d, 1H, H*_meta_*), 7.87 (d, 0.27H, H*_meta_*), 7.17 (overlap with solvent, 2.5H, H*_meta′_*), 7.06 (t, 1H, H*_para_*), 7.01 (t, 0.29H, H*_para_*), 6.86 (t, 0.29H, H*_meta′_*), 6.72 (d, 1H, H*_ortho′_*), 6.60 (d, 0.28H, H*_ortho′_*); ^13^C NMR (100.62 MHz, *d*_6_-benzene, 300 K) δ 159.4 (Na–C*_ortho_* (major)), 157.0 (C*_ipso_* (major)), 140.2 (C*_meta_* (major)), 139.5 (C*_meta_* (minor)), 126.0 (C*_meta′_* (major)), 125.4 (C*_para_* (minor)), 120.9 (C*_para_* (major)), 119.5 (C*_meta′_* (minor)), 113.8 (C*_ortho′_* (major)), 112.2 (C*_ortho′_* (minor)). The relevant resonances for the remaining quaternary carbons in the minor *ortho*-deprotonated product, C*_ipso_* and Na–C*_aryl_* could not be detected. Due to the presence of three species and resulting complexity, no correlation could be drawn between these signals and those in the aliphatic region.

**Synthesis of 6 ((TMEDA)Na(TMP)(C_6_H_4_-NMe_2_)Zn(*t*-Bu)):** The above-mentioned procedure was repeated and TMP(H) (2 mmol, 0.34 mL) was introduced to the mixture. The resulting yellow suspension was allowed to stir overnight, after which time the resulting white precipitate was collected by filtration (0.32 g, 31%). The precipitate was re-dissolved in warm hexane and allowed to cool to ambient temperature to afford a small amount of colourless crystals (recrystallised yield: 0.07 g, 7% – not optimised). ^1^H NMR (400.13 MHz, *d*_8_-THF, 300 K) δ 7.48 (d, 1H, H*_meta_*), 6.92 (t, 1H, H*_meta′_*), 6.81 (d, 1H, H*_ortho′_*), 6.75 (t, 1H, H*_para_*), 2.70 (s, 6H, N(CH_3_)_2_), 2.31 (s, 4H, CH_2_ (TMEDA)), 2.15 (s, 12H, CH_3_ (TMEDA)), 1.74 (m, 2H, γ-TMP), 1.37 (br, 4H, β-TMP), 1.20 (s, 12H, CH_3_ (TMP)), 0.98 (s, 9H, CH_3_ (*t*-Bu)); ^13^C NMR (100.62 MHz, *d*_8_-THF, 300 K) δ 165.0 (Zn–C*_ortho_*), 160.1 (C*_ipso_*), 141.2 (C*_meta_*), 125.5 (C*_meta′_*), 122.2 (C*_para_*), 114.9 (C*_ortho′_*), 58.9 (CH_2_ (TMEDA)), 53.1 (α-TMP), 46.8 (N(CH_3_)_2_), 46.2 (CH_3_ (TMEDA)), 40.7 (β-TMP), 36.1 (CH_3_ (TMP)), 35.7 (CH_3_ (*t*-Bu)), 20.6 (γ-TMP), 19.8 (C_q_ (*t*-Bu)).

**Electrophilic quenching reactions:** The solutions prepared as described above, were treated with a freshly prepared solution of 1 M iodine in THF (7 mmol, 7 mL) and allowed to stir overnight. A solution of NH_4_Cl (5 mL) was added along with a saturated Na_2_S_2_O_3_ solution until bleaching occurred (5 mL). The organic layer was separated from the aqueous layer and dried over MgSO_4_. After filtration, the solvent was removed under reduced pressure to give a yellow oil. The NMR spectrum of the crude material was recorded to determine the yield of the iodo-product relative to *N*,*N*-dimethylaniline.

**2-Iodo-*N*,*N*-dimethylaniline:** ^1^H NMR (400.13 MHz, *d*_6_-benzene, 300 K) δ 7.76 (d, 1H, H*_meta_*), 6.98 (t, 1H, H*_meta′_*), 6.72 (d, 1H, H*_ortho′_*), 6.46 (t, 1H, H*_para_*), 2.45 (s, 6H, N(CH_3_)_2_); ^13^C NMR (100.62 MHz, *d*_6_-benzene, 300 K) δ 155.4 (C*_ipso_*), 140.5 (C*_meta_*), 129.1 (C*_meta′_*), 125.2 (C*_para_*), 120.9 (C*_ortho′_*), 97.9 (I–C*_ortho_*), 44.7 (N(CH_3_)_2_).

**3-Iodo-*N*,*N*-dimethylaniline:** ^1^H NMR (400.13 MHz, *d*_6_-benzene, 300 K) δ 7.05 (d, 1H, H*_para_*), 6.98 (s, 1H, H*_ortho_*), 6.70 (t, 1H, H*_meta′_*), 6.34 (d, 1H, H*_ortho′_*), 2.32 (s, 6H, N(CH_3_)_2_); ^13^C NMR (100.62 MHz, *d*_6_-benzene, 300 K) δ 151.8 (C*_ipso_*), 130.6 (C*_meta′_*), 125.5 (C*_para_*), 121.4 (C*_ortho_*), 111.7 (C*_ortho′_*), 96.0 (I–C*_meta_*), 39.7 (N(CH_3_)_2_).

**4-Iodo-*N*,*N*-dimethylaniline:**^ 1^H NMR (400.13 MHz, *d*_6_-benzene, 300 K) δ 7.42 (d, 2H, H*_meta_*), 6.13 (d, 2H, H*_ortho_*), 2.36 (s, 6H, N(CH_3_)_2_); ^13^C NMR (100.62 MHz, *d*_6_-benzene, 300 K) δ 150.9 (C*_ipso_*), 137.8 (C*_meta_*), 115.0 (C*_ortho_*), 77.7 (I–C*_para_*), 39.8 (N(CH_3_)_2_).

GC–MS (CI) *m*/*z*: [M + H]^+^ calcd for C_8_H_10_IN, 246.9; found, 247.9.

For unidentified species, GC–MS (CI) *m*/*z*: [M + H]^+^ calcd for C_8_H_9_I_2_N, 372.8; found, 373.8.

## Supporting Information

File 1 Computational details and NMR spectra for compounds **3**, **4**, **5** and **6**.

File 2 CIF files giving crystallographic data for compounds **3**, **4**, **5** and **6**.
