# Supplementary material for: Meta-metallation of N,N-dimethylaniline: Contrasting direct sodium-mediated zincation with indirect sodiation-dialkylzinc co-complexation
Source: Beilstein J Org Chem. 2011 Sep 6;7:1234–48. doi: 10.3762/bjoc.7.144 (PMC3182433; doi:10.3762/bjoc.7.144)
Supplement: File 1 — Computational details and NMR spectra for compounds 3, 4, 5 and 6. [file Beilstein_J_Org_Chem-07-1234-s001.pdf]

Supporting Information

for

Meta-metallation of *N,N*-dimethylaniline: contrasting direct sodium-mediated zincation with indirect sodiation-dialkylzinc co-complexation

David R. Armstrong, Liam Balloch*, Eva Hevia, Alan R. Kennedy, Robert E. Mulvey*, Charles T. O'Hara and Stuart D. Robertson

Address: WestCHEM, Department of Pure and Applied Chemistry, University of Strathclyde, Glasgow, G1 1XL, United Kingdom

Email: David R. Armstrong - d.r.armstrong@strath.ac.uk; Liam Balloch* - liam.balloch@strath.ac.uk; Robert E. Mulvey* - r.e.mulvey@strath.ac.uk

* Corresponding author

Computational details and NMR spectra for compounds 3, 4, 5 and 6

DFT calculations were carried out using the Gaussian G03 computational package [1]. The B3LYP density functionals [2,3] were used along with the 6-311G(d,p) basis set [4,5]. After the geometry optimisation of each molecule, a frequency analysis was carried out. The resulting calculated zero-point energy was added to the electronic energy and this is the energy value quoted below.

_o_-NaC_6H_4-NMe_2

<u>Principal bond lengths (Å) and angles (°)</u>

Na-N	2.289 Å	C-Na-N	63.0°
Na-C	2.439 Å	Na-N-C	85.6°
Na-C_i	2.758 Å	N-C-C	117.8°
C_i-N	1.487 Å	C-C-Na	93.6°

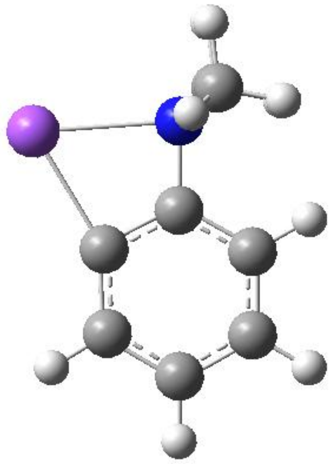

_m_-NaC_6H_4-NMe_2

<u>Principal bond lengths (Å) and angles (°)</u>

Na-C	2.301 Å
Na-C-C	122.8 ° 120.5 °

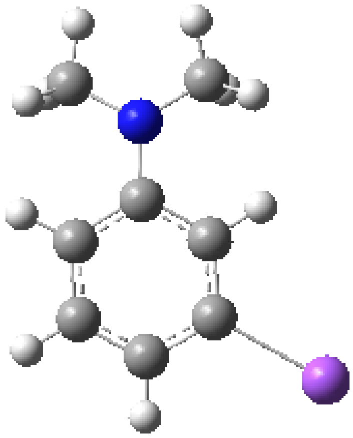

_p_-NaC_6H_4-NMe_2

<u>Principal bond lengths (Å) and angles (°)</u>

Na-C	2.301 Å
Na-C-C	123.0 °

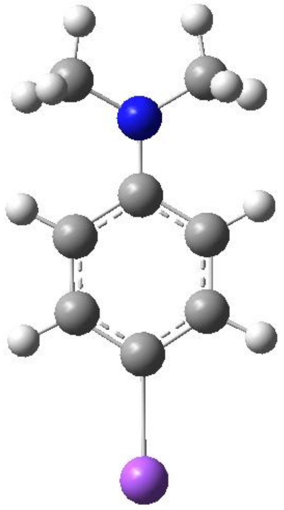

C_6H_5-N(Me)CH_2Na

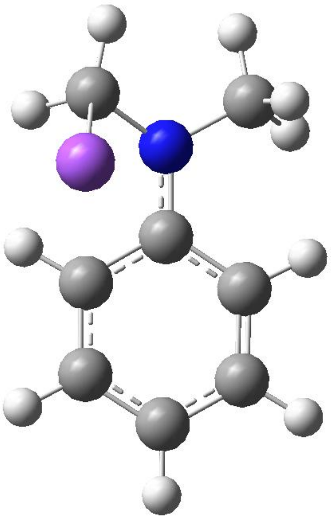

Principal bond lengths (Å) and angles (°)

Na-C	2.387 Å	N-C_i	1.371 Å
Na..C_i	3.023 Å	N-C_{Me}	1.469 Å 1.451Å
Na..C_o	2.862 Å		
Na..N	2.959 Å		

Energies/au

***o*-NaC_6H_4-NMe_2**	-527.821237
***m*-NaC_6H_4-NMe_2**	-527.807529
***p*-NaC_6H_4-NMe_2**	-527.807352
**C_6H_5-N(Me)CH_2Na**	-527.818668

Relative energies/kcal mol^{-1}

ortho:*meta*:*para*:*methyl* 0.00:8.60:8.71:1.61

[*o*-NaC$_6$H$_4$-NMe$_2$(TMEDA)]

<u>Principal bond lengths (Å) and angles (°)</u>

Na-N$_{tm}$	2.521 Å 2.533 Å
Na-N	2.501 Å
Na-C	2.357 Å
C$_i$-N	1.488 Å
N$_{tm}$-Na-N$_{tm}$	74.5 °
C-Na-N	61.1 °
Na-N-C	86.8 °
N-C-C	117.5 °
C-C-Na	94.6 °

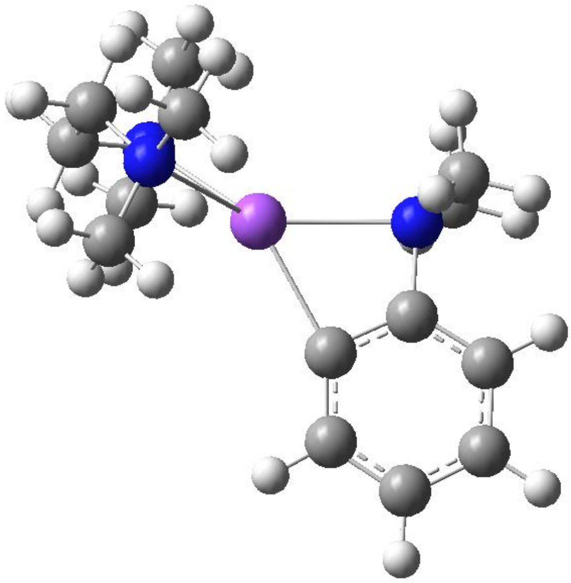

[*m*-NaC$_6$H$_4$-NMe$_2$(TMEDA)]

<u>Principal bond lengths (Å) and angles (°)</u>

Na-C	2.356 Å
Na-N	2.513 Å 2.517 Å
Na-C-C	119.5 ° 125.4 °
N-Na-N	74.7 °

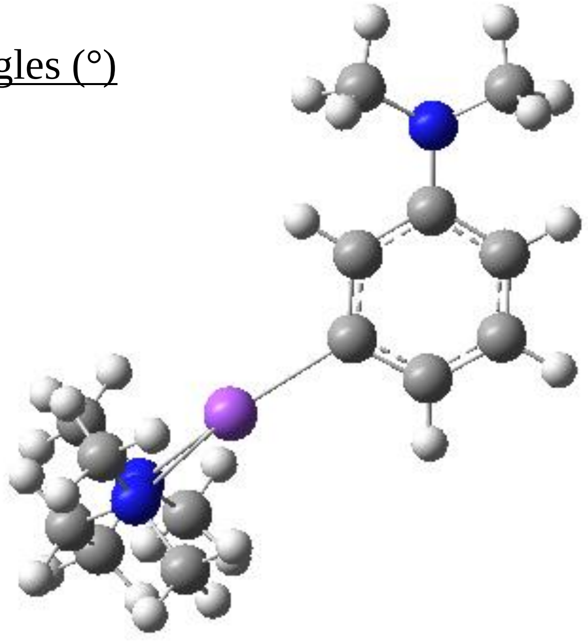

[*p*-NaC_6H_4-NMe_2(TMEDA)]

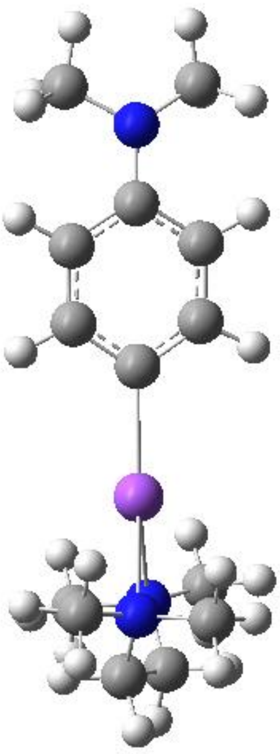

Principal bond lengths (Å) and angles (°)

Na-C	2.351 Å
Na-N	2.518 Å 2.517 Å
Na-C-C	123.8 ° 123.7 °
N-Na-N	74.6 °

[{C_6H_5-N(Me)CH_2Na}(TMEDA)]

Principal bond lengths (Å) and angles (°)

Na-C	2.424 Å
Na..C_i	3.118 Å
Na..C_o	2.963 Å
N-C_{Me}	1.489 Å 1.448 Å
Na-N	2.518 Å 2.527 Å
N-Na-N	74.8 °

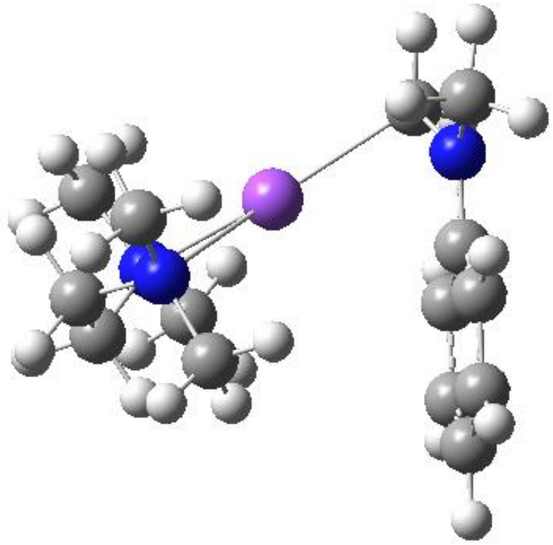

Energies/au

**[*o*-NaC$_6$H$_4$-NMe$_2$(TMEDA)]**	-875.471777
**[*m*-NaC$_6$H$_4$-NMe$_2$(TMEDA)]**	-875.460731
**[*p*-NaC$_6$H$_4$-NMe$_2$(TMEDA)]**	-875.460371
**[{C$_6$H$_5$-N(Me)CH$_2$Na}(TMEDA)]**	-875.469626

Relative energies kcal mol^{-1}

ortho:*meta*:*para*:methyl 0.00:6.93:7.15:1.35

Energies of the reactions

TMEDA·BuNa + C$_6$H$_5$-NMe$_2$ → BuH + TMEDA·NaC$_6$H$_4$-NMe$_2$

**[*o*-NaC$_6$H$_4$-NMe$_2$(TMEDA)]**	ΔE = -12.74 kcal mol^{-1}
**[*m*-NaC$_6$H$_4$-NMe$_2$(TMEDA)]**	ΔE = -5.81 kcal mol^{-1}
**[*p*-NaC$_6$H$_4$-NMe$_2$(TMEDA)]**	ΔE = -5.59 kcal mol^{-1}
**[{C$_6$H$_5$-N(Me)CH$_2$Na}(TMEDA)]**	ΔE = -11.39 kcal mol^{-1}

[*o*-NaC_6H_4-NMe_2(TMEDA)]$_2$

Principal bond lengths (Å) and angles (°)

Na-N_{tm}	2.604 Å 2.674 Å
Na-N	2.662 Å
Na-C	2.594 Å 2.605 Å
C_i-N	1.461 Å
N_{tm}-Na-N_{tm}	70.6 °
C-Na-N	55.6 °
Na-N-C	85.3 °
N-C-C	117.0 °
C-C-Na	88.8 °
C-Na-C	105.3 °
Na-C-Na	74.7 °

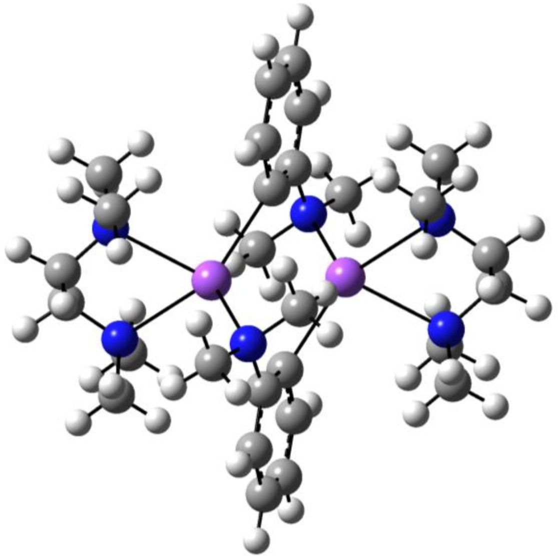

[*m*-NaC_6H_4-NMe_2(TMEDA)]$_2$

Principal bond lengths (Å) and angles (°)

Na-C	2.571 Å 2.532 Å
Na-N	2.546 Å 2.546 Å
Na-C-Na	72.1 °
C-Na-C	107.9 °
N-Na-N	74.7 °

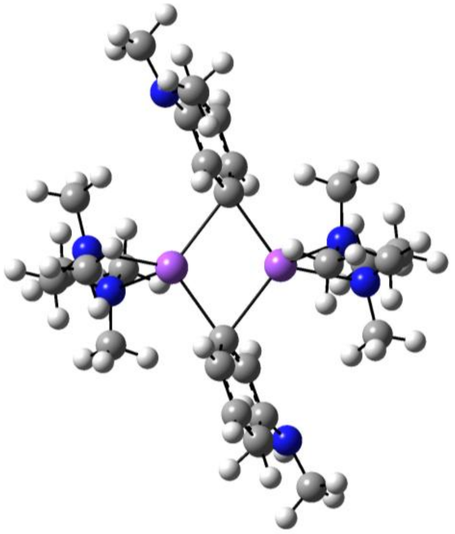

[*p*-NaC$_6$H$_4$-NMe$_2$(TMEDA)]$_2$

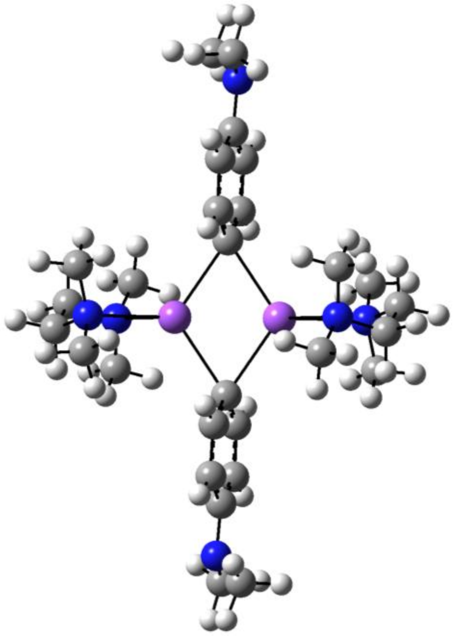

Principal bond lengths (Å) and angles (°)

Na-C	2.551 Å 2.548 Å
Na-N	2.545 Å 2.544 Å
Na-C-Na	69.1 °
C-Na-C	110.9 °
N-Na-N	74.7 °

[{C$_6$H$_5$-N(Me)CH$_2$Na}(TMEDA)]$_2$

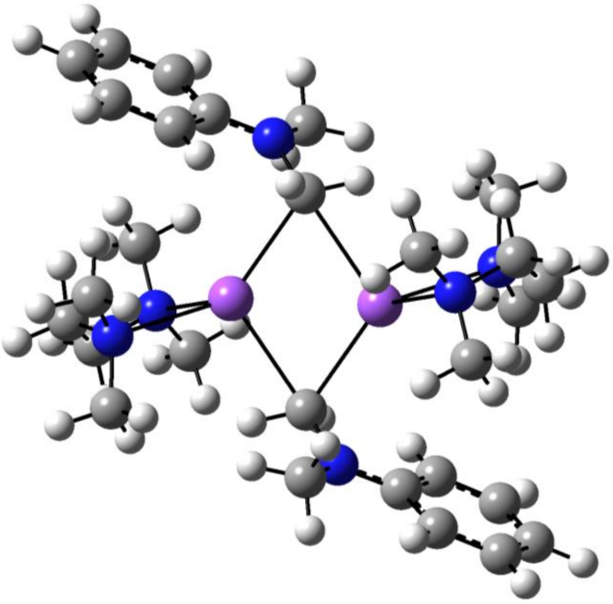

Principal bond lengths (Å) and angles (°)

Na-C	2.598 Å 2.563 Å
Na..C$_i$	3.632 Å
Na..C$_o$	4.320 Å
N-C$_{Me}$	1.496 Å 1.459 Å
Na-N	2.581 Å 2.587 Å
N-Na-N	73.5 °
Na-C-Na	74.1 °
C-Na-C	105.9 °

Energies/au

**[*o*-NaC_6H_4-NMe_2(TMEDA)]$_2$**	-1750.978739
**[*m*-NaC_6H_4-NMe_2(TMEDA)]$_2$**	-1750.971326
**[*p*-NaC_6H_4-NMe_2(TMEDA)]$_2$**	-1750.969999
**[{C_6H_5-N(Me)CH_2Na}(TMEDA)]$_2$**	-1750.965031

Relative energies/kcal mol^{-1}

ortho:*meta*:*para*:methyl 0.00:4.65:5.48:8.60

Energies of the reactions

2 TMEDA·BuNa + 2 C_6H_5-NMe_2 → 2 BuH + [TMEDA·NaC_6H_4-NMe_2]$_2$

**[*o*-NaC_6H_4-NMe_2(TMEDA)]$_2$**	ΔE = -47.55 kcal mol^{-1}
**[*m*-NaC_6H_4-NMe_2(TMEDA)]$_2$**	ΔE = -42.90 kcal mol^{-1}
**[*p*-NaC_6H_4-NMe_2(TMEDA)]$_2$**	ΔE = -42.07 kcal mol^{-1}
**[{C_6H_5-N(Me)CH_2Na}(TMEDA)]$_2$**	ΔE = -38.95 kcal mol^{-1}

[(TMEDA)Na(μ-TMP)(μ-*m*-C_6H_4-NMe_2)Zn(*t*Bu)]

Principal bond lengths (Å)

Na-C	2.594
Na…C	3.070 3.212
Na-N_{TMP}	2.454
Na-N_{TMEDA}	2.602 2.606
Zn-C_{br}	2.084
Zn-C_{ter}	2.040
Zn-N	2.054

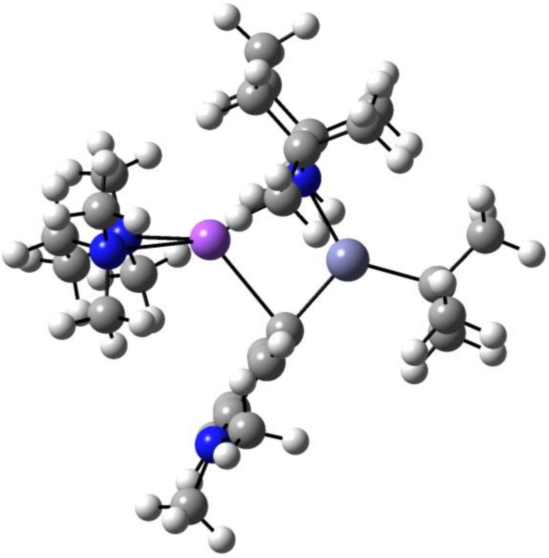

[(TMEDA)Na(μ-TMP)(μ-*p*-C_6H_4-NMe_2)Zn(*t*Bu)]

Principal bond lengths (Å)

Na-C	2.596
Na…C	3.178 3.239
Na-N_{TMP}	2.445
Na-N_{TMEDA}	2.599 2.591
Zn-C_{br}	2.083
Zn-C_{ter}	2.042
Zn-N	2.055

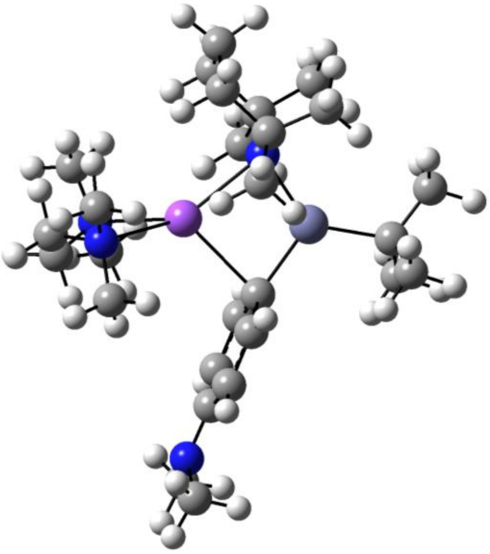

[(TMEDA)Na(μ-TMP)(μ-*o*-C_6H_4-NMe_2)Zn(*t*Bu)]

<u>Principal bond lengths (Å)</u>

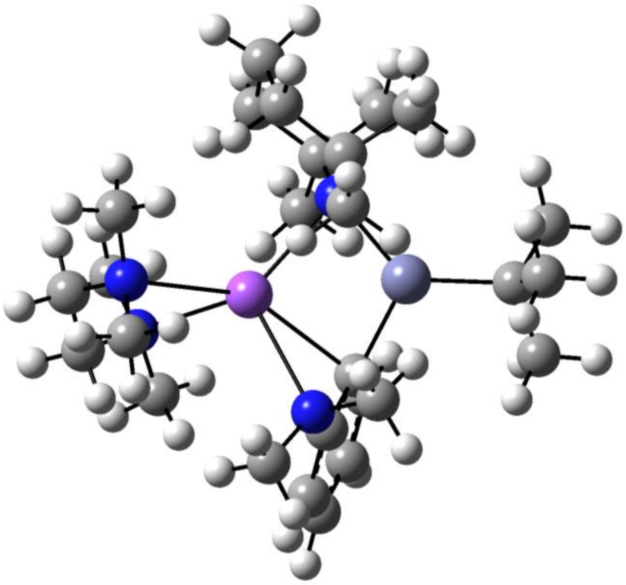

Na-C	2.747
Na…C	2.937
Na-N	2.780
Na-N_{TMP}	2.497
Na-N_{TMEDA}	2.651 2.807
Zn-C_{br}	2.107
Zn-C_{ter}	2.055
Zn-N	2.048

Energies/au

**[(TMEDA)Na(μ-TMP)(μ-*m*-C_6H_4-NMe_2)Zn(*t*Bu)]**	-3221.012984
**[(TMEDA)Na(μ-TMP)(μ-*p*-C_6H_4-NMe_2)Zn(*t*Bu)]**	-3221.011929
**[(TMEDA)Na(μ-TMP)(μ-*o*-C_6H_4-NMe_2)Zn(*t*Bu)]**	-3221.006092
**[(TMEDA)Na(μ-TMP)(μ-C_6H_5-N(CH_2)Me)Zn(*t*Bu)]**	-3220.998630

Relative energies/kcal mol^{-1}

meta:*para*:*ortho*:methyl 0.00:0.66:4.32:9.01

Energies of the reactions

[(TMEDA)Na(μ-TMP)(μ-*t*Bu)Zn(*t*Bu)] + C_6H_5-NMe_2 →

BuH + [(TMEDA)Na(μ-TMP)(μ-C_6H_4-NMe_2)Zn(*t*Bu)]

**[(TMEDA)Na(μ-TMP)(μ-*m*-C_6H_4-NMe_2)Zn(*t*Bu)]**	ΔE =-21.46 kcal mol^{-1}
**[(TMEDA)Na(μ-TMP)(μ-*p*-C_6H_4-NMe_2)Zn(*t*Bu)]**	ΔE=-20.80 kcal mol^{-1}
**[(TMEDA)Na(μ-TMP)(μ-*o*-C_6H_4-NMe_2)Zn(*t*Bu)]**	ΔE=-17.13 kcal mol^{-1}
**[(TMEDA)Na(μ-TMP)(μ-C_6H_5-N(CH_2)Me)Zn(*t*Bu)]**	ΔE=-12.45 kcal mol^{-1}

NMR spectroscopic analysis

➢ Key

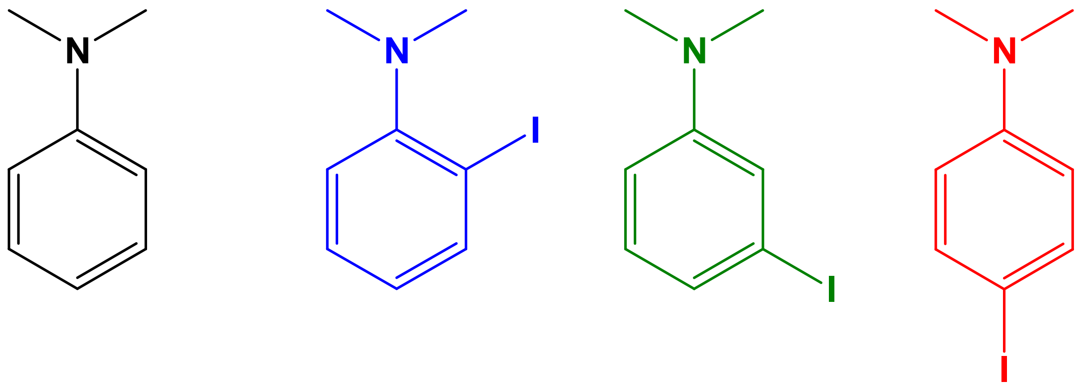

N,N*-dimethylaniline** ***ortho ***meta*** ***para***

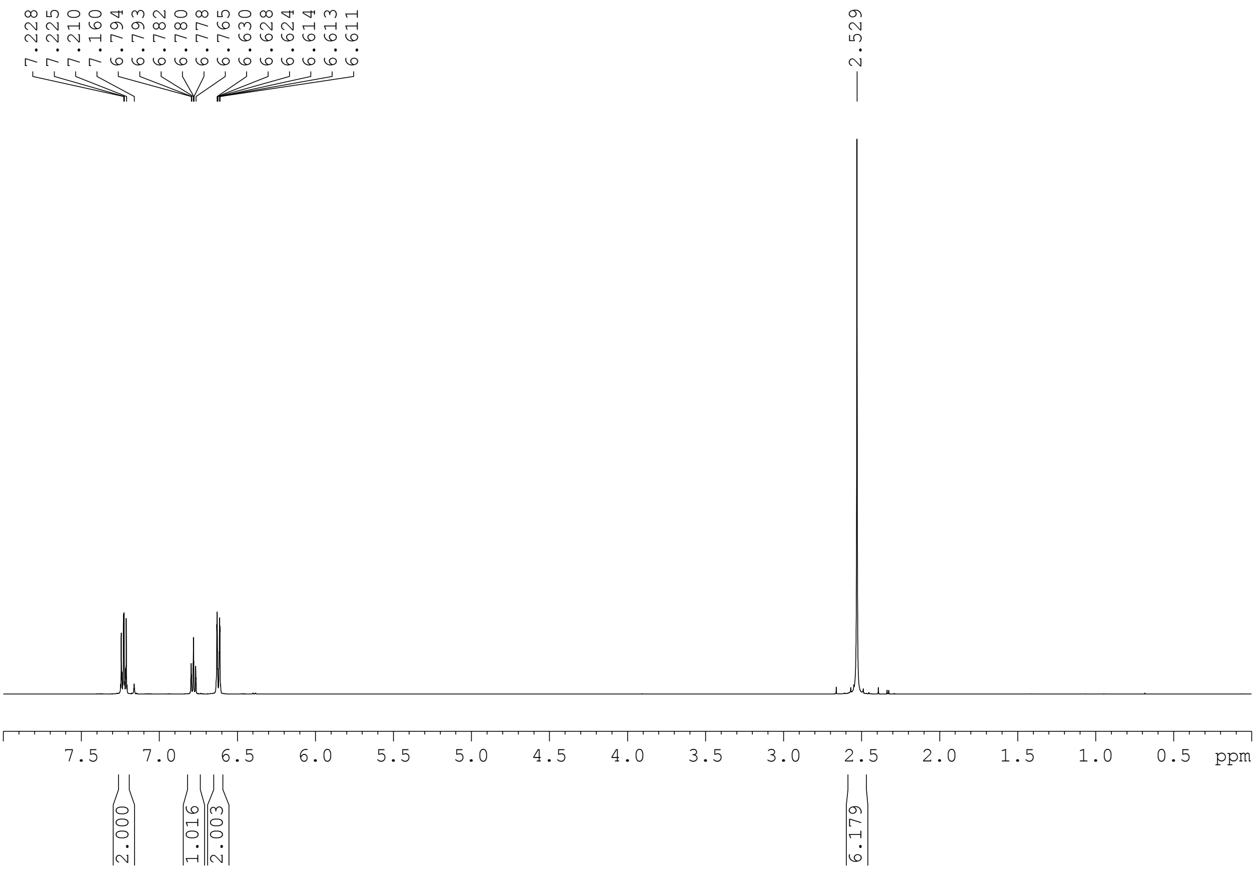

Spectrum 1. ^{1}H NMR (400.13 MHz, 300 K) spectrum of *N,N*-dimethylaniline in C_6D_6 solution.

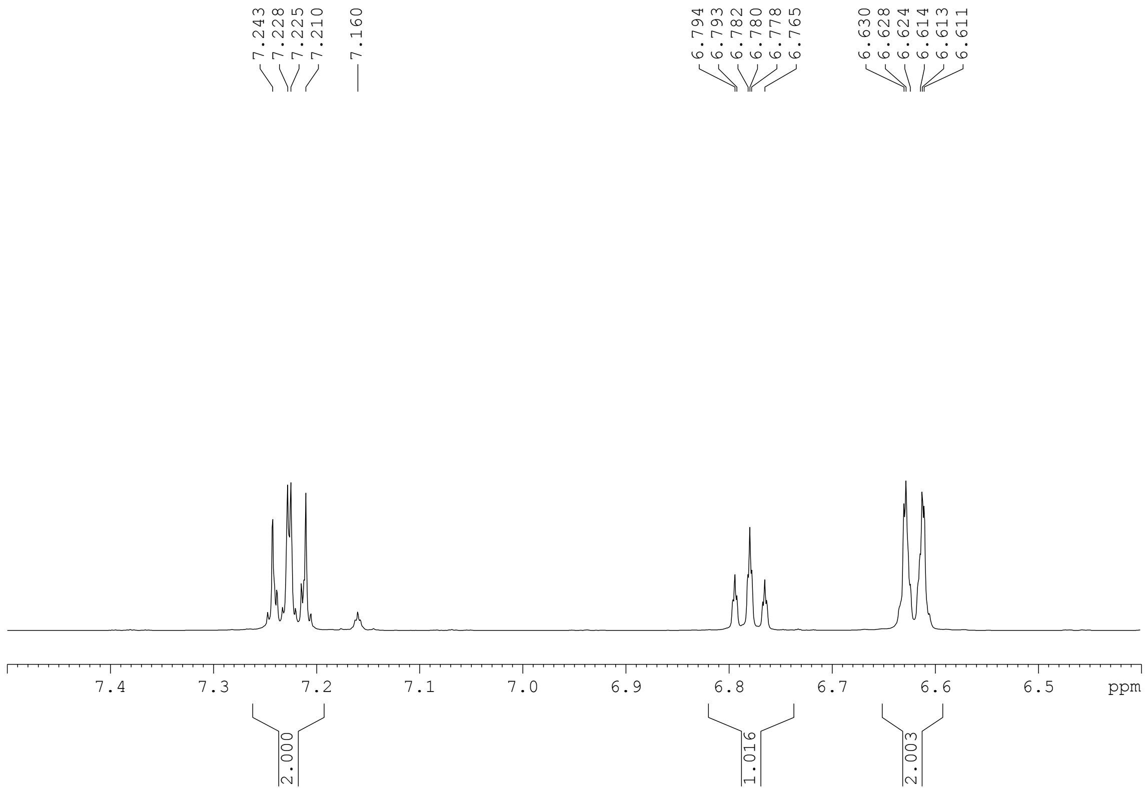

Spectrum 2. Aromatic region of ^{1}H NMR spectrum of *N*,*N*-dimethylaniline in C_6D_6 solution.

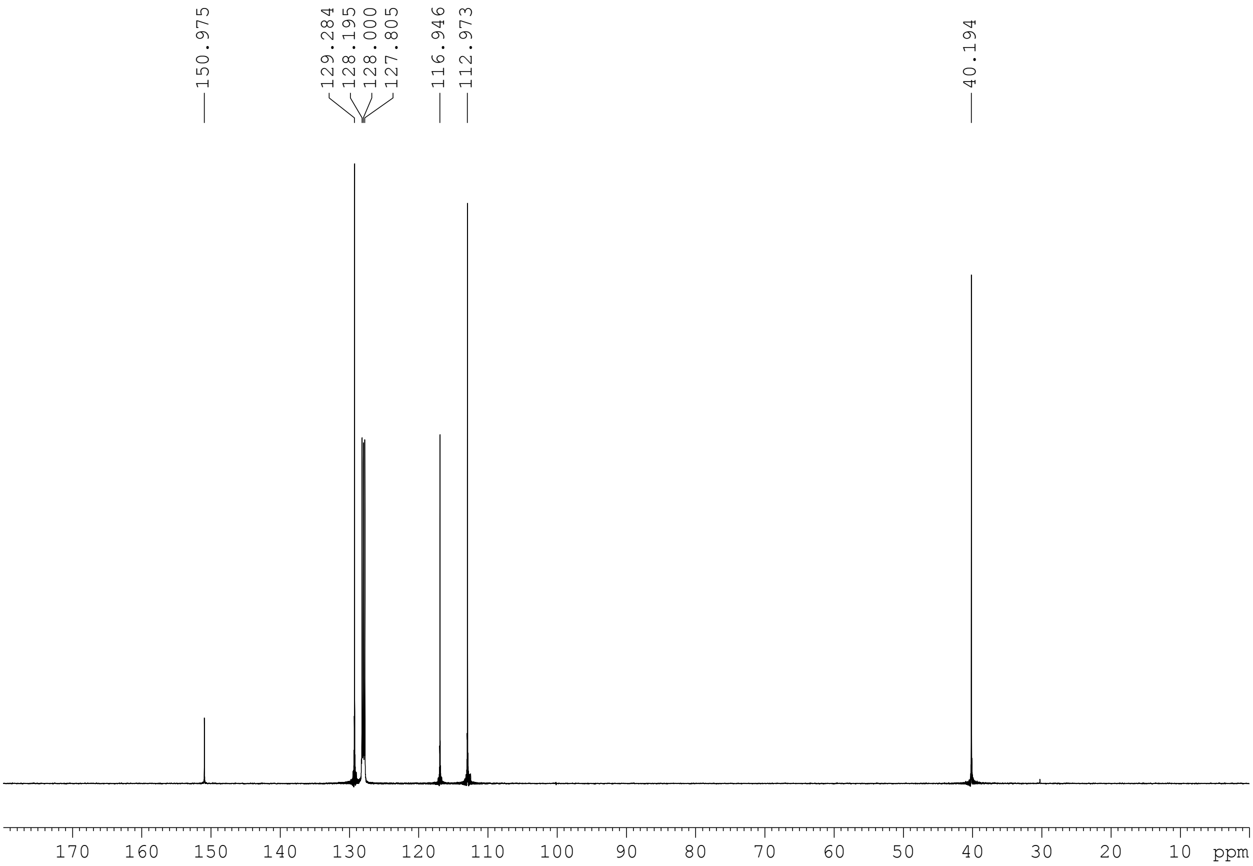

Spectrum 3. ^{13}C {^{1}H} NMR (100.62 MHz, 300 K) spectrum of *N*,*N*-dimethylaniline in C_6D_6 solution.

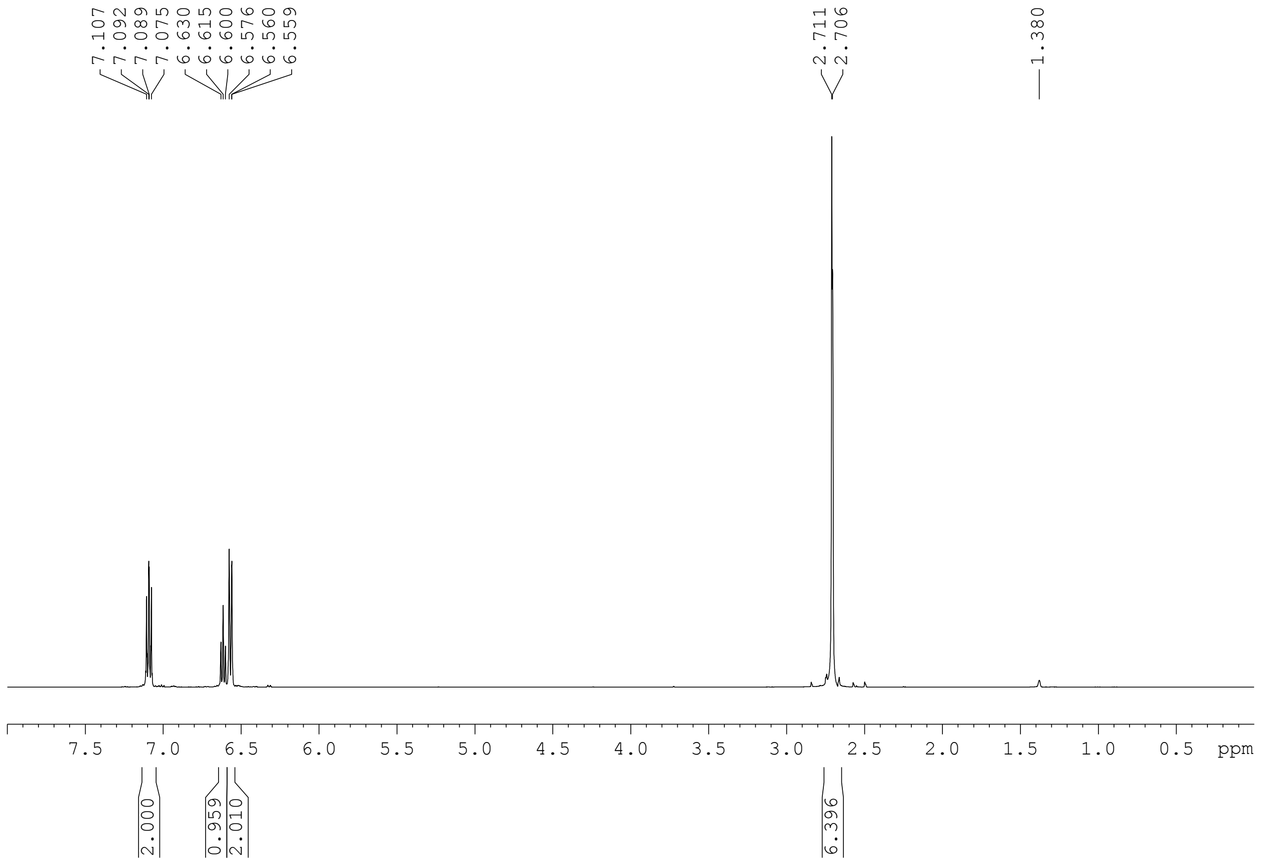

Spectrum 4. ^{1}H NMR (400.13 MHz, 300 K) spectrum of *N*,*N*-dimethylaniline in C_6D_{12} solution.

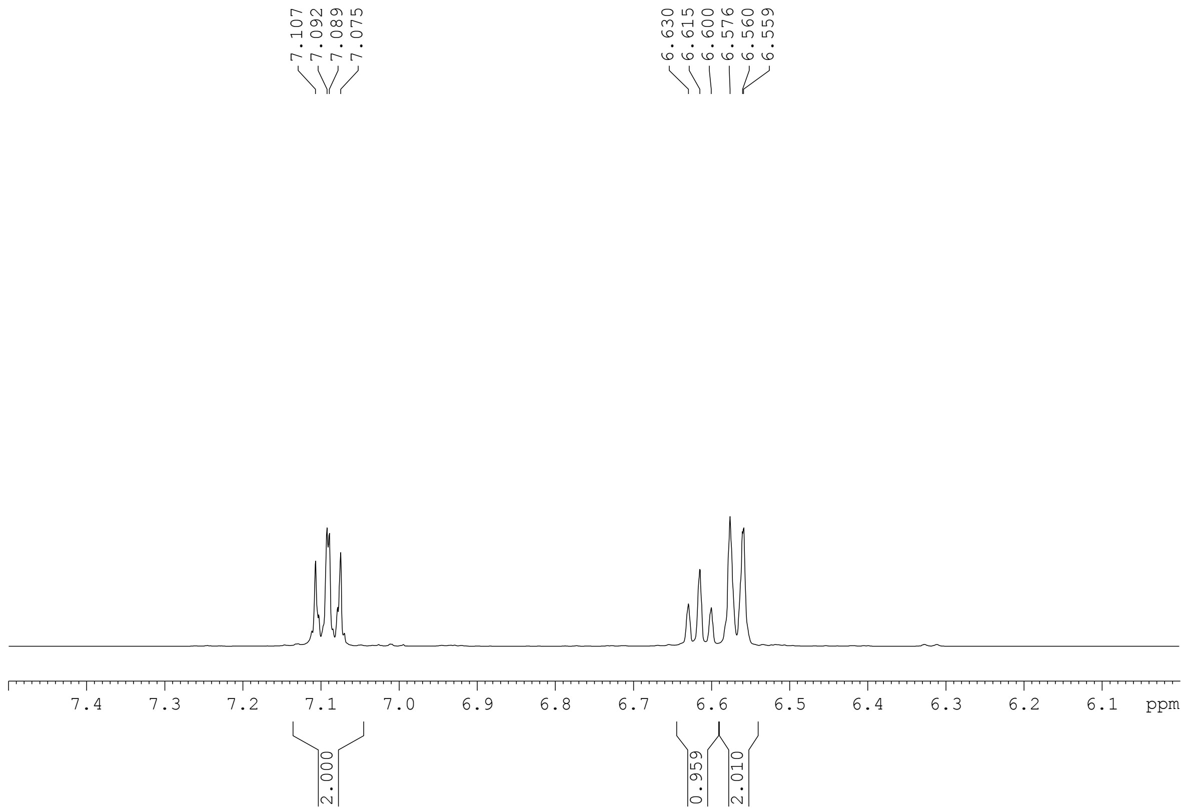

Spectrum 5. Aromatic region of ^{1}H NMR spectrum of *N*,*N*-dimethylaniline in C_6D_{12} solution.

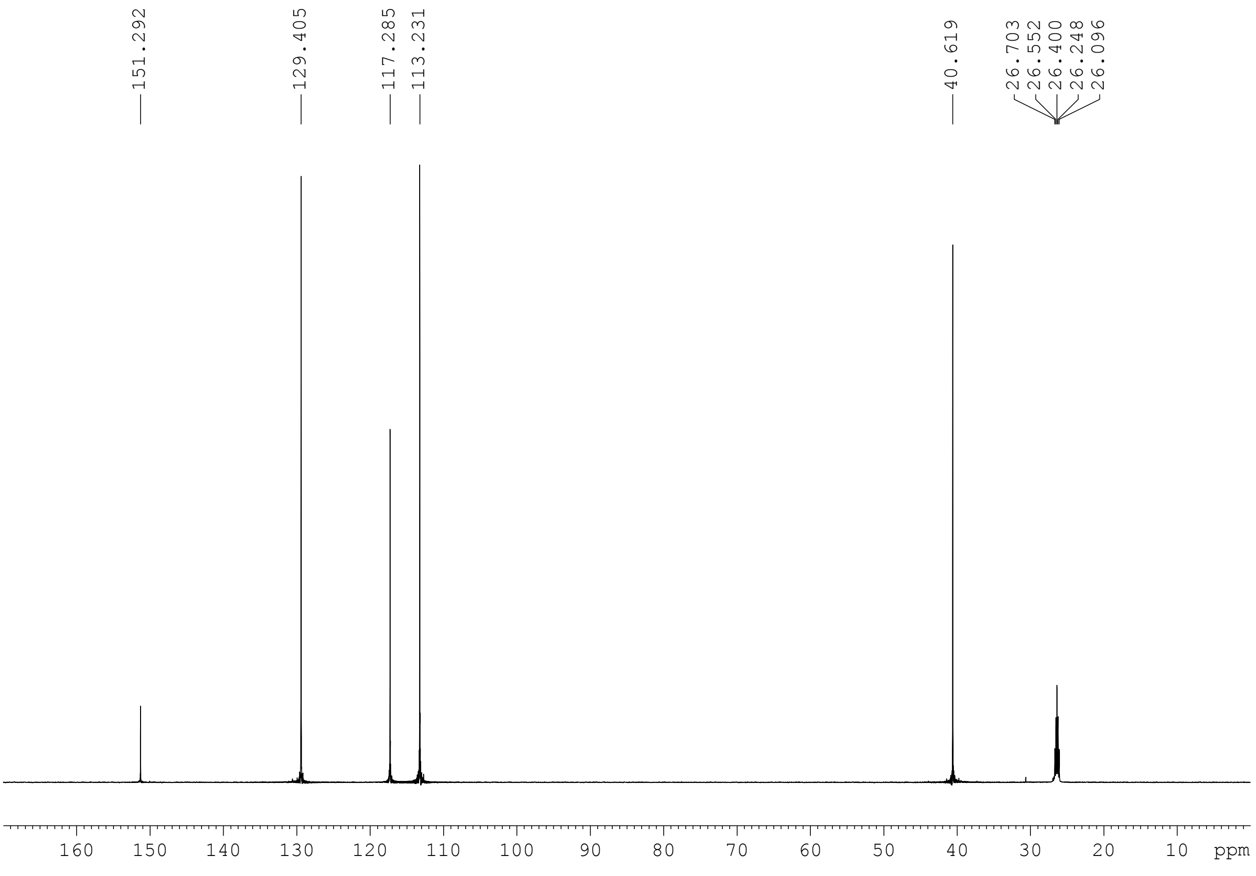

Spectrum 6. ^{13}C {^{1}H} NMR (100.62 MHz, 300 K) spectrum of *N*,*N*-dimethylaniline in C_6D_{12} solution.

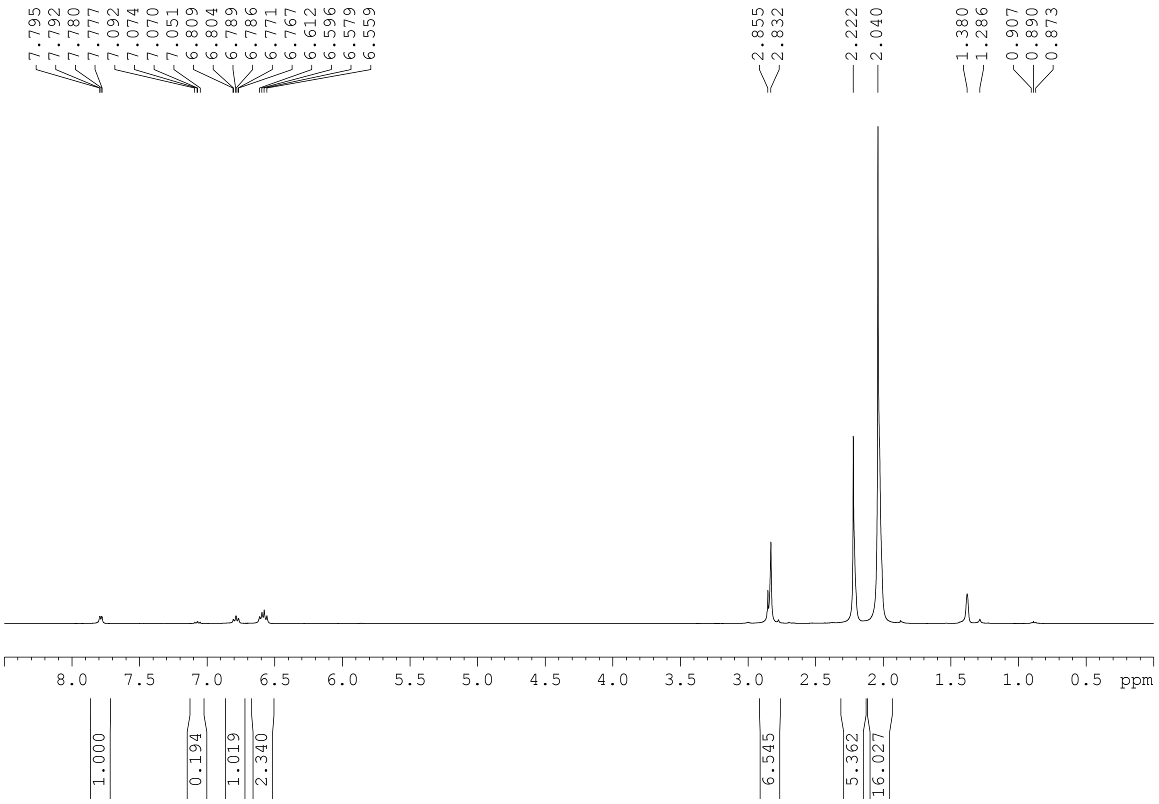

Spectrum 7. ^{1}H NMR (400.13 MHz, 300 K) spectrum of **3** in C_6D_{12} solution.

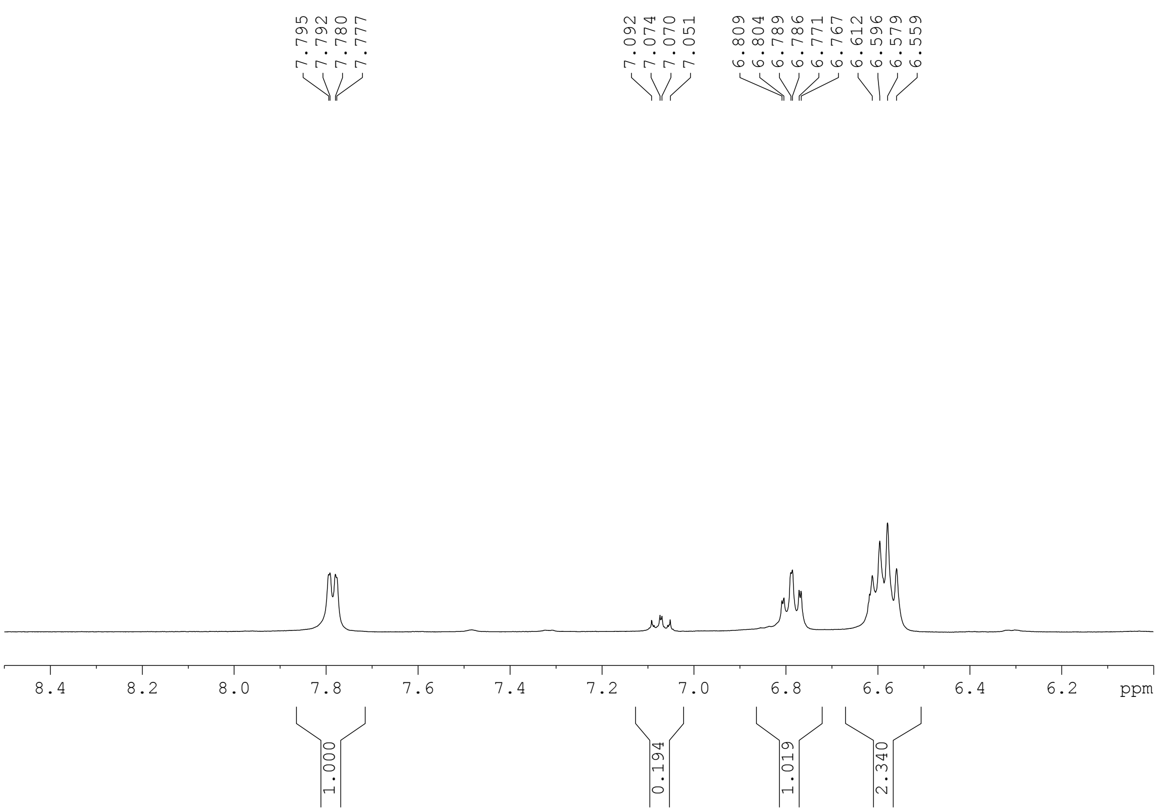

Spectrum 8. Aromatic region of ^{1}H NMR spectrum of **3** in C_6D_{12} solution.

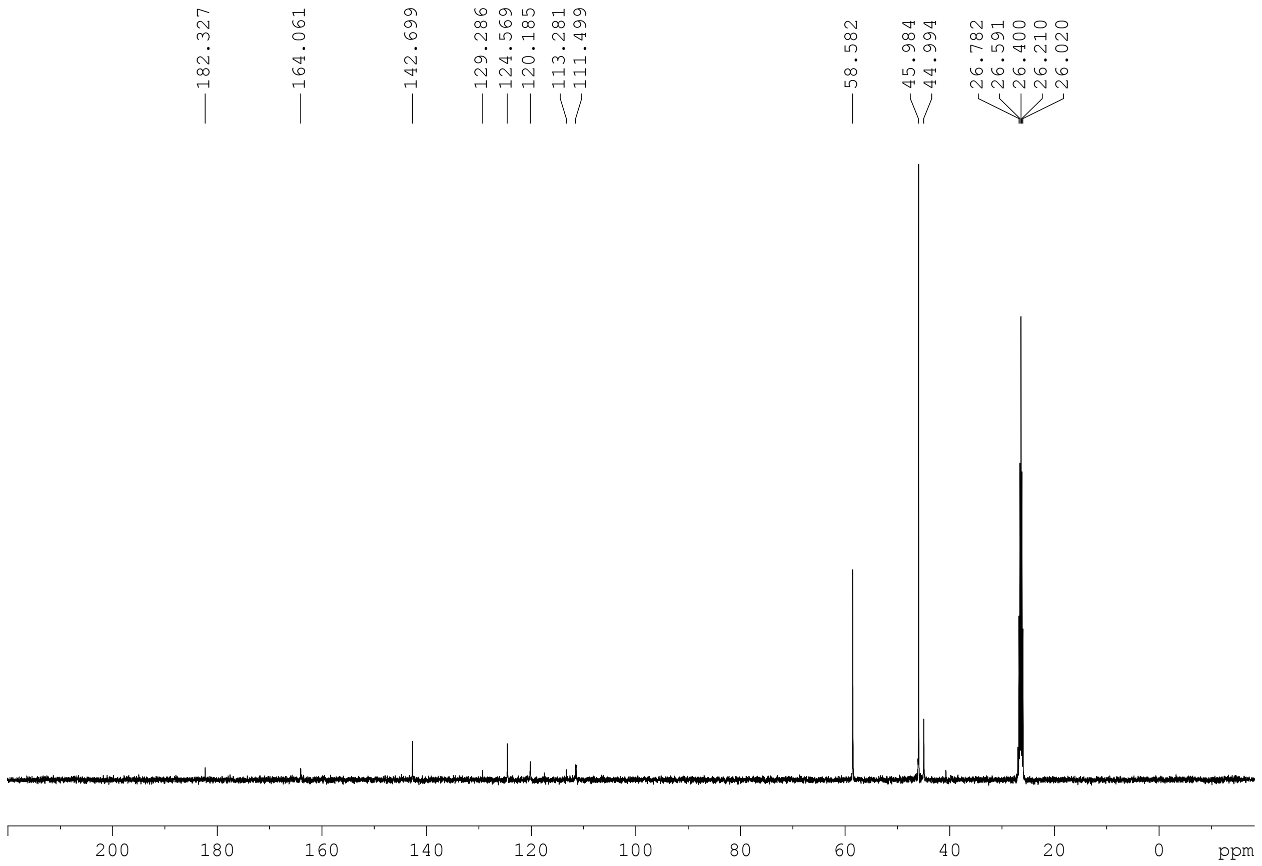

Spectrum 9. $^{13}C\{^1H\}$ NMR (100.62 MHz, 300 K) spectrum of **3** in C_6D_{12} solution.

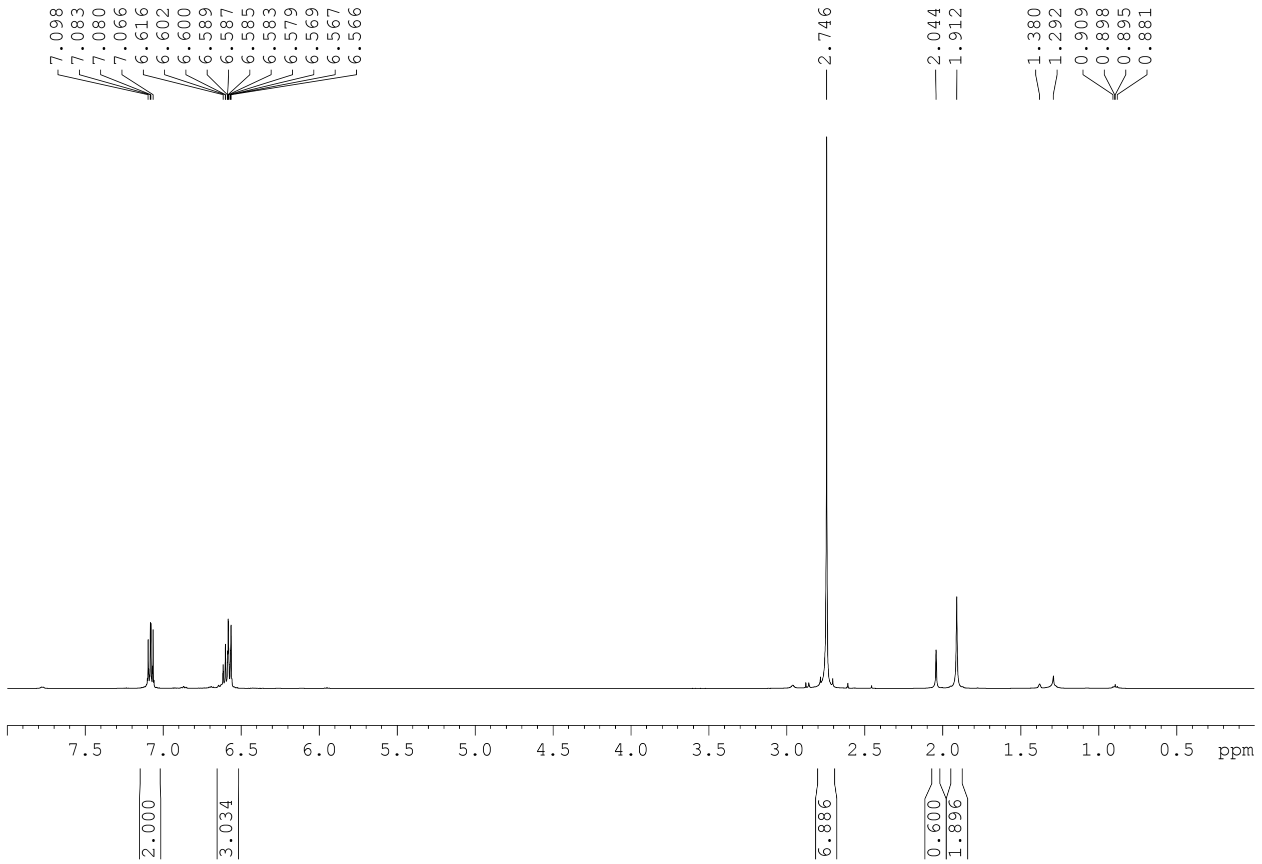

Spectrum 10. ^{1}H NMR (400.13 MHz, 300 K) spectrum of mother liquor following isolation of **3** in C_6D_{12} solution.

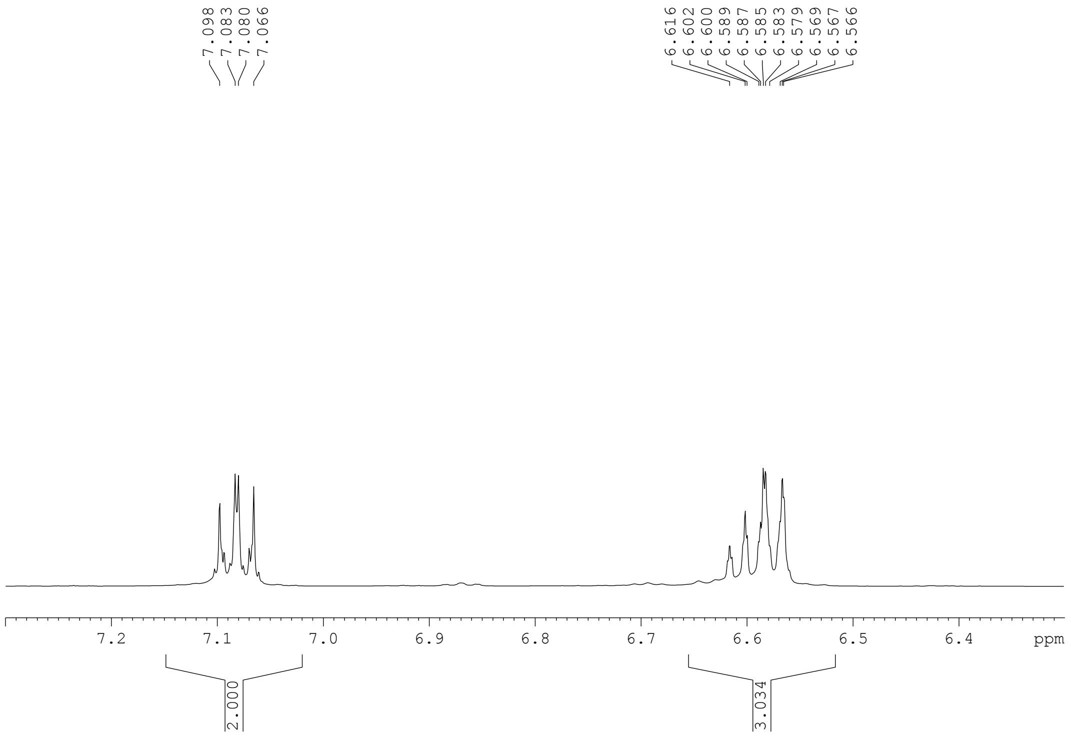

Spectrum 11. Aromatic region of ^{1}H NMR spectrum of mother liquor following isolation of **3** in C_6D_{12} solution.

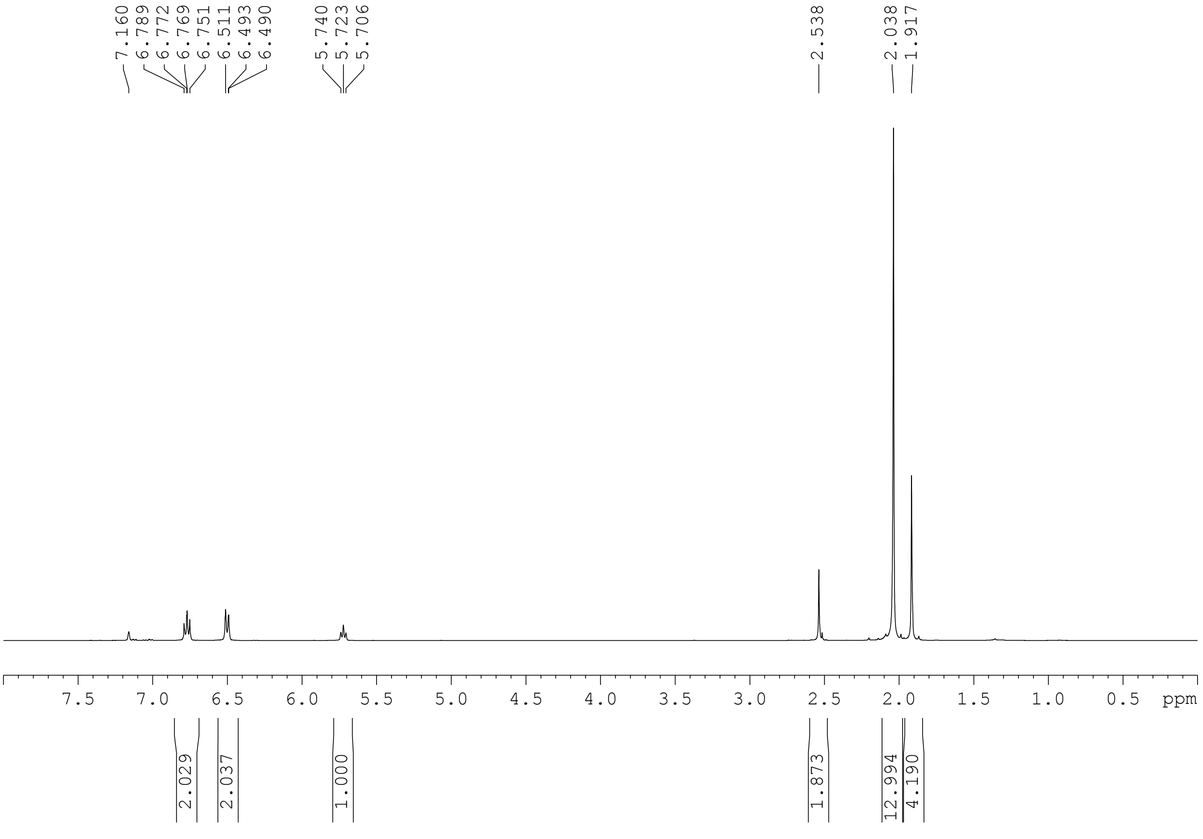

Spectrum 12. ^{1}H NMR (400.13 MHz, 300 K) spectrum from the reaction of **3** with toluene in C_6D_6 solution.

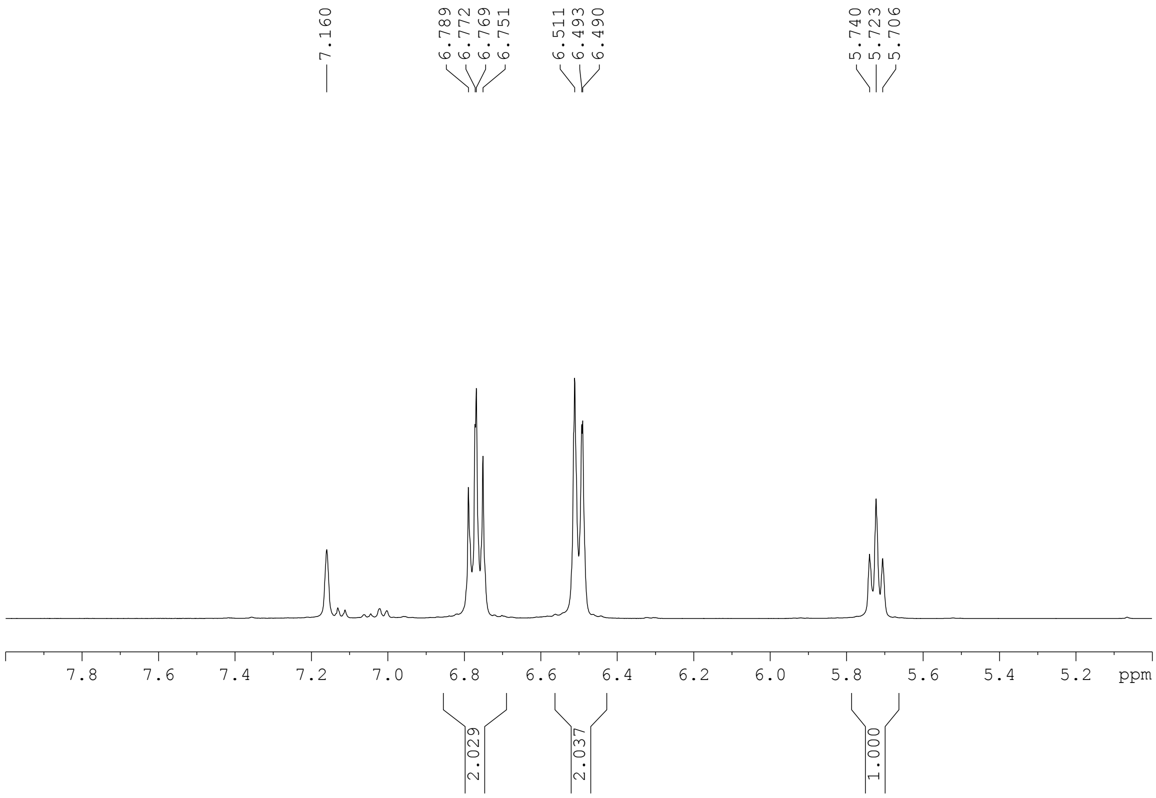

Spectrum 13. Aromatic region of ^{1}H NMR spectrum from the reaction of **3** with toluene in C_6D_6 solution.

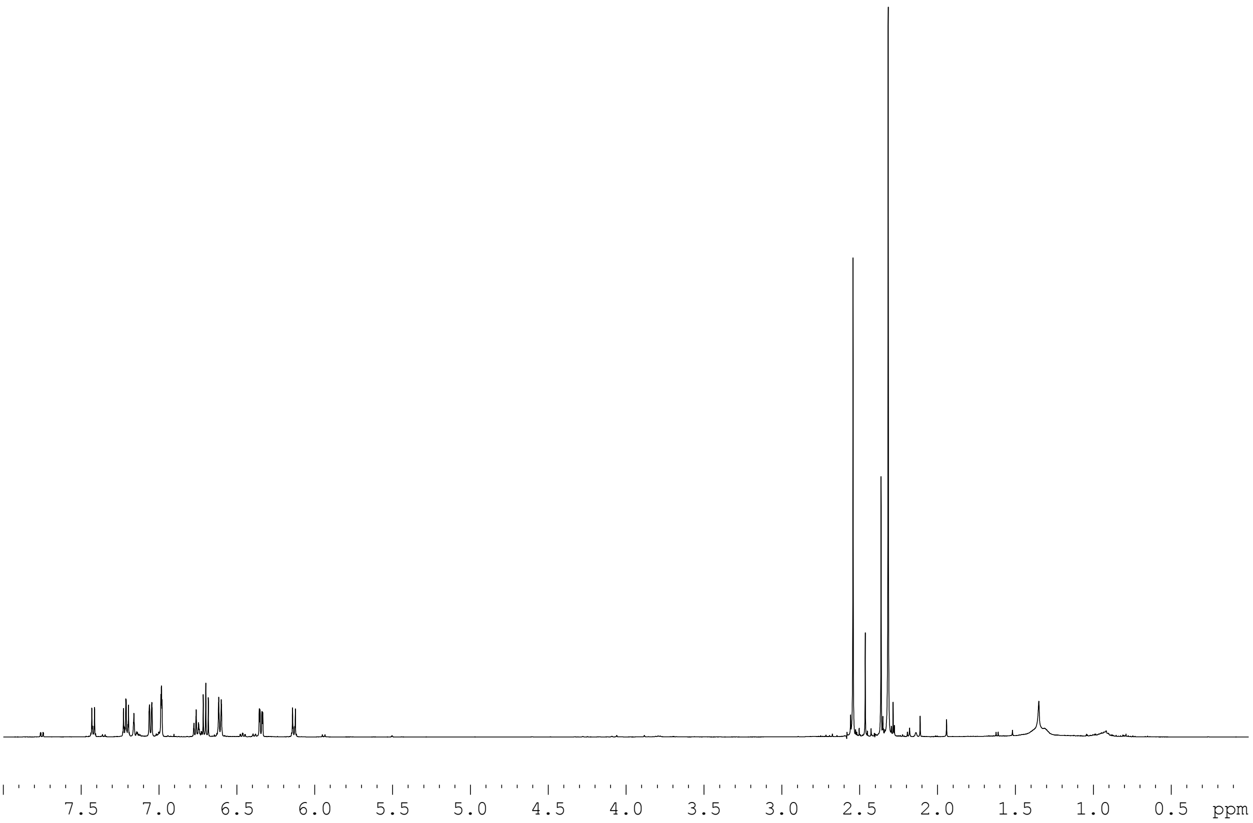

Spectrum 14. ^{1}H NMR (400.13 MHz, 300 K) spectrum from the reaction of **1** and *N*,*N*-dimethylaniline with iodine in C_6D_6 solution.

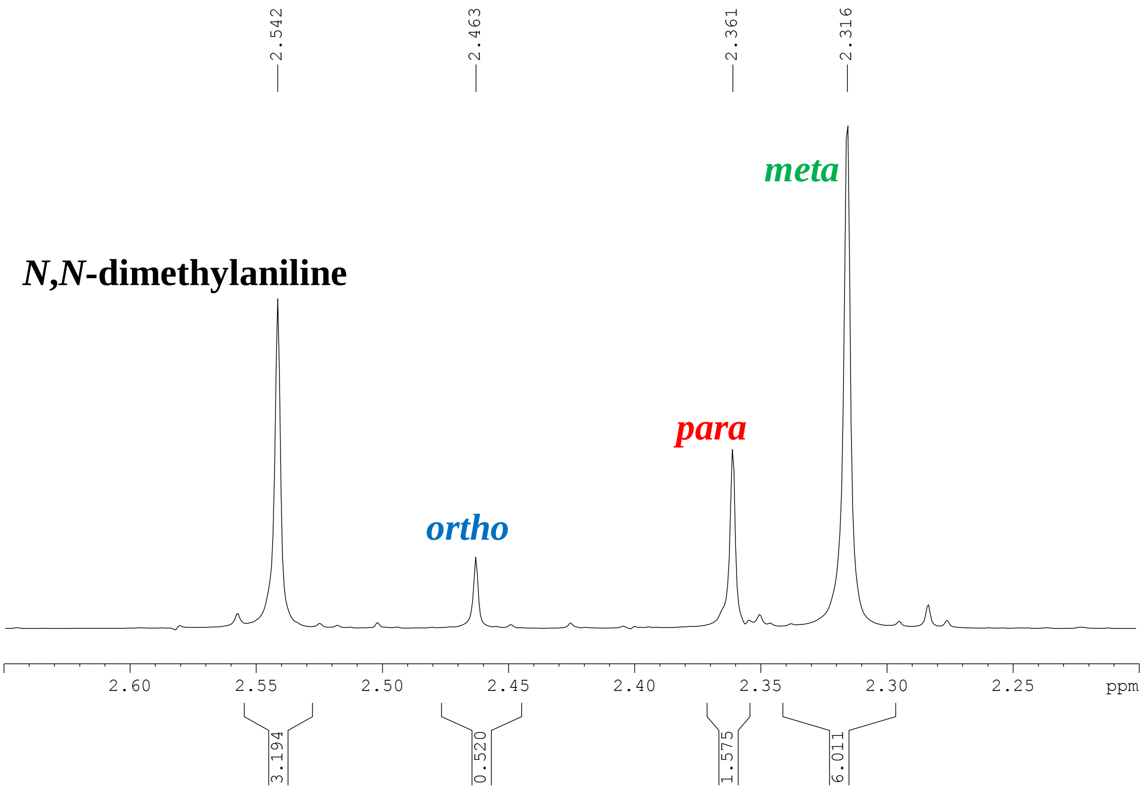

Spectrum 15. Aliphatic region of ^{1}H NMR spectrum from the reaction of **1** and *N*,*N*-dimethylaniline with iodine in C_6D_6 solution.

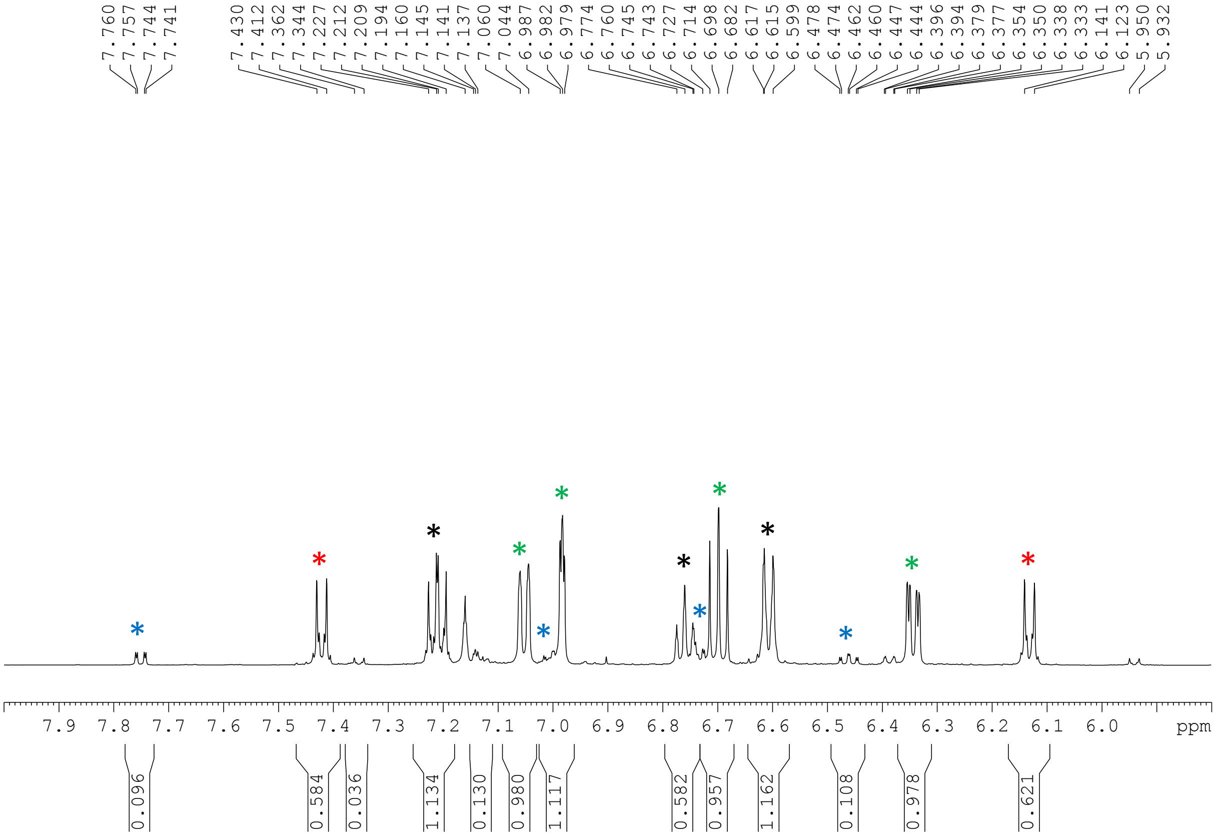

Spectrum 16. Aromatic region of ^{1}H NMR spectrum from the reaction of **1** and *N,N*-dimethylaniline with iodine in C_6D_6 solution.

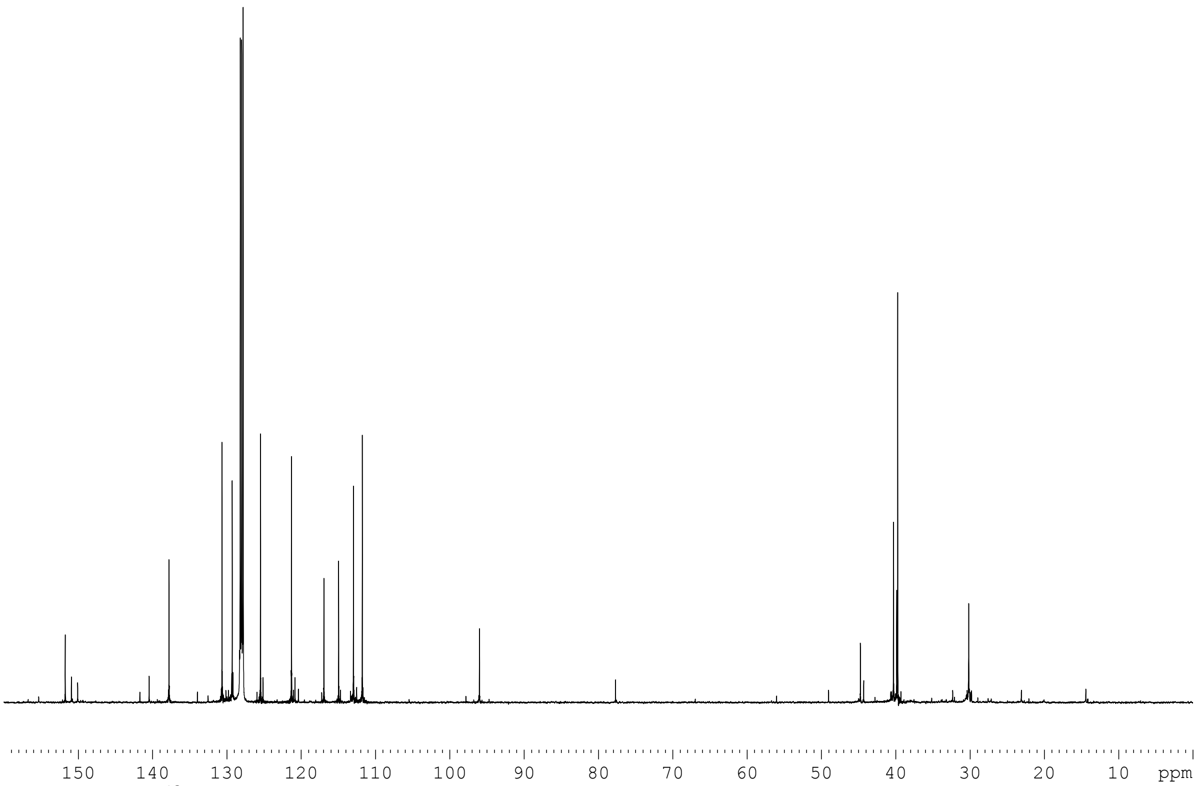

Spectrum 17. ^{13}C NMR (100.62 MHz, 300 K) spectrum from the reaction of **1** and *N*,*N*-dimethylaniline with iodine in C_6D_6 solution.

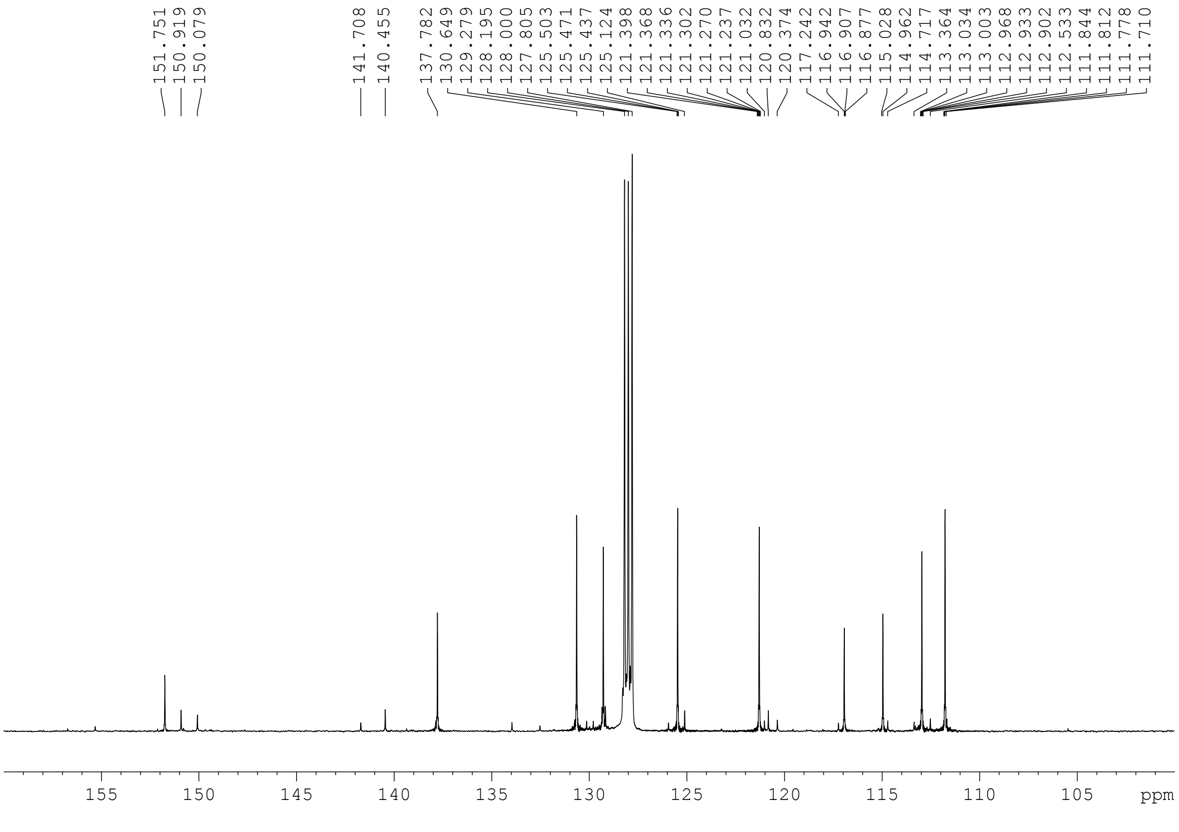

Spectrum 18. Aromatic region of ^{13}C NMR spectrum from the reaction of **1** and *N*,*N*-dimethylaniline with iodine in C_6D_6 solution.

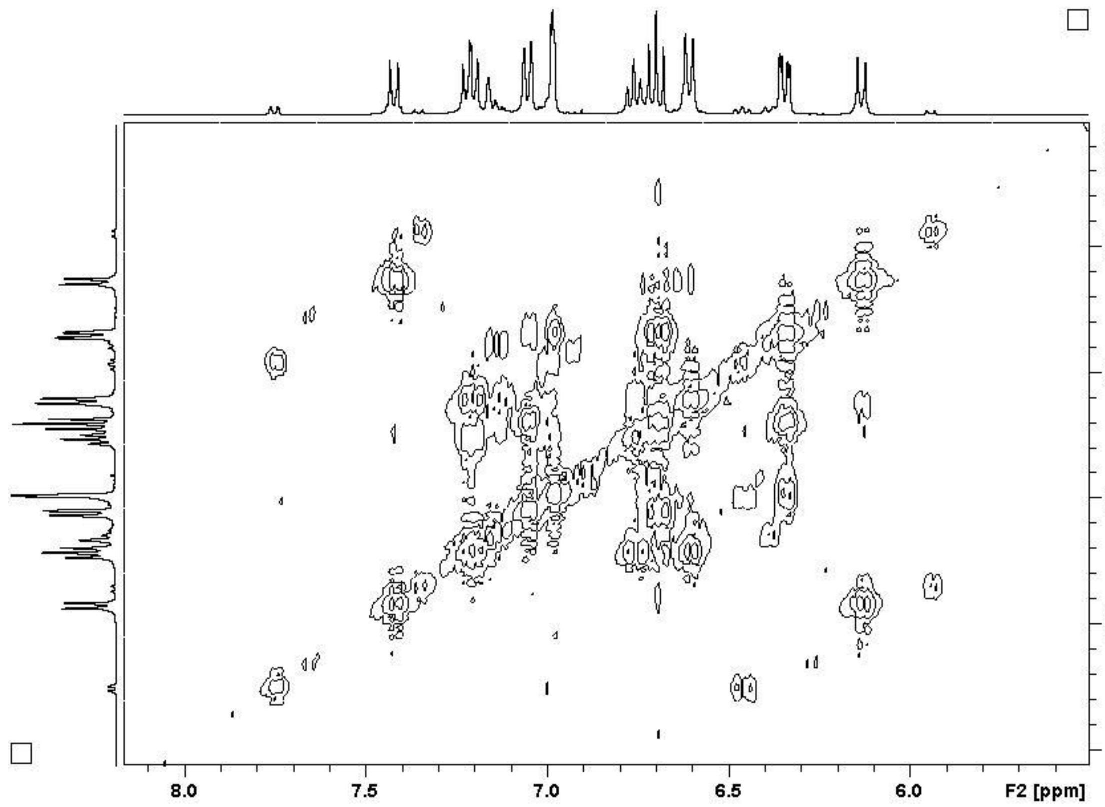

Spectrum 19. Aromatic region of ^1H-^1H COSY NMR spectrum from the reaction of **1** and *N*,*N*-dimethylaniline with iodine in C_6D_6 solution.

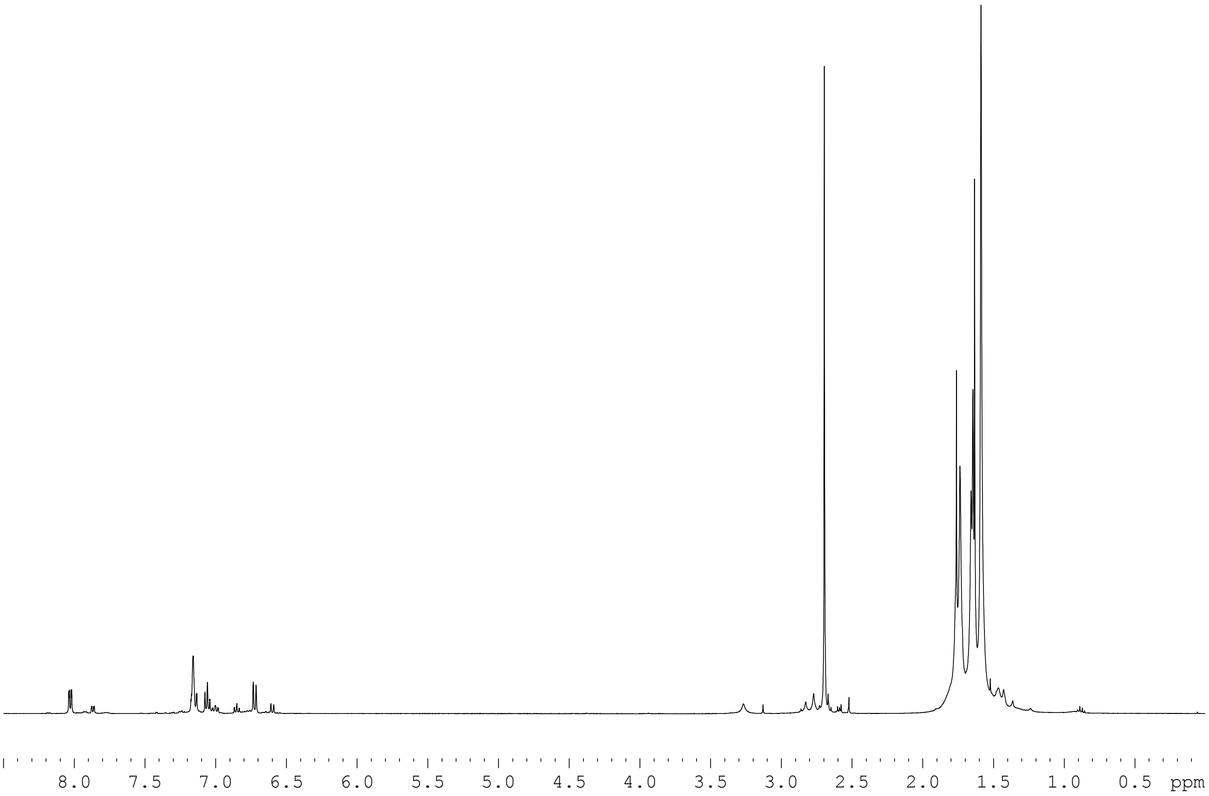

Spectrum 20. ^1H NMR (400.13 MHz, 300 K) spectrum of **4** and **5** in C_6D_6 solution.

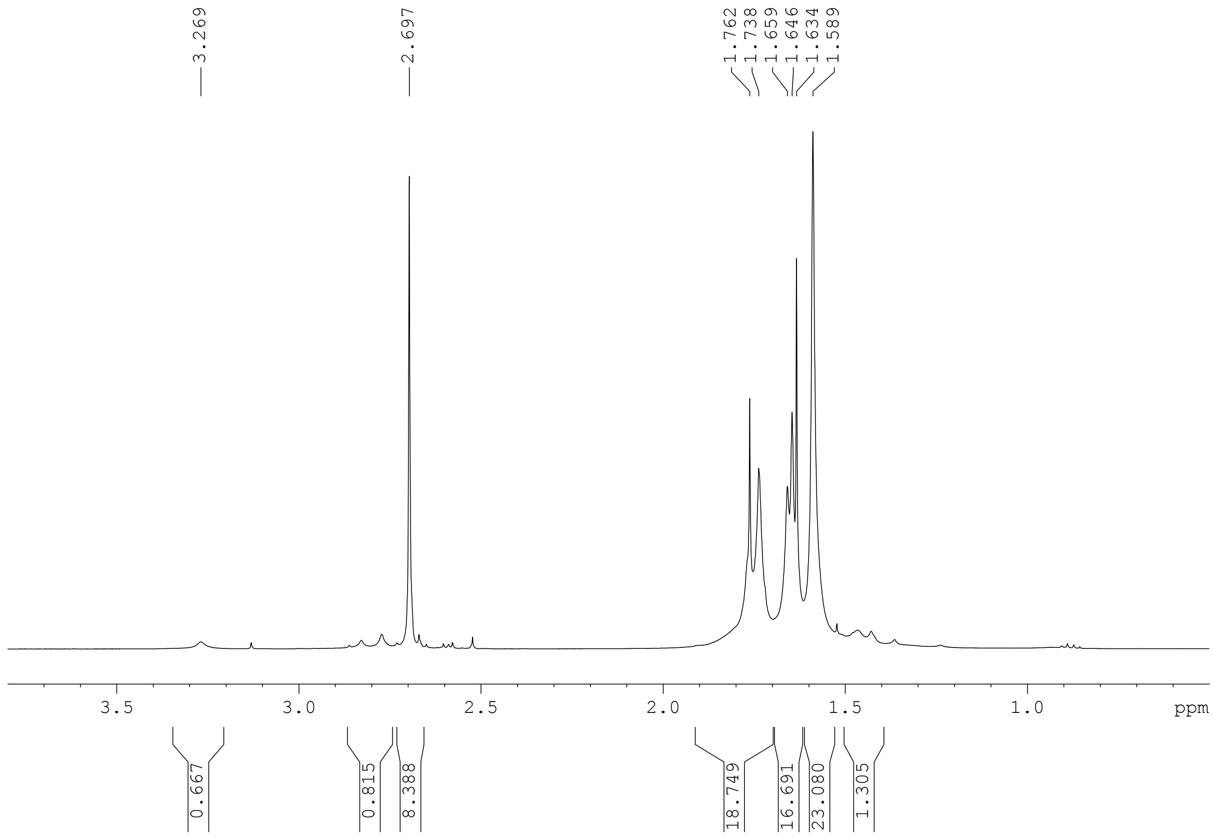

Spectrum 21. Aliphatic region of ^{1}H NMR spectrum of **4** and **5** in C_6D_6 solution.

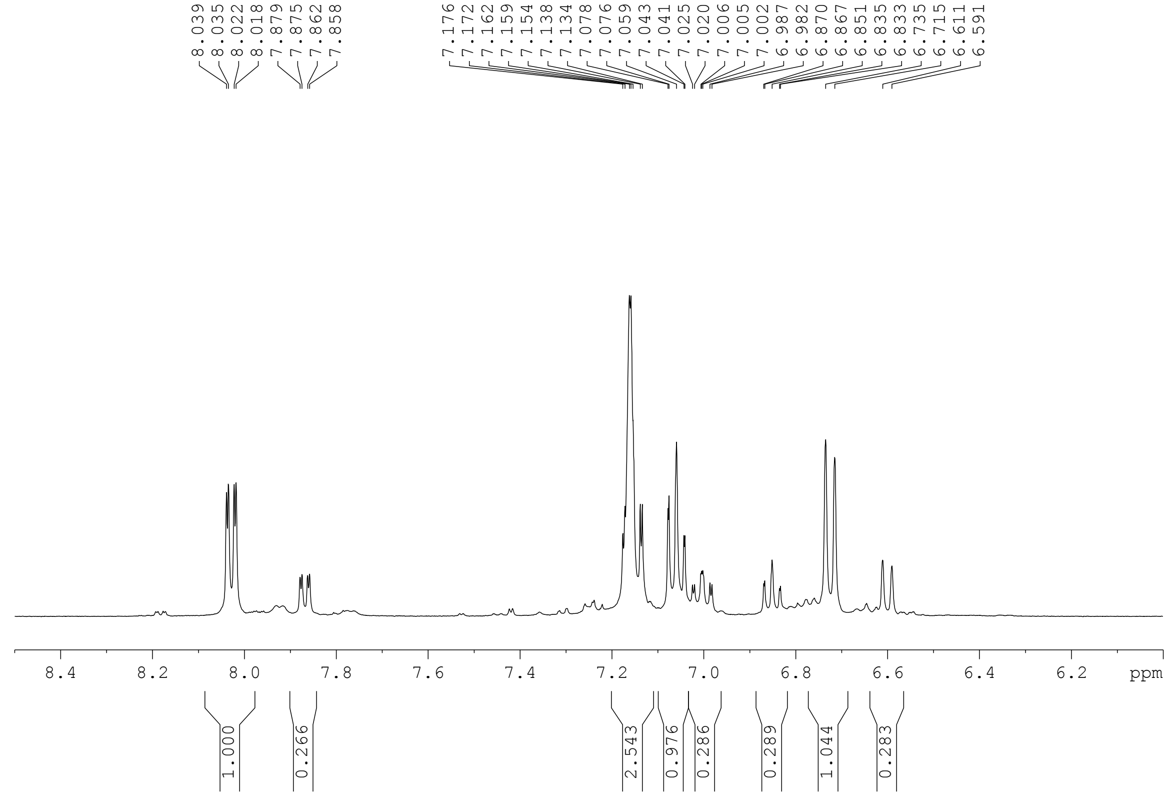

Spectrum 22. Aromatic region of ^{1}H NMR spectrum of **4** and **5** in C_6D_6 solution.

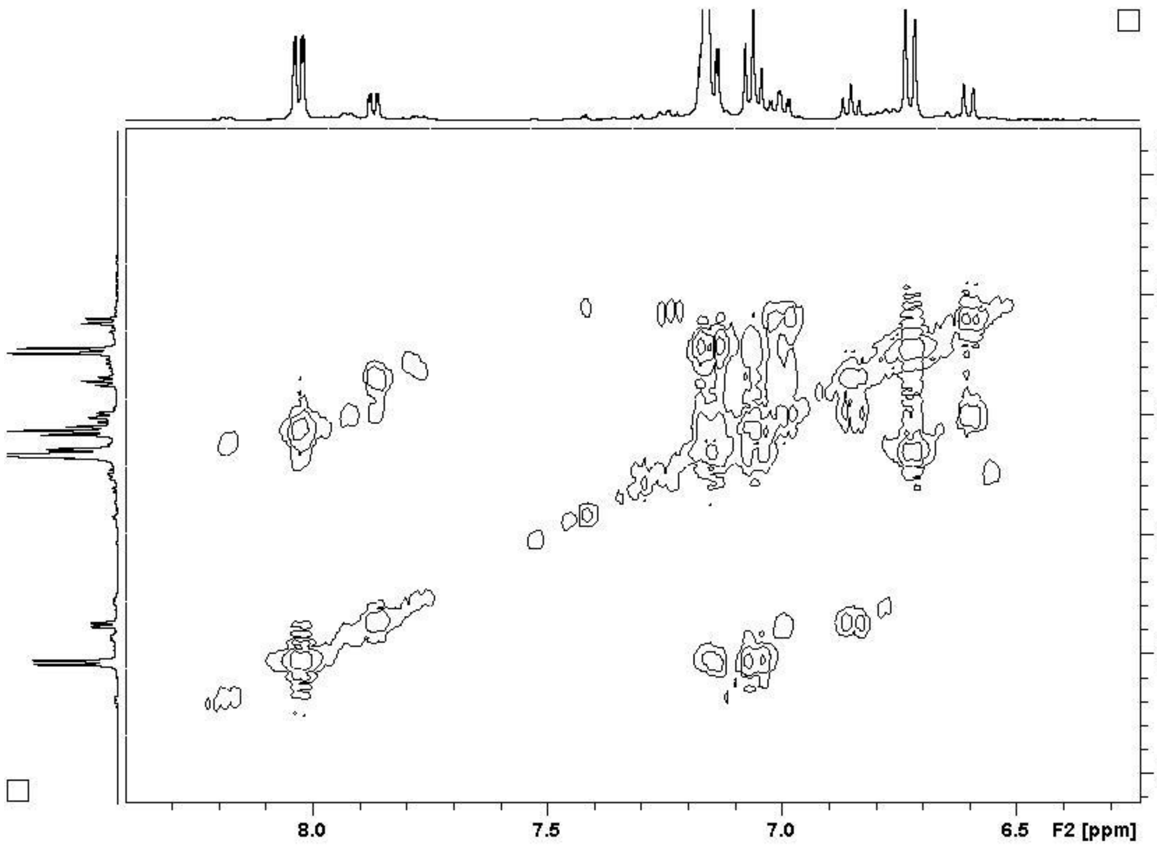

Spectrum 23. Aromatic region of ^{1}H-^{1}H COSY NMR spectrum of **4** and **5** in C_6D_6 solution.

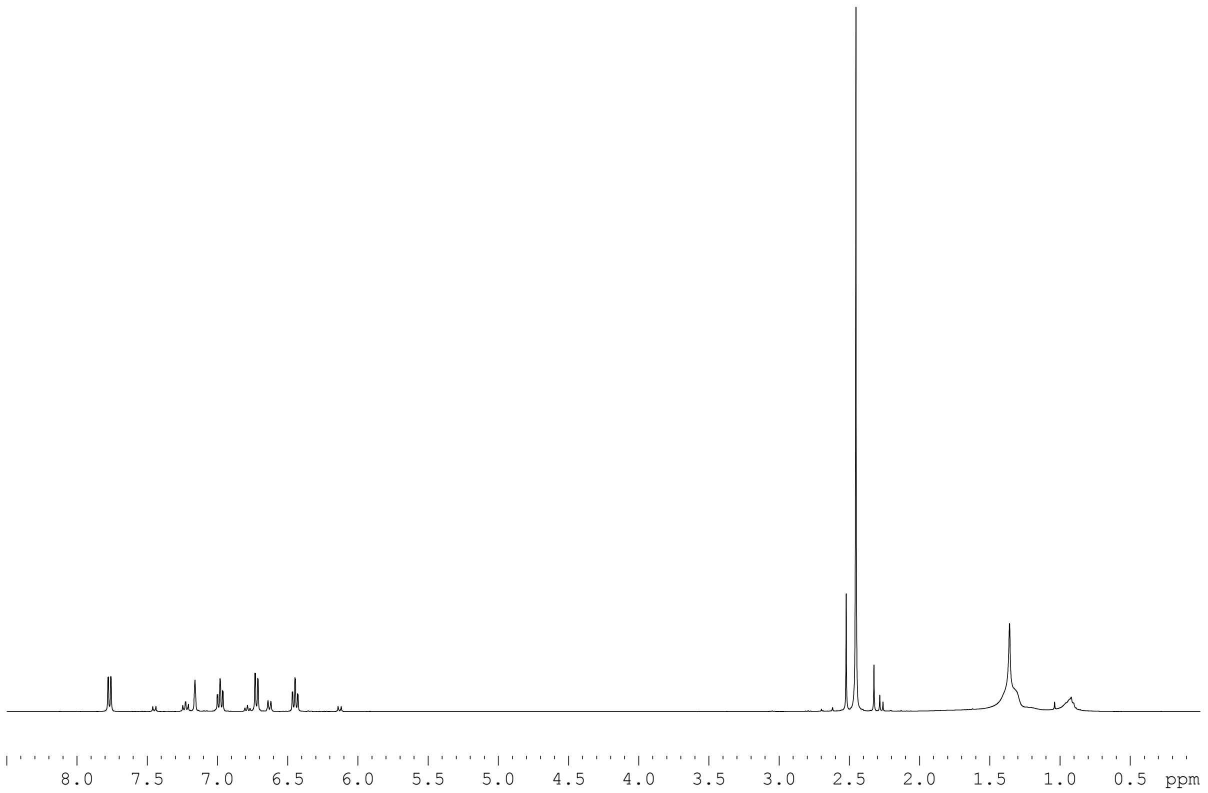

Spectrum 24. ^{1}H NMR (400.13 MHz, 300 K) spectrum from the reaction of BuNa·TMEDA, *N*,*N*-dimethylaniline and *t*Bu_2Zn with iodine in C_6D_6 solution.

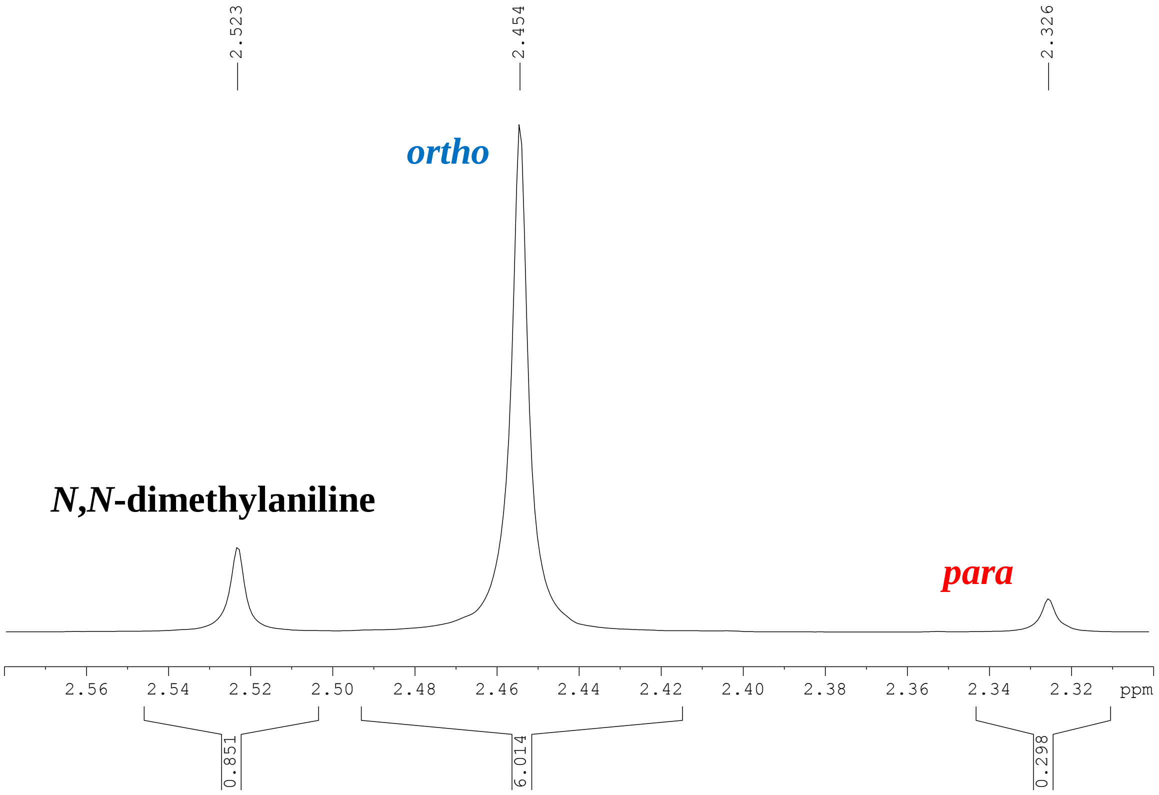

Spectrum 25. Aliphatic region of ^{1}H NMR spectrum from the reaction of BuNa·TMEDA, *N*,*N*-dimethylaniline and *t*Bu_2Zn with iodine in C_6D_6 solution.

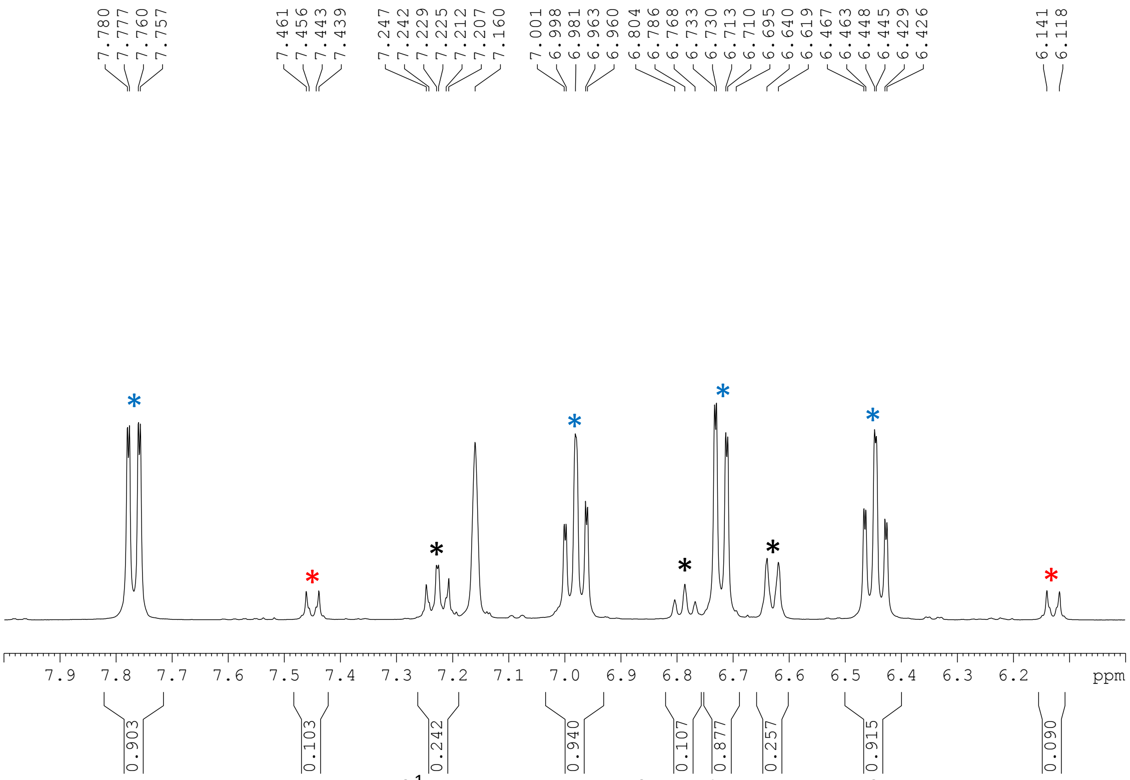

Spectrum 26. Aromatic region of ^{1}H NMR spectrum from the reaction of BuNa·TMEDA, *N*,*N*-dimethylaniline and *t*Bu_2Zn with iodine in C_6D_6 solution.

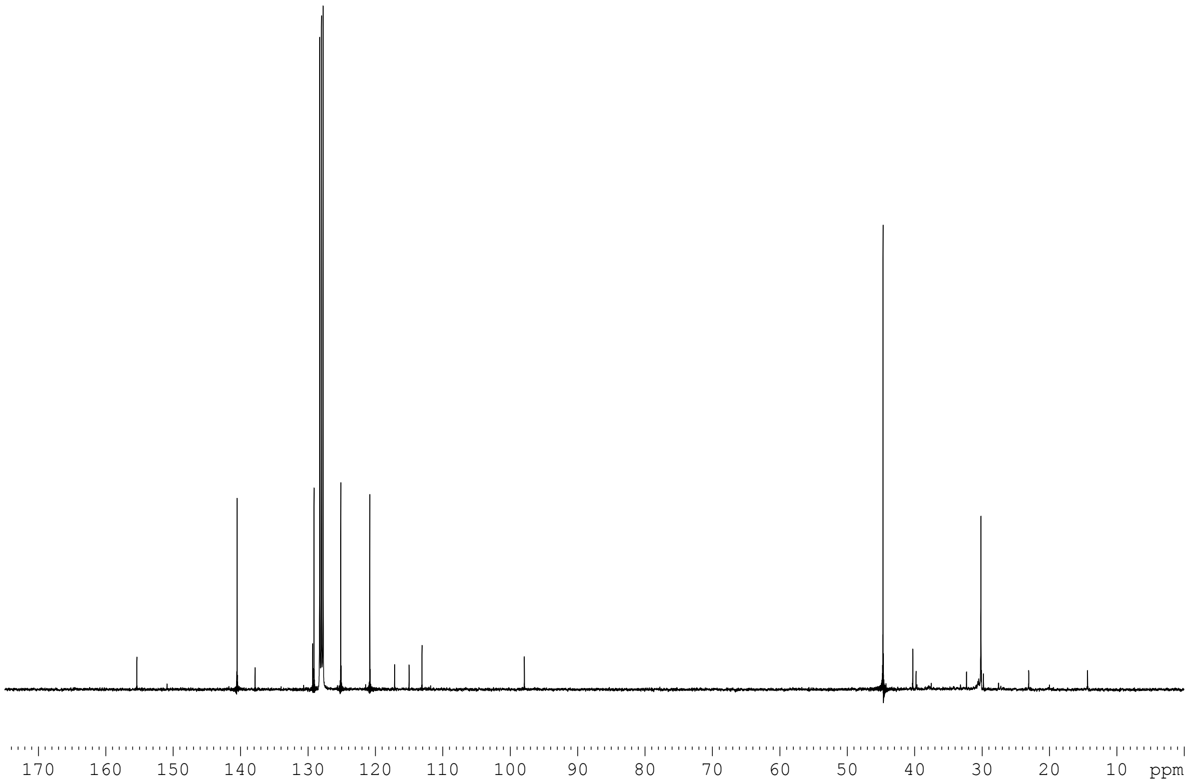

Spectrum 27. ^{13}C NMR (100.62 MHz, 300 K)spectrum from the reaction of BuNa·TMEDA, *N*,*N*-dimethylaniline and *t*Bu_2Zn with iodine in C_6D_6 solution.

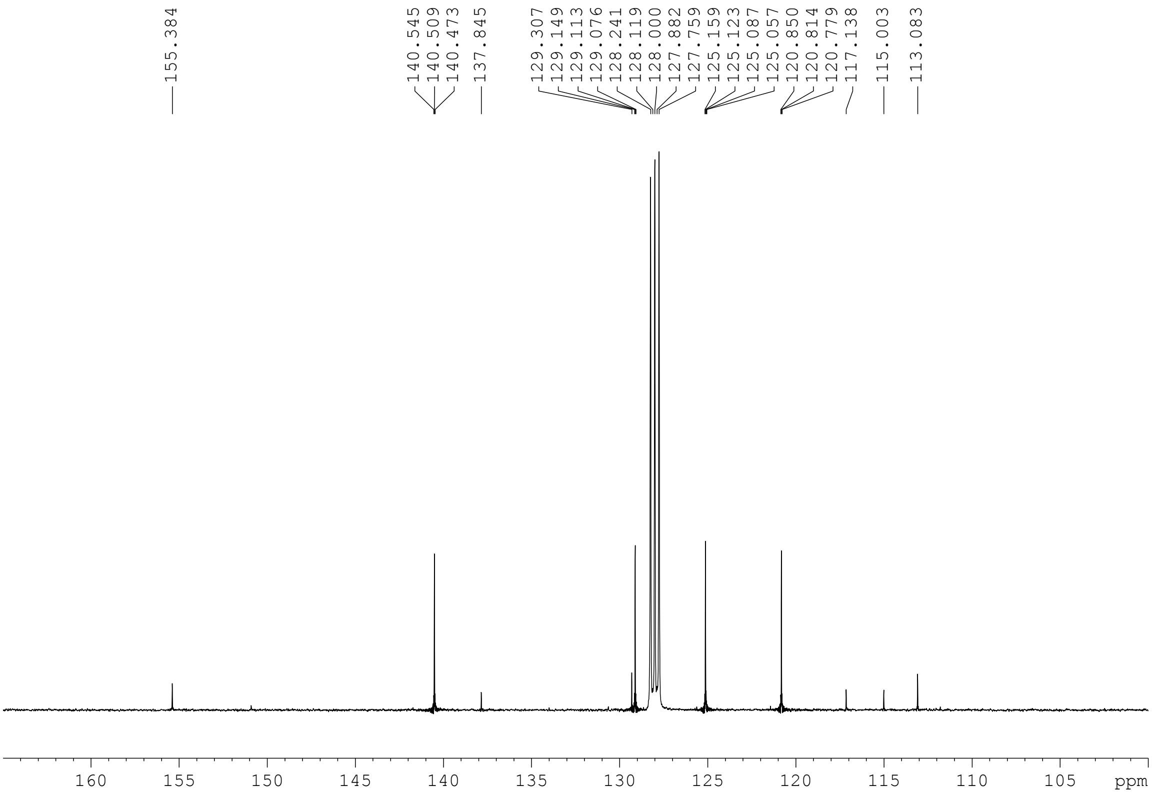

Spectrum 28. Aromatic region of ^{13}C NMR spectrum from the reaction of BuNa·TMEDA, *N*,*N*-dimethylaniline and *t*Bu_2Zn with iodine in C_6D_6 solution.

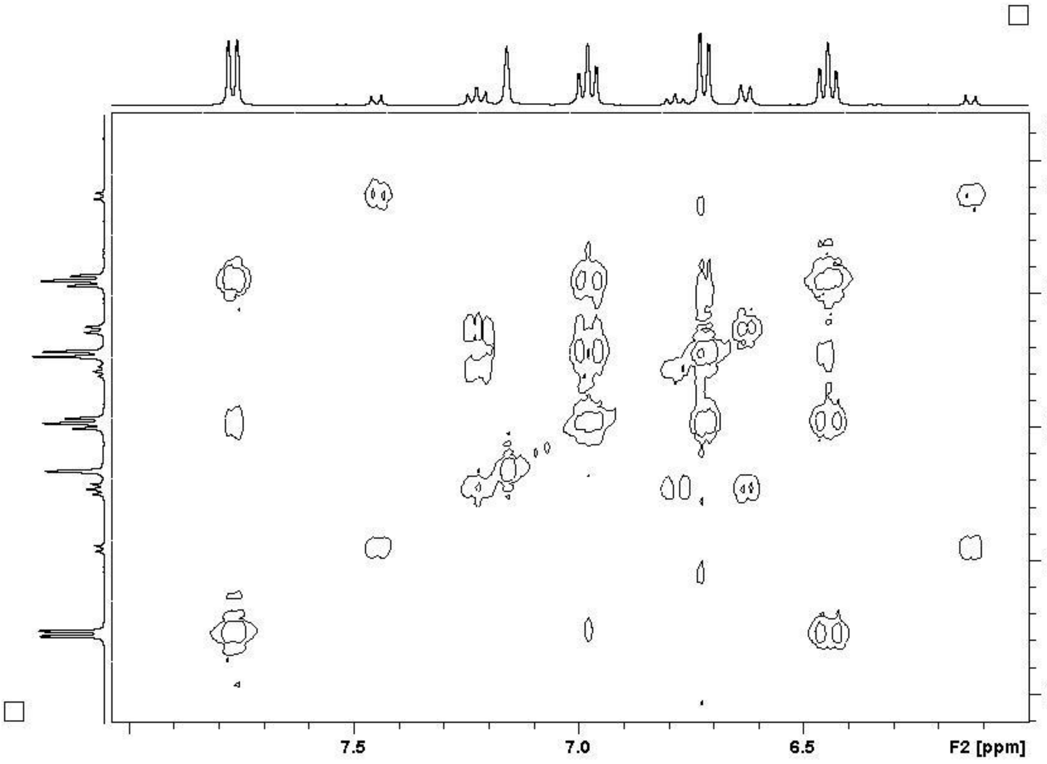

Spectrum 29. Aromatic region of ^{1}H-^{1}H COSY NMR spectrum from the reaction of BuNa·TMEDA, *N*,*N*-dimethylaniline and *t*Bu_2Zn with iodine in C_6D_6 solution.

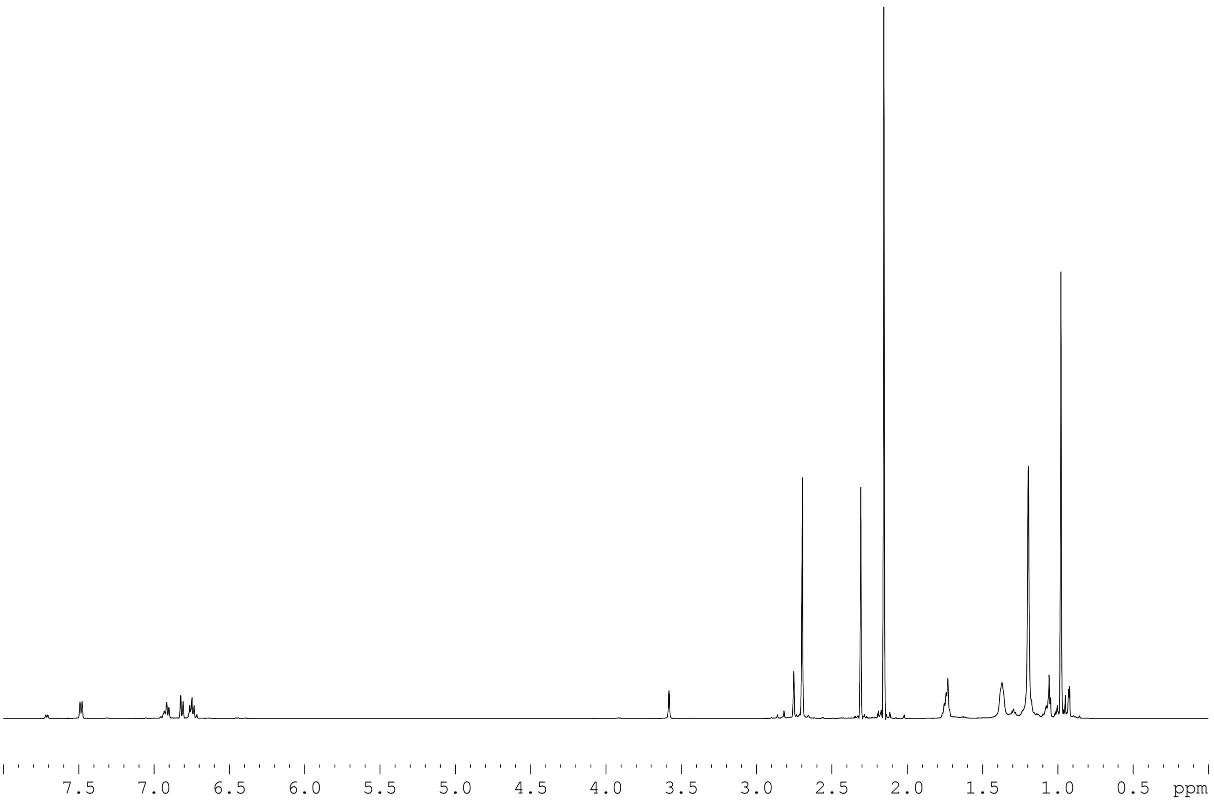

Spectrum 30. ^{1}H NMR (400.13 MHz, 300 K) spectrum of **6** in d_8-THF solution.

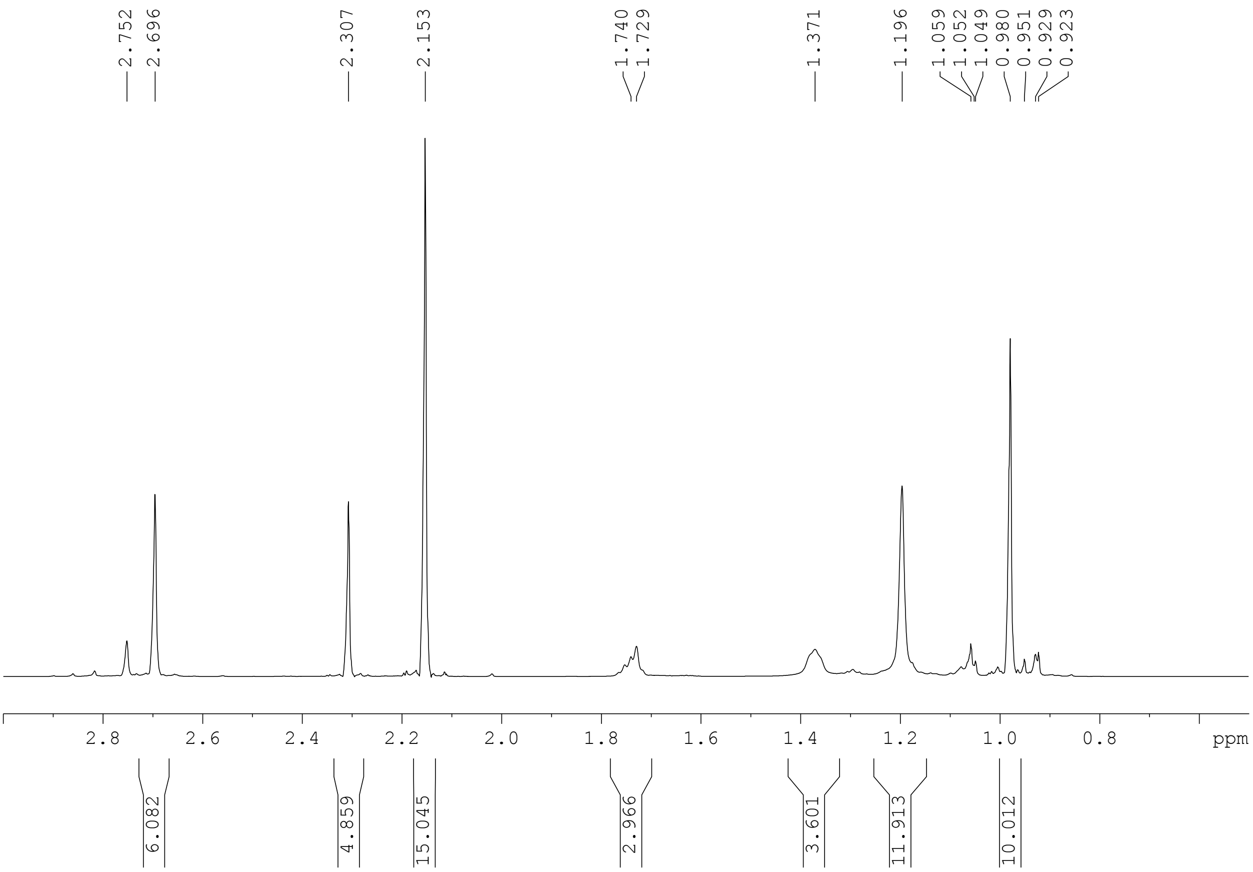

Spectrum 31. Aliphatic region of ^{1}H NMR spectrum of **6** in d_8-THF solution.

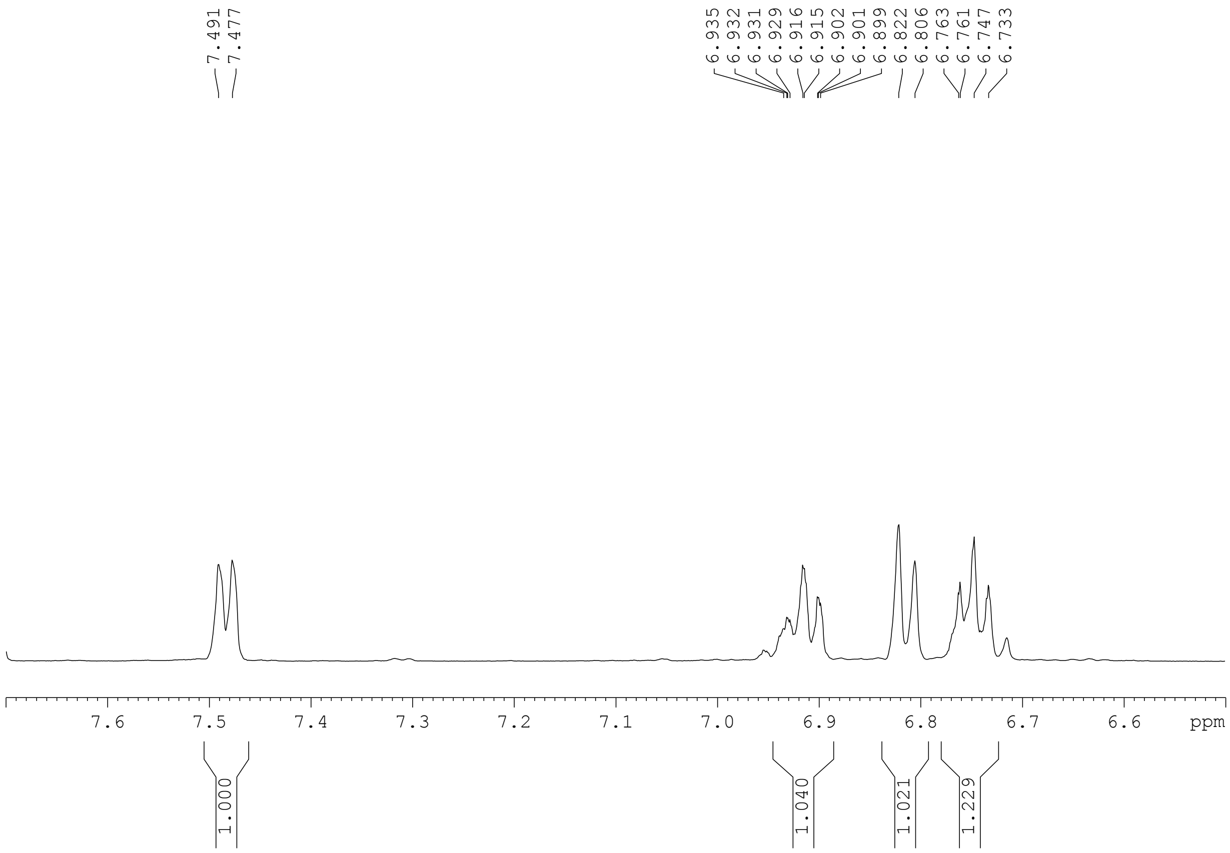

Spectrum 32. Aromatic region of ^{1}H NMR spectrum of **6** in d_8-THF solution.

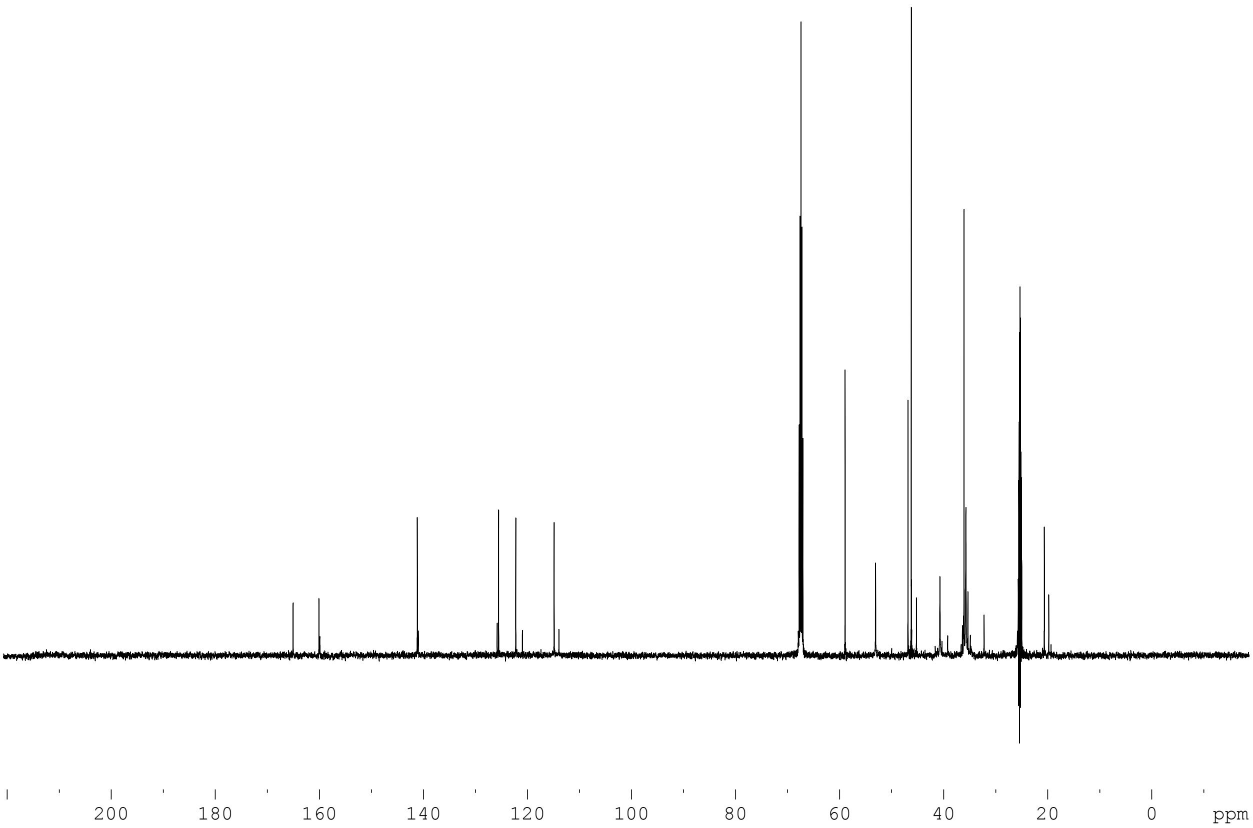

Spectrum 33. ^{13}C NMR (100.62 MHz, 300 K) spectrum of **6** in d_8-THF solution.

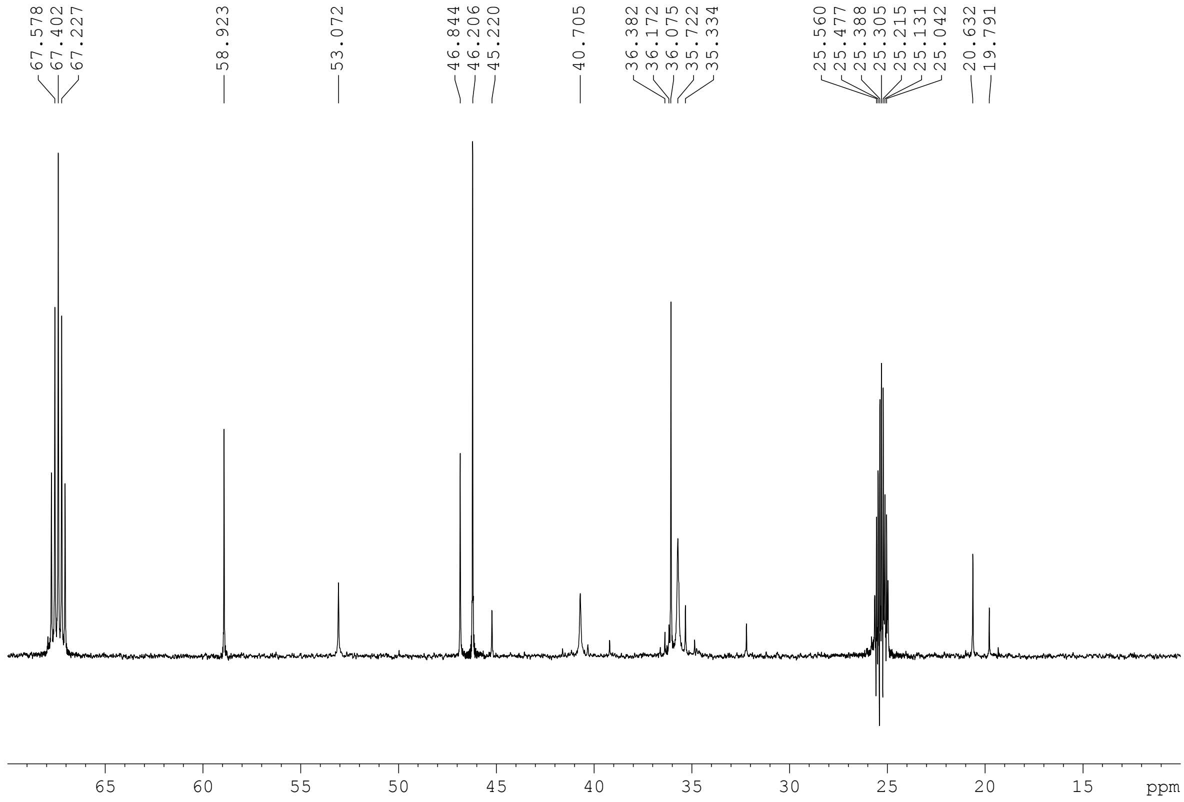

Spectrum 34. Aliphatic region of ^{13}C NMR spectrum of **6** in d_8-THF solution.

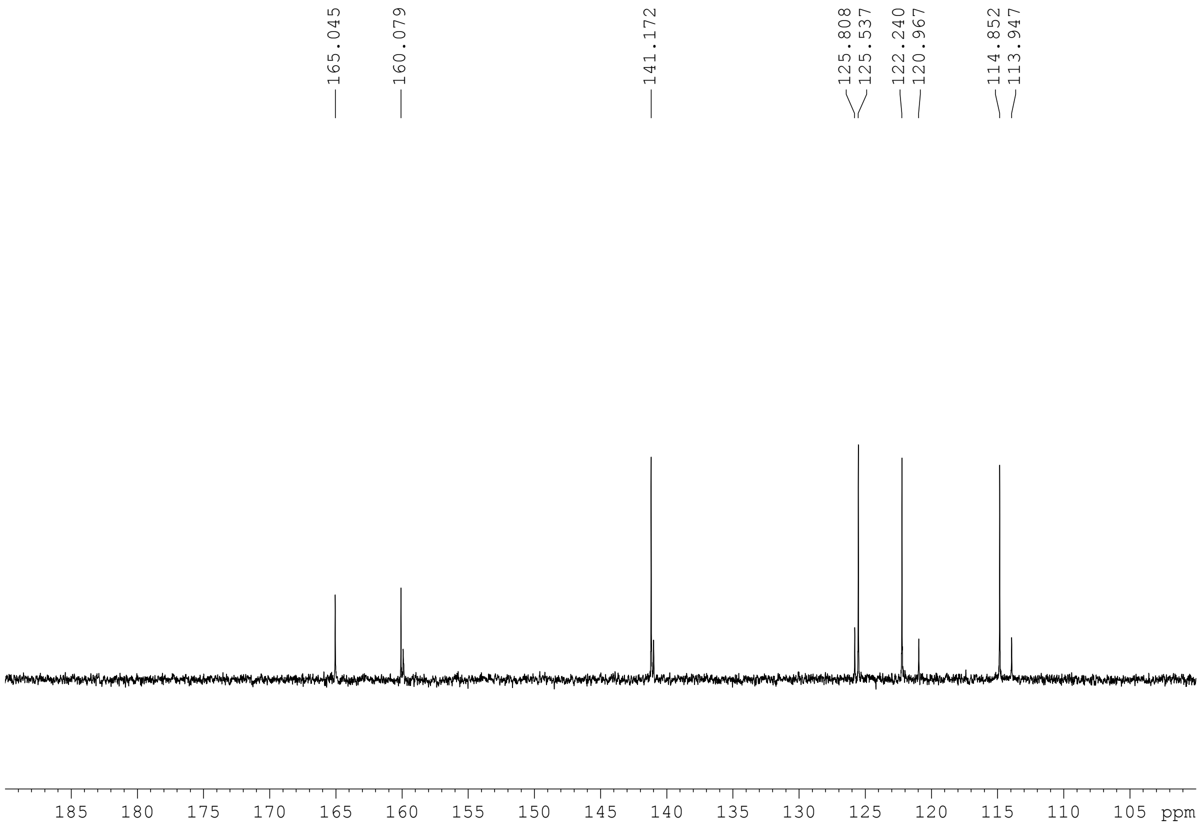

Spectrum 35. Aromatic region of ^{13}C NMR spectrum of **6** in d_8-THF solution.

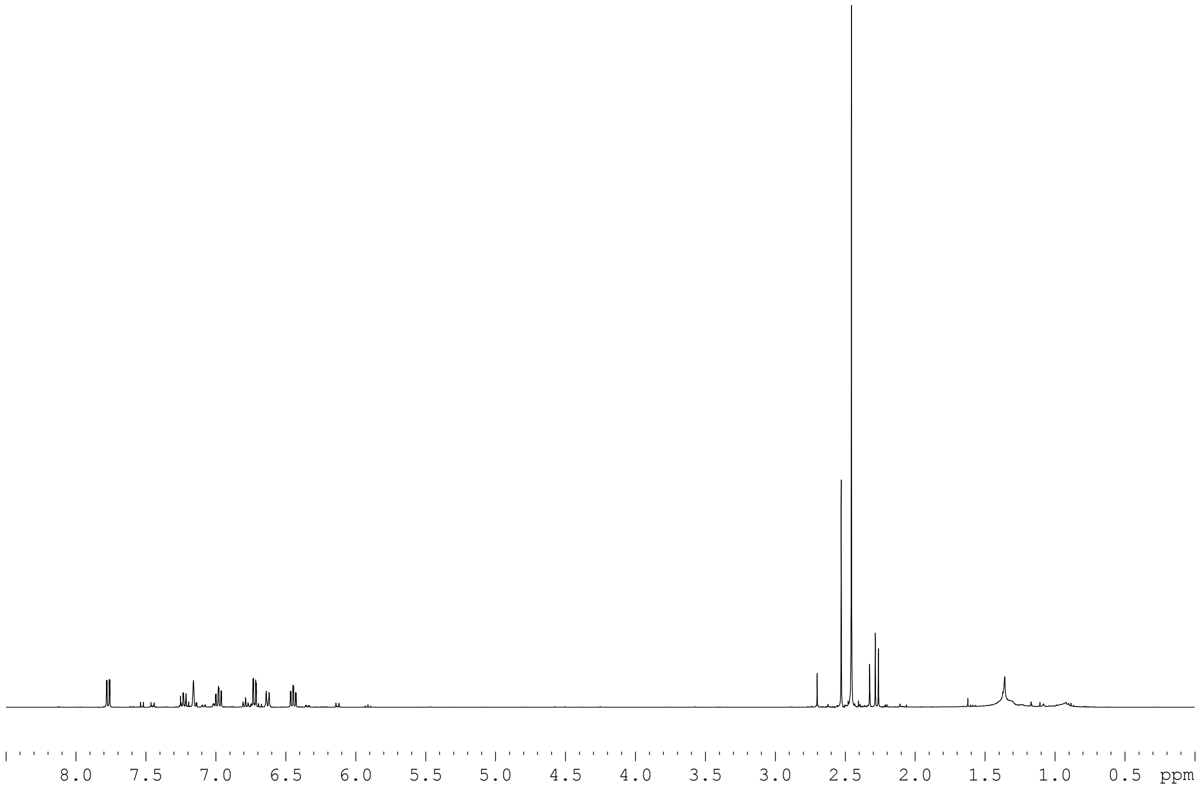

Spectrum 36. ^{1}H NMR (400.13 MHz, 300 K) from the reaction of BuNa·TMEDA, *N*,*N*-dimethylaniline, *t*Bu_2Zn and TMP(H) with iodine in C_6D_6 solution.

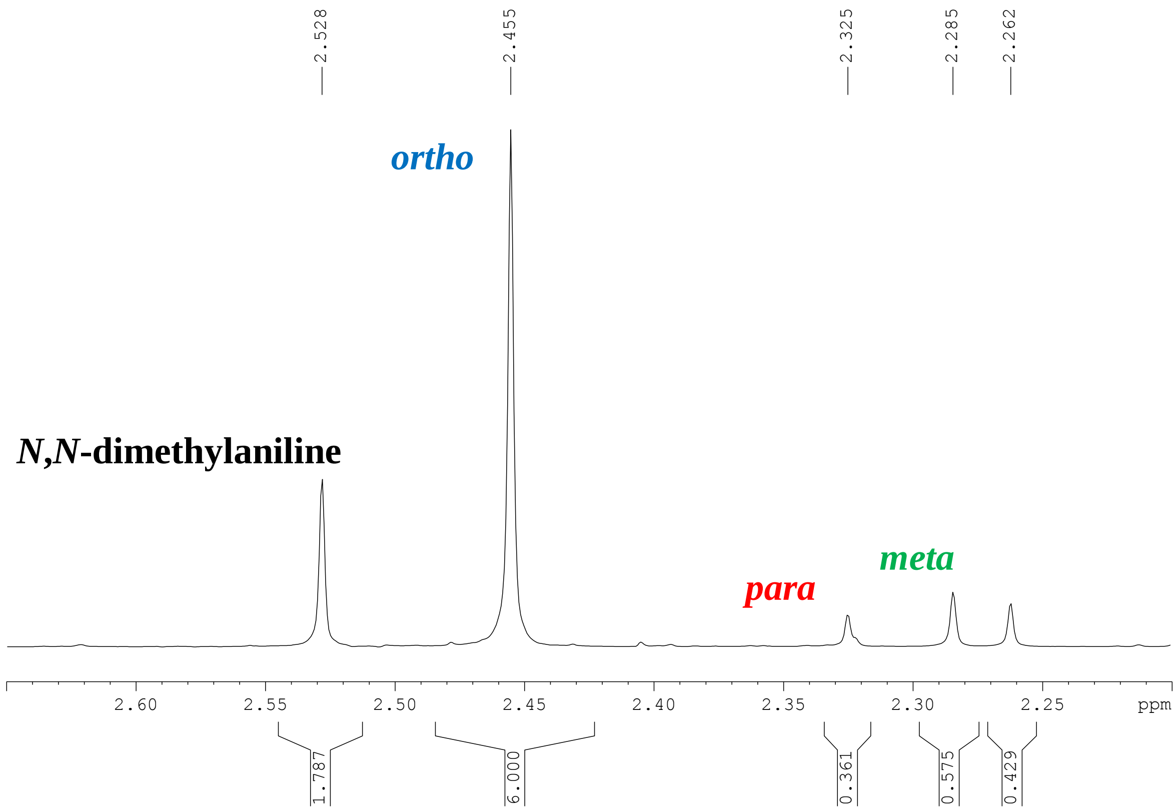

Spectrum 37. Aliphatic region of ^{1}H NMR spectrum from the reaction of BuNa·TMEDA, *N*,*N*-dimethylaniline, *t*Bu_2Zn and TMP(H) with iodine in C_6D_6 solution.

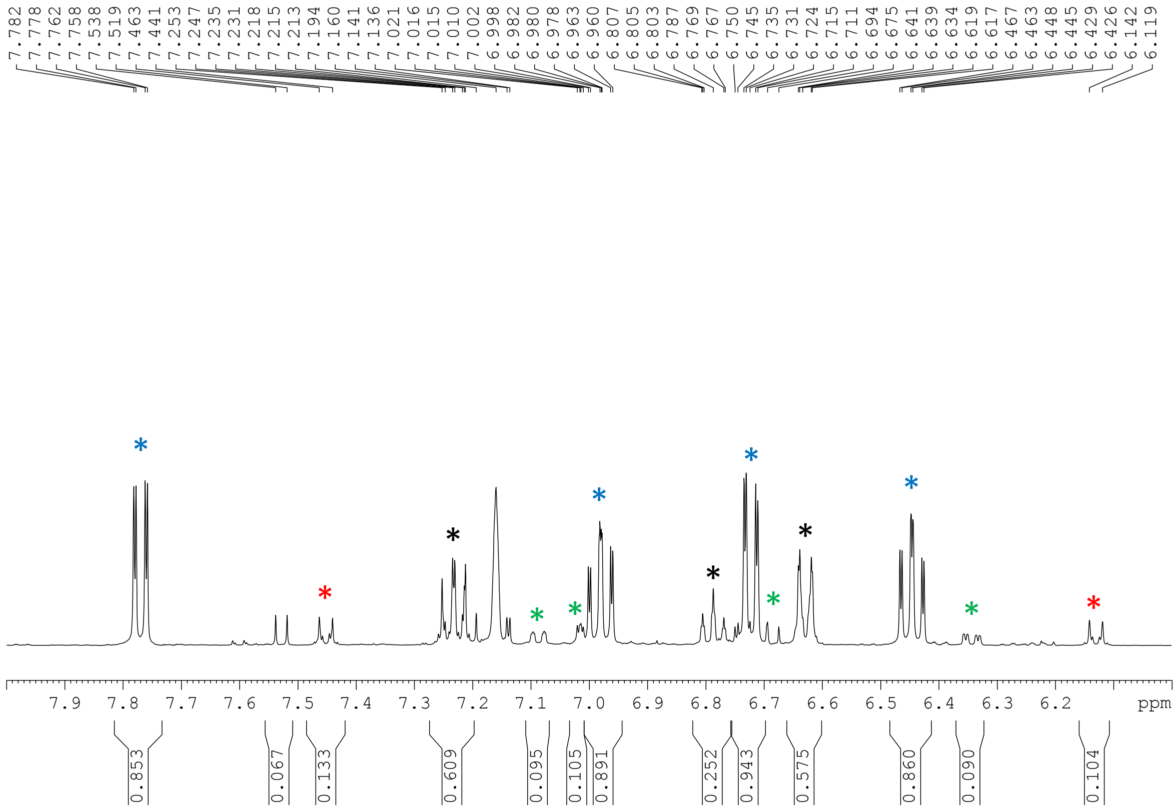

Spectrum 38. Aromatic region of ^{1}H NMR spectrum from the reaction of BuNa·TMEDA, *N*,*N*-dimethylaniline, *t*Bu_2Zn and TMP(H) with iodine in C_6D_6 solution.

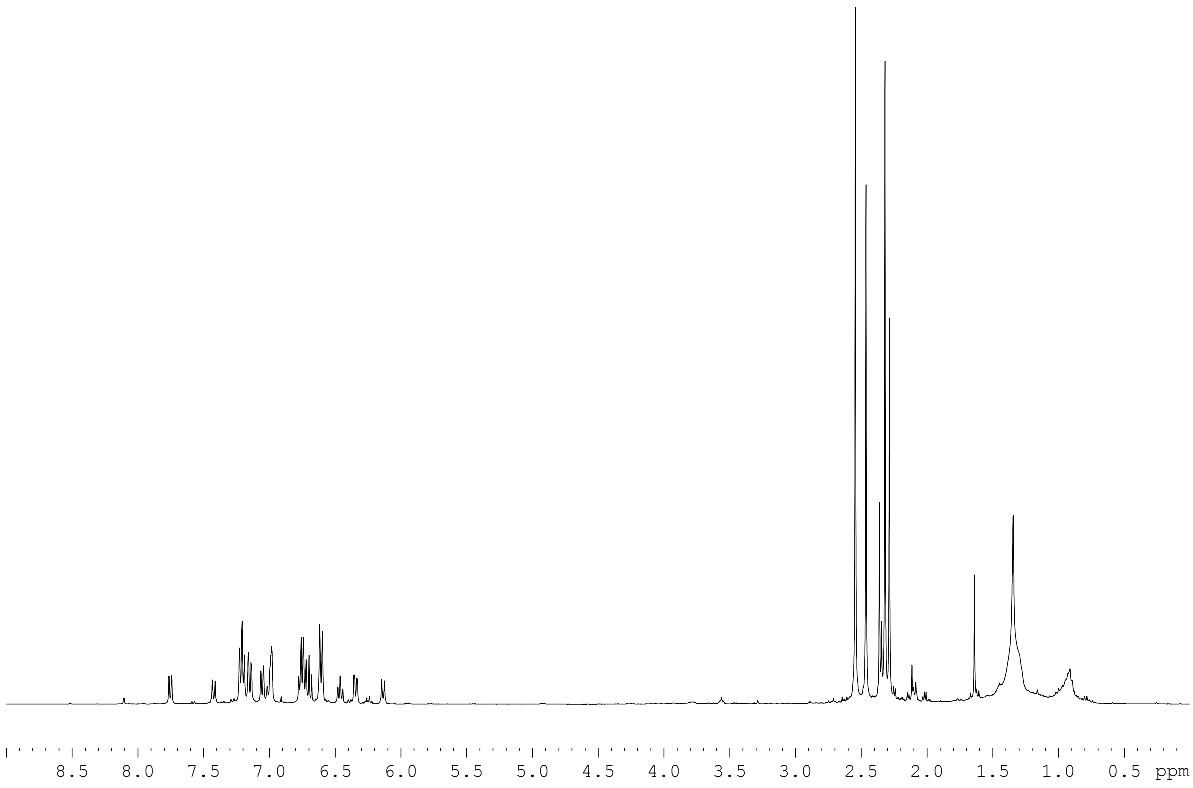

Spectrum 39. ^{1}H NMR (400.13 MHz, 300 K) spectrum from the reaction of BuNa·TMEDA, *N*,*N*-dimethylaniline, *t*Bu_2Zn and TMP(H) with iodine following an overnight reflux in C_6D_6 solution.

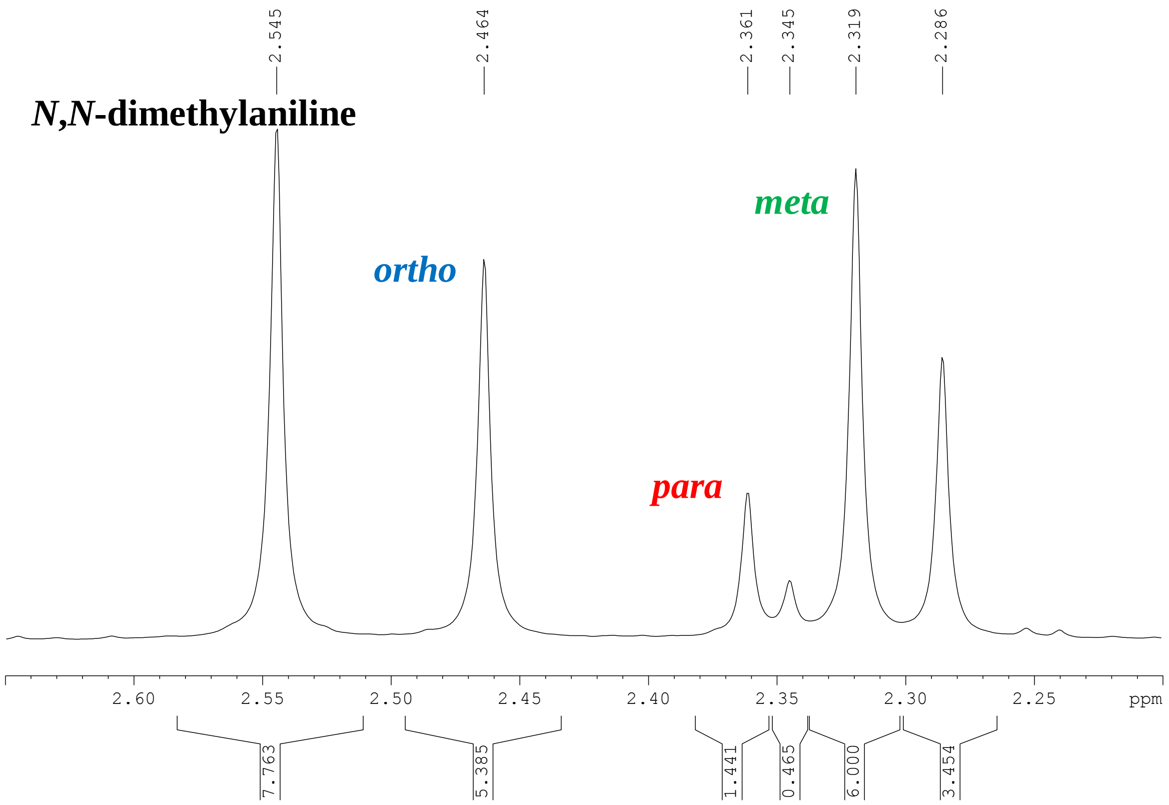

Spectrum 40. Aliphatic region of ^{1}H NMR spectrum from the reaction of BuNa·TMEDA, *N*,*N*-dimethylaniline, *t*Bu_2Zn and TMP(H) with iodine following an overnight reflux in C_6D_6 solution.

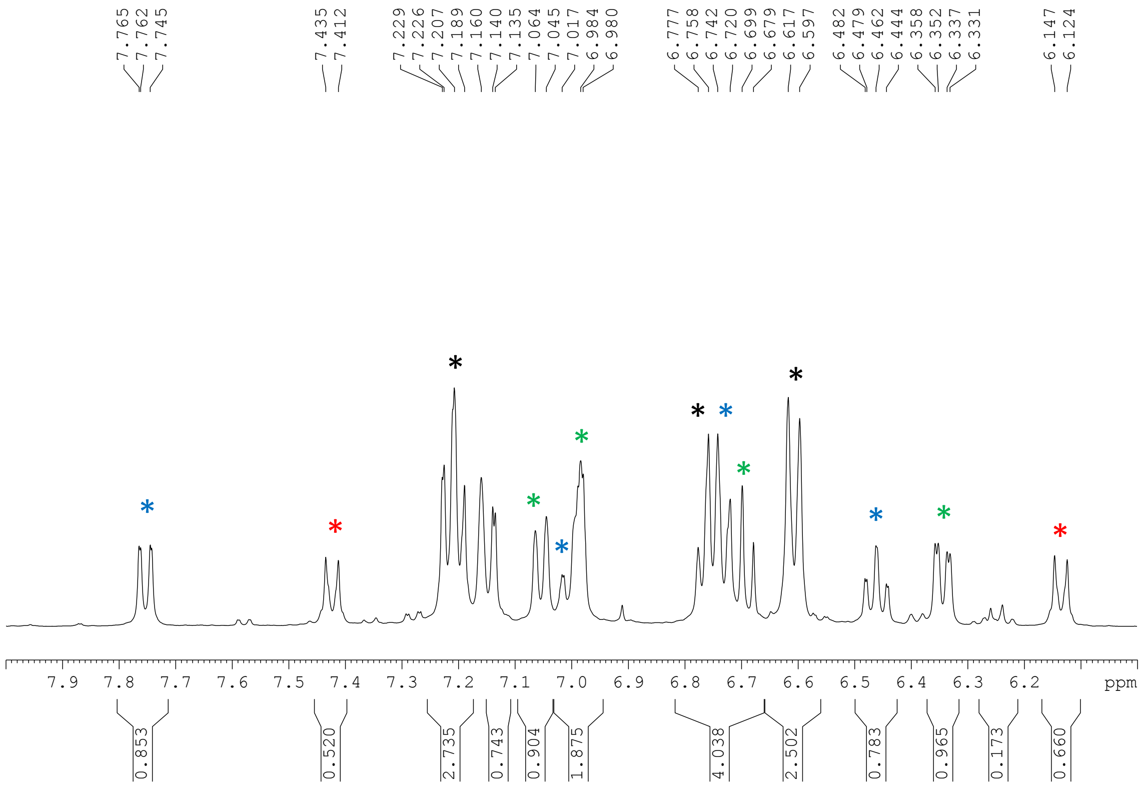

Spectrum 41. Aromatic region of ^{1}H NMR spectrum from the reaction of BuNa·TMEDA, *N*,*N*-dimethylaniline, *t*Bu_2Zn and TMP(H) with iodine following an overnight reflux in C_6D_6 solution.

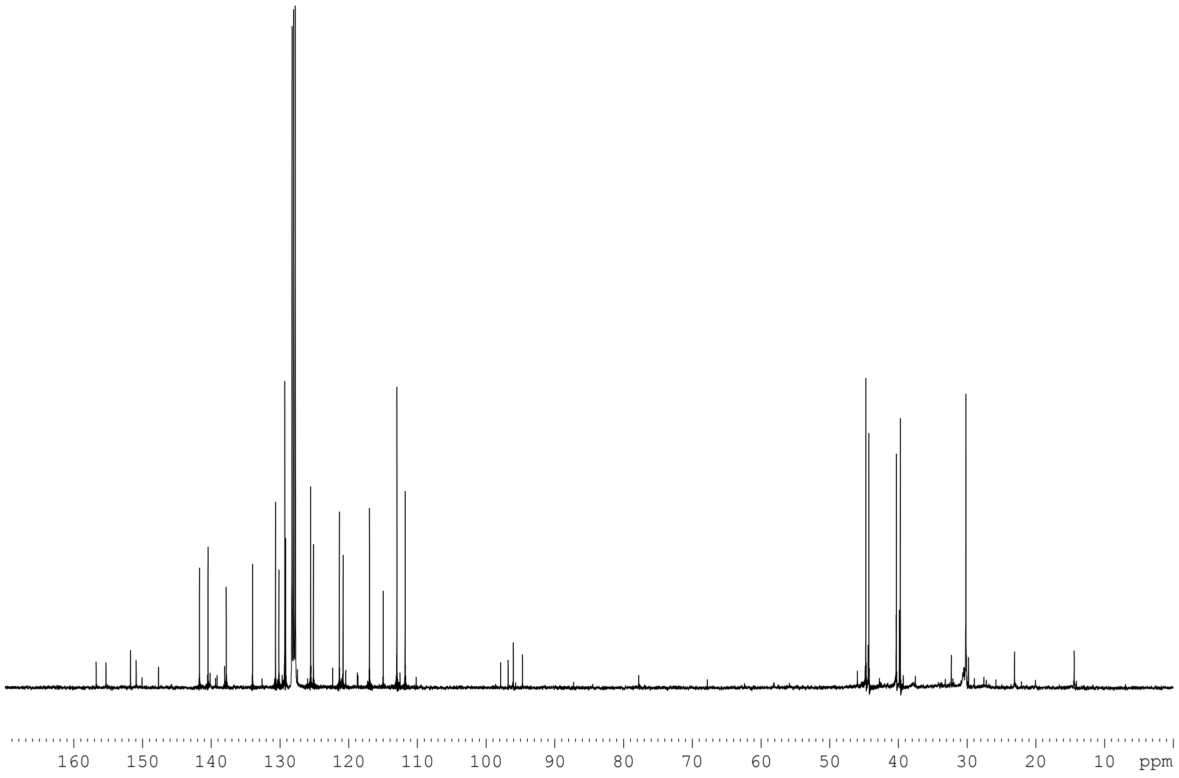

Spectrum 42. ^{13}C NMR (100.62 MHz, 300 K) spectrum from the reaction of BuNa·TMEDA, *N*,*N*-dimethylaniline, *t*Bu_2Zn and TMP(H) with iodine following an overnight reflux in C_6D_6 solution.

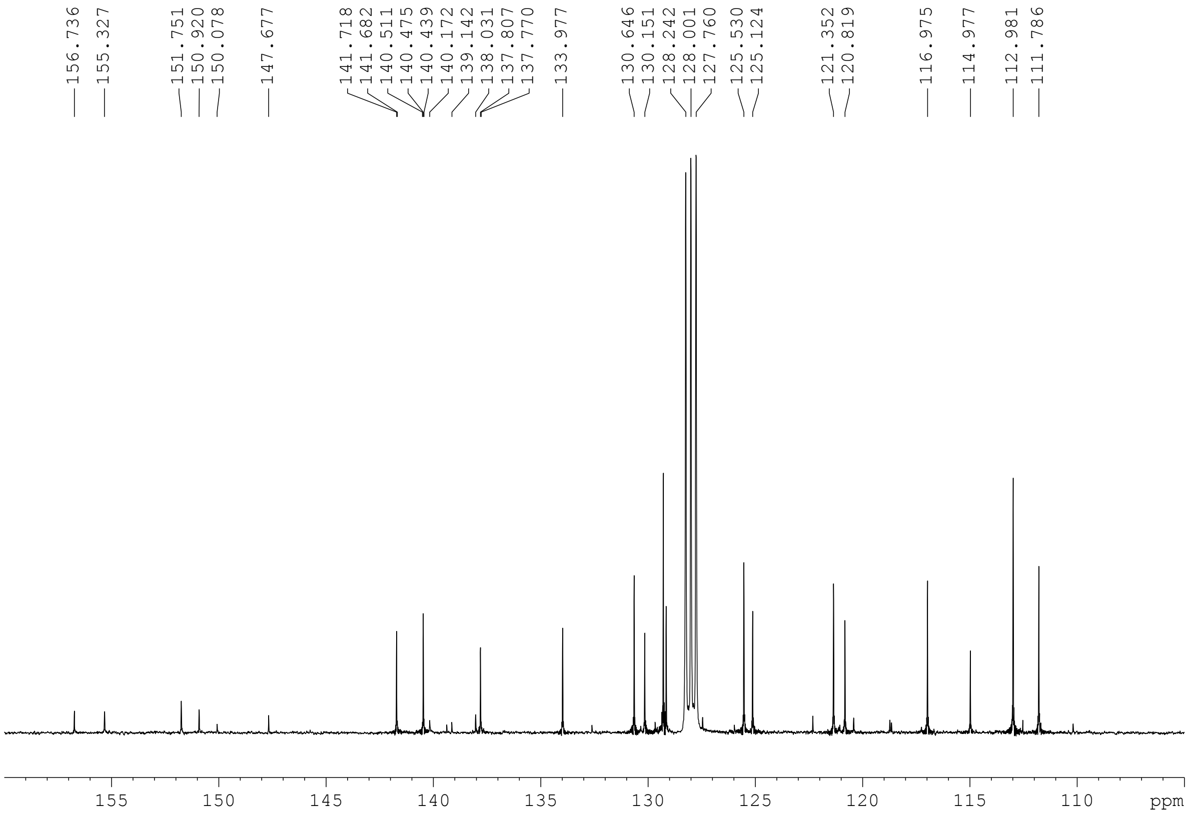

Spectrum 43. Aromatic region of ^{13}C NMR spectrum from the reaction of BuNa·TMEDA, *N*,*N*-dimethylaniline, *t*Bu_2Zn and TMP(H) with iodine following an overnight reflux in C_6D_6 solution.

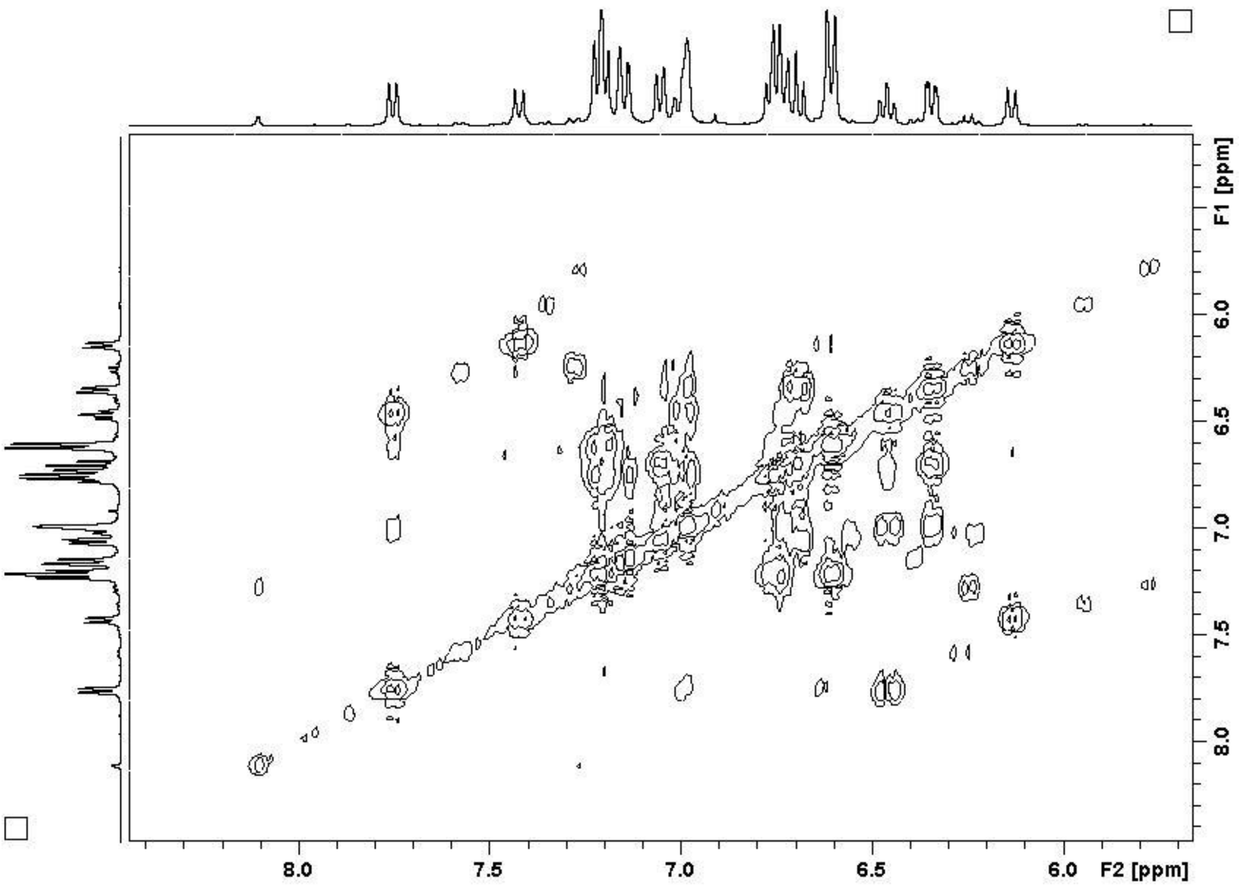

Spectrum 44. Aromatic region of ^1H-^1H COSY NMR spectrum from the reaction of BuNa·TMEDA, *N*,*N*-dimethylaniline, *t*Bu_2Zn and TMP(H) with iodine following an overnight reflux in C_6D_6 solution.

References

1. *Gaussian 03*, Revision B.0.5; Gaussian, Inc.: Pittsburgh PA, 2003.
2. Becke, A. D. *Phys. Rev. A* **1988,** *38,* 3098. doi:10.1103/PhysRevA.38.3098
3. Lee, C. T.; Yang, W. T.; Parr, R. G. *Phys. Rev. B* **1998,** *37,* 785. doi:10.1103/PhysRevB.37.785
4. McLean, A. D.; Chandler, G. S. *J. Chem. Phys.* **1980,** *72,* 5639. doi:10.1063/1.438980
5. Krishnan, R.; Binkley, J. S.; Seeger, R.; Pople, J. A. *J. Chem. Phys.* **1980,** *72,* 650. doi:10.1063/1.438955
